# Model-based identification of TNFα-induced IKKβ-mediated and IκBα-mediated regulation of NFκB signal transduction as a tool to quantify the impact of drug-induced liver injury compounds

**DOI:** 10.1038/s41540-018-0058-z

**Published:** 2018-06-11

**Authors:** Angela Oppelt, Daniel Kaschek, Suzanna Huppelschoten, Rowena Sison-Young, Fang Zhang, Marie Buck-Wiese, Franziska Herrmann, Sebastian Malkusch, Carmen L. Krüger, Mara Meub, Benjamin Merkt, Lea Zimmermann, Amy Schofield, Robert P. Jones, Hassan Malik, Marcel Schilling, Mike Heilemann, Bob van de Water, Christopher E. Goldring, B. Kevin Park, Jens Timmer, Ursula Klingmüller

**Affiliations:** 1 Division Systems Biology of Signal Transduction, German Cancer Research Center (DKFZ), Heidelberg, Germany; 2 Institute of Physics, University of Freiburg, Freiburg, Germany; 3 Division of Toxicology, Leiden Academic Centre for Drug Research, Leiden University, Leiden, The Netherlands; 4 MRC Centre for Drug Safety Science, Department of Molecular and Clinical Pharmacology, University of Liverpool, Liverpool, UK; 5 Institute of Physical and Theoretical Chemistry, Single Molecule Biophysics, Johann Wolfgang Goethe-University, Frankfurt, Germany; 6 North Western Hepatobiliary Unit, Aintree University Hospital NHS Foundation Trust, Liverpool, UK; 7 Bioquant, University of Heidelberg, Heidelberg, Germany; 8 BIOSS Centre for Biological Signalling Studies, University of Freiburg, Freiburg, Germany

## Abstract

Drug-induced liver injury (DILI) has become a major problem for patients and for clinicians, academics and the pharmaceutical industry. To date, existing hepatotoxicity test systems are only poorly predictive and the underlying mechanisms are still unclear. One of the factors known to amplify hepatotoxicity is the tumor necrosis factor alpha (TNFα), especially due to its synergy with commonly used drugs such as diclofenac. However, the exact mechanism of how diclofenac in combination with TNFα induces liver injury remains elusive. Here, we combined time-resolved immunoblotting and live-cell imaging data of HepG2 cells and primary human hepatocytes (PHH) with dynamic pathway modeling using ordinary differential equations (ODEs) to describe the complex structure of TNFα-induced NFκB signal transduction and integrated the perturbations of the pathway caused by diclofenac. The resulting mathematical model was used to systematically identify parameters affected by diclofenac. These analyses showed that more than one regulatory module of TNFα-induced NFκB signal transduction is affected by diclofenac, suggesting that hepatotoxicity is the integrated consequence of multiple changes in hepatocytes and that multiple factors define toxicity thresholds. Applying our mathematical modeling approach to other DILI-causing compounds representing different putative DILI mechanism classes enabled us to quantify their impact on pathway activation, highlighting the potential of the dynamic pathway model as a quantitative tool for the analysis of DILI compounds.

## Drug-induced liver injury: mathematical model quantifies impact of liver-damaging drugs

Drug-induced liver injury (DILI) is one of the most important obstacles during drug development. More than 1000 drugs have been identified to damage the liver, but the current test systems are poor in predicting DILI. A team of cell biologists, theoretical physicists, and clinical pharmacologists combined experimental data generated in cultured liver cells with mathematical modeling to quantify the impact of the anti-inflammatory drug diclofenac. The analysis demonstrated that diclofenac induces multiple changes in the signal transduction network activated by the tumor necrosis factor alpha (TNFα), one of the known factors to amplify liver toxicity. Data of other liver injury-causing compounds were integrated into the mathematical model and their impact was quantified, thereby demonstrating the potential use of the mathematical model for the further analysis of other compounds in order to improve DILI test systems.

## Introduction

Drug-induced liver injury (DILI) is currently one of the most important obstacles during drug development. To date, over 1000 drugs are known to cause DILI,^1^ affecting not only a restricted group of patients, but a broad range of medications and treatments.^2^ Current test systems employed by the pharmaceutical industry are poorly predictive since the underlying mechanisms are still unclear. So far, the majority of studies focused on the effects of compounds on hepatocytes, whereas the impacts of synergistic drug–cytokine interactions were rarely considered. Furthermore, due to the complexity of the impact of compounds on the dynamic behavior of the intracellular signaling network, the impacts of multiple factors have to be considered.

One of the top ten DILI-causing compounds is diclofenac (DCF), a commonly used nonsteroidal anti-inflammatory drug. DCF was shown to synergize with tumor necrosis factor alpha (TNFα) by accelerating apoptosis in primary human hepatocytes (PHH) and HepG2 cells^3,4^ by enhancing endoplasmic reticulum stress as well as oxidative stress.^5^ However, the exact underlying mode of action remained to be elucidated. TNFα signal transduction, apart from being a key mediator of inflammatory responses, plays also a major role in apoptosis. It was observed that there is a tightly regulated and very complex balance between TNFα-induced pro-survival signaling via complex I and death signaling via complex II.^6,7^ The TNFR1-Membrane-Associated Proximal Complex (complex I) is rapidly formed at the plasma membrane and is composed of the receptor itself, TRADD, RIP, TRAF2, and cIAP1, but is devoid of caspase 8 and triggers only the NFκB response but no apoptotic signaling.^6^ TNFα was reported to enhance cell death^8,9^ if the NFκB-induced inhibition of apoptotic signaling via JNK or necroptotic signaling via RIP fails.^10^ Because NFκB signal transduction is extremely complex due to a multitude of feedback regulators, it has been previously examined by applying mathematical modeling that is a powerful tool to study multifactorial and complex networks.^11–15^ Since it was proposed that the IκB kinase (IKK) signaling module is highly relevant for the temporal control of NFκB signal transduction,^16^ several mathematical models included the IKK module.^11,15,17,18^ However, a potential role of IKK in drug-induced hepatotoxicity upon inflammatory responses so far has not been addressed. IKK is a multi-protein complex composed of IKKα, IKKβ, and the regulatory IKKγ (NEMO) that phosphorylates IκB and thereby facilitates degradation of IκB inhibitors and the subsequent translocation of NFκB to the nucleus.^19,20^ The activity of the IKK is controlled by positive and negative regulatory phosphorylation cycles modulated by a network of components of the TNF receptor (TNFR) complex.^19,20^ Specifically, activation by TNFα binding to the receptor leads to the phosphorylation of two sites in the activation loop of IKKβ, which is essential for the activation of the NFκB pathway. During this highly active state, IKKβ undergoes extensive autophosphorylation at multiple sites at the C-terminus,^21^ which leads to a massive downregulation of its activity. If both the activation loop and the C-terminus are phosphorylated, IKKβ is still active, although with almost no catalytic activity. Rather, this state facilitates the recruitment of phosphatases deactivating IKK by dephosphorylation of its activation loop. This in turn creates an inactive state that is refractory to activation by TNFα.^22^ Therefore, another dephosphorylation event has to take place resulting in the non-phosphorylated state of IKKβ that is free to be re-activated by the receptor complex. In sum, the activity of IKK and thereby the activation of NFκB signal transduction is tightly regulated at the level of IKK by a four-step phosphorylation/dephosphorylation process.^22^ The latent transcription factor NFκB is regulated by the inhibitor of NFκB, IκBα, which sequesters NFκB in the cytoplasm. IκBα in complex with NFκB is a substrate for the IKK complex.^23,24^ Phosphorylation of IκBα prompts it for ubiquitination leading eventually to the degradation of IκBα and the release and nuclear translocation of NFκB. These steps provide another important regulatory mechanism to control the activation of NFκB signal transduction downstream of the TNFR complex.

To examine mechanisms responsible for the impact of DCF on TNFα-induced hepatotoxicity, we considered the highly intertwined signaling components and applied a data-based mathematical modeling approach. While the NFκB pathway has been extensively studied in the past and several dynamic pathway models exist, these mathematical models did not address the effect of DCF on the TNFα-induced NFκB signal transduction pathway. In the presented study, we generated quantitative data in the human hepatocellular carcinoma cell line HepG2 and in PHHs under standardized conditions. Jointly estimating parameters of our dynamic pathway model from the standardized data enabled us to attribute the observed alterations in the dynamics of TNFα-induced NFκB signal transduction in response to co-treatment with DCF to specific changes in parameters of the reaction network. To this end, previously established methods of model selection and L_1_ regularization^25,26^ were employed to determine the most likely interaction points between DCF and TNFα-induced NFκB signal transduction. The systematic analyses of the calibrated model revealed the interplay of IKK-mediated and IκBα-mediated regulation of the dynamics of NFκB in the nucleus and thereby explained the DCF-induced alterations of NFκB oscillations in nucleus and cytoplasm. With a mathematical model generalized to explain drug-induced IKK and IκBα regulation it was possible to quantify the impact of four additional DILI compounds (amiodarone, paracetamol, ximelagatran, and fialuridine) on TNFα-induced NFκB activation.

## Results

### Dynamic pathway model of TNFα-induced NFκB signal transduction

**Fig. 1:**
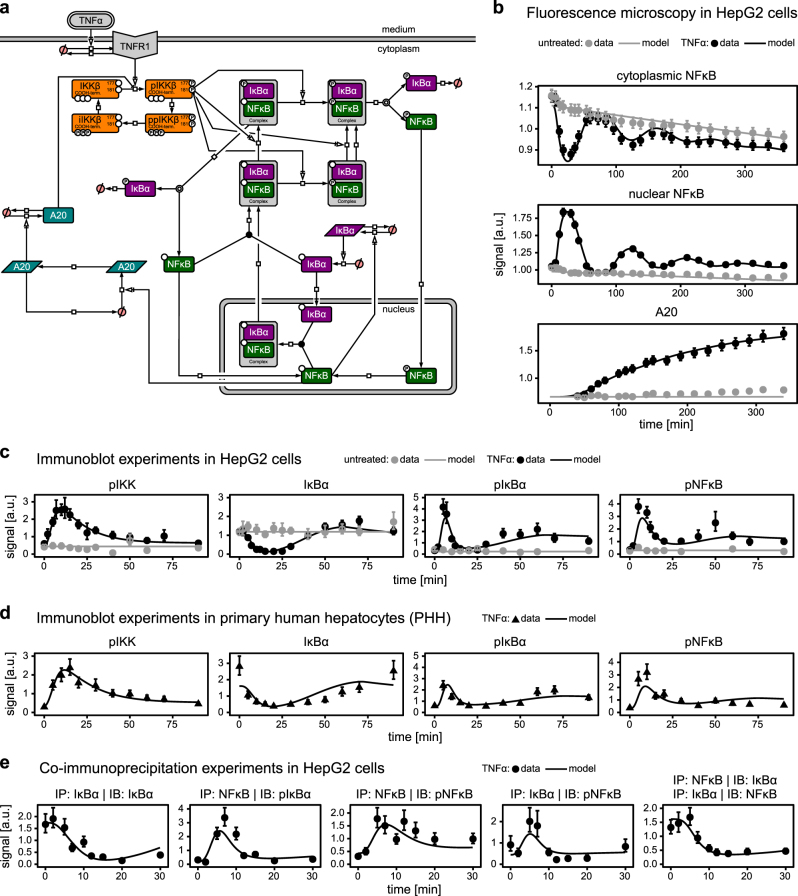
TNFα-induced NFκB signaling pathway model for HepG2 cells and primary human hepatocytes (PHH). **a** Schematic representation of the model according to Systems Biology Graphical Notation indicating the considered components and reactions. Arrows indicate biochemical reactions. Boxes with round corners symbolize proteins, parallelograms represent RNA. Slashed circles represent degradation or production. Experimental data was acquired by either live-cell fluorescence microscopy (**b**) of NFκB translocation (cytoplasm and nuclei, *n* = 3) and of A20 expression dynamics (*n* = 1) in HepG2 cells stably expressing NFκB-GFP or A20-GFP, respectively, or quantitative immunoblotting (**c**–**e**) of cytoplasmic lysates of HepG2 cells (**c**, *n* = 3–6, raw data in Figs. S8–S17) and PHHs (**d**, *n* = 3 donors, raw data in Figs. S18–S21) or immunoprecipitations (**e**, *n* = 1–3, raw data in Figs. S22–S29) treated with TNFα (10 ng/ml). Data points with 1*σ* confidence intervals computed from replicates are indicated by dots with error bars, lines indicate trajectories of the calibrated model

To develop a mathematical model for TNFα-induced NFκB signal transduction (Fig. 1a), we generated time-resolved data of several pathway components of the canonical NFκB pathway in the human hepatocellular carcinoma cell line HepG2 and in PHHs. Since it was previously reported that DCF affects the oscillatory behavior of NFκB, we first examined the dynamics of TNFα-induced nuclear translocation of NFκB and the corresponding production of the target gene A20. We utilized HepG2 cells stably expressing GFP-tagged RelA, a subunit of NFκB (further referred to as NFκB-GFP HepG2) and monitored by life cell imaging the localization of NFκB in the cytoplasm and the nucleus upon stimulation with TNFα. The NFĸB-GFP intensity was simultaneously quantified in several hundred cells, yielding an average dynamics (Fig. 1b, data points in upper two panels). As shown in Fig. S1, the single-cell dynamics of NFĸB nuclear translocation was highly consistent with the average dynamics, corroborating our approach to analyze the average data and use it for mathematical modeling. Further, we quantified the time course of A20 expression in response to TNFα stimulation in HepG2 cells stably expressing GFP-tagged A20 (A20-GFP HepG2) (Fig. 1b, data points in lower panel). These examinations showed that TNFα-induced nuclear accumulation of NFĸB reaches its first peak after 25 min followed by a second peak at 122 min. The timing of maximal accumulation of NFĸB in the nucleus mirrored the maximal reduction of NFĸB from the cytoplasm showing the expected complementary dynamics. The expression of the target gene A20 was initiated as soon as 50 min past TNFα stimulation, which is shortly after the first peak of nuclear NFĸB, and kept increasing for the entire observation time of 6 h.

To simultaneously examine the dynamics of pathway activation of multiple key components of the canonical NFκB signal transduction pathway, we used quantitative immunoblotting and determined the dynamics of IKK, IκBα, and NFκB phosphorylation and of IκBα abundance in HepG2 cells that were either stimulated with TNFα or were left untreated. Comparable experiments were performed with PHH that were treated with TNFα. As depicted in Fig. 1c for HepG2 cells and in Fig. 1d for PHH, in both cell types the key components of the canonical NFκB signal transduction pathway were phosphorylated after 10–20 min of TNFα stimulation. Likewise, the overall dynamics of pathway activation was very comparable in HepG2 cells and PHH, corroborating the use of the HepG2 cell line as model system. However, the extent of IκBα and NFĸB phosphorylation was slightly lower in PHH compared to HepG2 cells. Further, shortly after the first peak of the IKK, IκBα, and NFκB phosphorylation the cytoplasmic levels of IκBα reached the lowest values after TNFα stimulation at 20 min in both HepG2 cells and PHH. Finally, to quantify the dynamics of phosphorylation events of the NFĸB:IκBα complex, we performed co-immunoprecipitation experiments (Co-IP) in HepG2 cells that were stimulated with TNFα. Cellular lysates taken at different time points of TNFα stimulation were subjected to immunoprecipitation with antibodies recognizing IĸBα or NFĸB. In immunoblotting experiments we examined the time course of immunoprecipitated IκBα in the cytoplasm as well as the complex formation with NFκB and phosphorylated NFκB. For immunoprecipitated NFκB the dynamics of NFκB phosphorylation was monitored as well as the complex formation with IĸBα and phosphorylated IĸBα (Fig. 1e). The obtained results showed that the peak of NFκB:pIĸBα complex and of pNFκB:IĸBα slightly preceded the maximum of TNFα-induced NFκB phosphorylation.

Based on these comprehensive time-resolved examinations an ordinary differential equation (ODE)-based dynamic pathway model of TNFα-induced NFκB signaling (Fig. 1a) was developed. First, a model with a multitude of independent reaction rates involving IKK, NFĸB, and IĸBα phosphorylation as well as complex formation was established (Fig. S2). Based on this comprehensive model, the major processes contributing to the signaling pathway were identified by inspecting all reaction fluxes (Fig. S3). To describe the upstream processes leading to the activation of the NFκB pathway, we included four IKK states in the dynamic pathway model that are denoted as IKK, phosphorylated (p) IKK, multiply phosphorylated (pp) IKK, and inactive (i) IKK. These states are based on sequential events induced by TNFα: first, the kinase domain is phosphorylated at the activation loop leading to full kinase activity (pIKK). Second, the C-terminal serines are phosphorylated, while the kinase domain is still phosphorylated (ppIKK). In the ppIKK state, IKKβ has very low catalytic activity. Finally, IKKβ is inactivated (iIKK) due to dephosphorylation of the activation loop and needs a recovery time before it can be activated again (IKK). Because the antibody applied for measuring the phosphorylation of IKK is detecting the phosphorylation in the activation loop, the immunoblotting measurements represent both pIKK and ppIKK. However, according to our flux analysis, only pIKK has an impact on IĸBα and NFĸB phosphorylation within the complex. Because phosphorylation of IĸBα triggers the dissociation of the NFκB:IκBα complex, this results in our model in free NFĸB, either phosphorylated or not. The reaction fluxes showed that both states are necessary and fulfill a different purpose. Inspecting the gain terms of the NFĸB:IĸBα complex we found that complex formation in the cytoplasm of unphosphorylated NFĸB with *de novo* produced IĸBα is almost as strong as the supply by complexes exported from the nucleus. Another finding from the analysis of the reaction fluxes was that the newly synthesized A20 protein, although being known to be a negative feedback on TNFα-mediated IKK activation, has a negligible inhibitory impact on IKK. Based on the analysis of the reaction fluxes a much reduced mathematical model was developed. Further reduction steps were introduced based on the profile likelihood method. The profile likelihood analysis pointed out that several estimated parameters, e.g., nuclear NFĸB dephosphorylation, complex formation and export, are likely to operate on a faster timescale than other reactions, and therefore cannot be constrained by upper confidence bounds based on the data. Finally, the combination of flux and profile likelihood analysis led to a fully identifiable core model capable of describing the dynamics of TNFα-induced activation of NFĸB signal transduction in HepG2 cells (Fig. 1b, c, e). A list of all reaction equations and parameter values is provided in Supplementary Material 1 (Tables S5–S7).

### Identification of DCF targets in the TNFα-induced NFκB signal transduction pathway

**Fig. 2:**
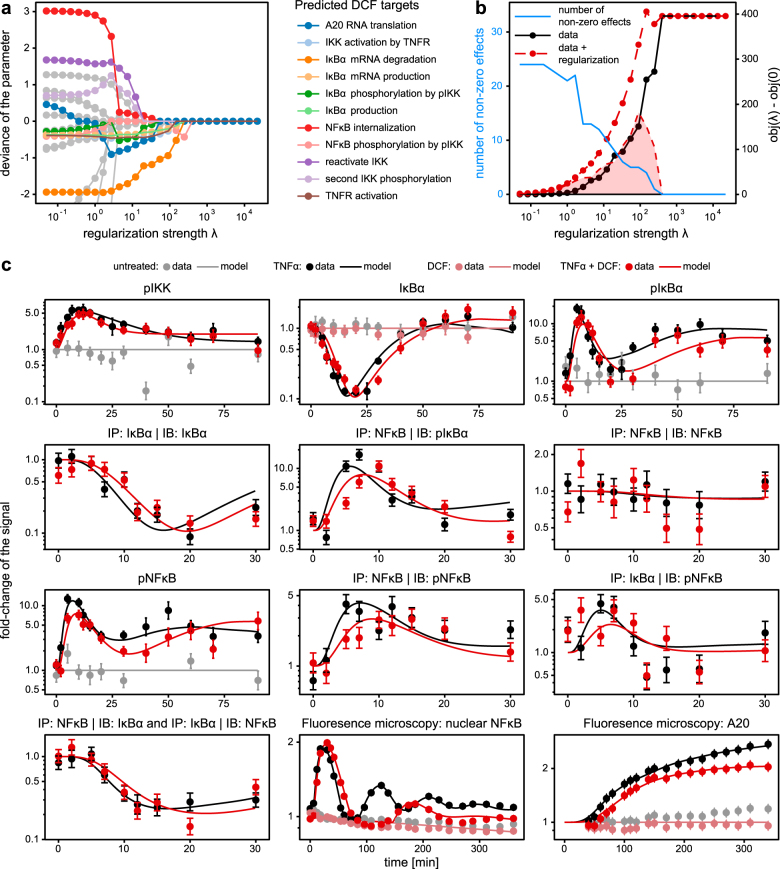
Identification of diclofenac effects. **a** Increasing regularization strength *λ* forces parameter differences between the mathematical model describing TNFα-stimulated control data and the model with modified rates based on the data of TNFα and DCF-treated cells to vanish. The eleven parameters disappearing last are indicated as colored paths, early disappearing effects are shown in gray. **b** Every vanishing effect reduces the model complexity by one, shown in blue. Accordingly, the fit quality decreases indicated by larger contributions from the data points to the objective value, indicated in black. The regularization value, shaded area, reaches its maximum when regularization strength and model complexity balance out. Regularization and data contribution sum up to the total objective value, shown as red line. **c** The core model with a total of seven diclofenac-specific rate parameters was fitted to the experimental data. Data points with 1*σ* confidence intervals computed from replicates are indicated by dots with error bars, model fits are shown as solid lines. Experimental data was acquired by either live-cell fluorescence microscopy of NFκB translocation (cytoplasm and nuclei, *n* = 3) and of A20 expression dynamics (*n* = 1) in HepG2 cells stably expressing NFκB-GFP or A20-GFP, respectively, or by quantitative immunoblotting of cytoplasmic lysates (*n* = 3–6, raw data in Figs. S8–S17) or immunoprecipitations (*n* = 1–3, raw data in Figs. S22–S29) of HepG2 cells, either untreated, treated with TNFα alone (10 ng/ml), treated with diclofenac (500 µM) alone or co-treated with TNFα (10 ng/ml) and diclofenac (500 µM)

To assess the impact of DCF on the TNFα-induced NFκB signal transduction pathway, we quantified the dynamics of the respective components in HepG2 cells in the presence or absence of DCF using quantitative immunoblotting and live-cell imaging (Fig. 2c, symbols). To select a suitable DCF dose, the impact of increasing doses of DCF on TNFα-induced IκB phosphorylation was examined (Fig. S7). In these experiments a major reduction of TNFα-induced IκB phosphorylation was observed upon treatment with 500 μM DCF. Since in a previous publication a substantial enhancement of DCF toxicity was observed upon treatment with 500 μM DCF and TNFα,^5^ this DCF concentration was selected for all measurements. DCF was added to the cells 30 min prior to stimulation with TNFα. The most prominent effects of co-treatment of TNFα and DCF were a delay of the second NFĸB peak in the nucleus and a reduced A20 production. Specifically, in the presence of TNFα and DCF the first peak of nuclear NFĸB was delayed by 8 min and slightly exceeded the maximum level observed for TNFα treatment alone. The phase of low NFĸB levels was prolonged in TNFα and DCF co-treated HepG2 cells relative to TNFα treatment alone and the second peak of nuclear NFĸB was delayed by 56 min. Also the levels of nuclear NFĸB were lower for TNFα and DCF co-treated cells. These effects were preceded by delayed and reduced concentrations of IĸBα, phosphorylated IĸBα, and NFĸB in the cytoplasm after 30 min of co-stimulation.

To systematically identify putative targets of DCF in the TNFα signaling network, we utilized the established core model of TNFα-induced NFκB signal transduction to analyze the time-resolved live-cell imaging and immunoblotting data of the TNFα-stimulated HepG2 cells that were either co-treated with DCF or not (Fig. 2c, data points). For simultaneous parameter estimation in TNFα and DCF co-treated or untreated cells, L_1_ regularization was used to gradually reduce the number of hypothesized interactions between DCF and the reaction network. For different values of the regularization strength *λ*, the regularized objective function *l*_*λ*_(*k*, Δ_*k*_) was optimized with respect to the original parameters *k* and the DCF-specific parameters Δ_*k*_. In Fig. 2a, the estimated Δ-values are shown as function of the regularization strength *λ*. With increasing *λ*, more and more parameters were reduced to the baseline indicating that their effect is estimated to be negligible. The 11 parameters that were reduced last to the baseline are indicated by colored lines. These parameters are retained, since they are most essential to maintain a good fit between data and model when the number of non-zero difference parameters is reduced. The decline of non-zero parameters is shown as a blue line in Fig. 2b. For comparison, the objective value with and without the regularization contribution are shown in red and black, respectively. The shaded area, i.e., the regularization contribution, indicates that for high enough regularization strength the number of non-zero Δ-parameters is decreased to an extent that the regularization contribution vanishes. However, at this point the fit between data and model deteriorated by 400 in terms of twice the negative log-likelihood compared to the model with 24 Δ-parameters. According to a likelihood ratio test, deterioration of the likelihood becomes highly significant (*p* < 0.001) when the penalty strength passes the threshold of *λ* = 10, corresponding to 11 non-zero effects and a difference of 34 in terms of twice the negative log-likelihood.

The list of non-zero effects revealed that the major targets of DCF are IKK activation and IĸBα production and degradation. In addition, the production of A20 appeared to be directly affected. Of note is that some of the curves depicted in Fig. 2a were overlapping. For example, IKK activation by the TNFR1 (light blue) and TNFR1 activation (brown) cannot be distinguished in the plot. A change of either rate parameter had the same effect on the model response and therefore these parameters were considered as not independent. Based on the obtained results, seven DCF-specific reaction rates were identified to be independent: the activation of IKK by TNFα, the recovery of iIKK, the phosphorylation of pIKK, the phosphorylation of IĸBα by pIKK, the production and degradation of IĸBα mRNA, and, finally, the translation of A20 mRNA were introduced into the model yielding the TNFα signaling DCF model. As a result, the data from HepG2 cells treated either with TNFα alone or in with a combination of TNFα and DCF were successfully described by the same model topology (Fig. 2c, solid lines).

### Impact of DCF on TNFα-induced NFκB signal transduction in PHHs

**Fig. 3:**
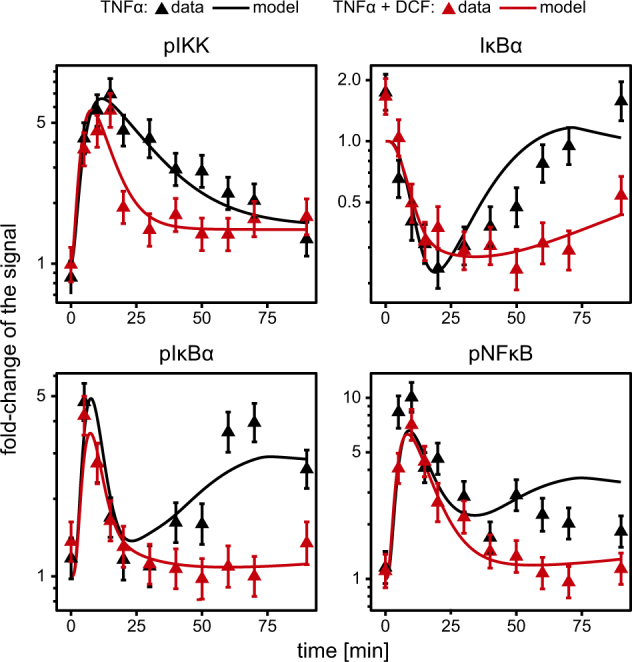
Primary human hepatocytes data and fit. Experimental data was acquired by quantitative immunoblotting of cytoplasmic lysates of primary human hepatocytes treated with either TNFα alone (10 ng/ml) or with TNFα (10 ng/ml) and diclofenac (500 µM). Data points with 1*σ* confidence intervals computed from independent donors (*n* = 3, raw data in Figs. S18–S21) are indicated by triangles and error bars. Only the model parameters specific for DCF treatment, observation-specific scaling and offset parameters and a global time-scale parameter were adjusted to predict the correct IKK, IκBα, and NFκB profiles

To verify that the model-identified impact of DCF on TNFα-induced NFκB signal transduction holds true in primary liver cells, we tested the TNFα signaling DCF model on data from freshly isolated PHHs, stimulated with TNFα in the presence or absence of DCF (Fig. 3). Since the availability of PHH is very limited, we focused our analysis on immunoblotting experiments and selected time points that were most informative in the analysis of the DCF effects on TNFα-induced NFκB signal transduction in HepG2 cells. As demonstrated in Fig. 1, the overall dynamics of pathway activation in PHH was highly consistent with the pathway activation dynamics observed in HepG2 cells, but appeared to be slower than in HepG2 cells. To consider this difference in timing, we introduced a global time-scale parameter affecting all reaction rates in the mathematical model. To account for the difference in signal strength and background in the immunoblotting experiments performed with PHH, observation parameters such as signal scale and offset were independently estimated. Furthermore, although we assume that the mechanism by which DCF acts on TNFα signaling in PHH is very comparable to HepG2 cells, the strength of DCF may vary in the primary cells. Taken together, a time-scale parameter, scaling and offset parameters, as well as the DCF-specific parameters were estimated from the PHH data, whereas all other parameter, i.e., reaction rates of the NFκB signaling network were unchanged. The resulting model trajectories shown in Fig. 3 are in agreement with the experimental data, confirming that our mathematical model can also be applied to data generated in primary cells. Furthermore, experimental data and mathematical model simulation demonstrated that also in PHH DCF has a strong and early impact on TNFα-induced NFκB signal transduction. In cells co-treated with TNFα and DCF, the activation of IKK was more transient and recovery of IκBα, phosphorylated IκBα, and phosphorylated NFκB were much reduced compared to TNFα-treated cells. These observations underpin the broad applicability of the developed model to quantitatively assess the impact of DCF on TNFα-induced NFκB signal transduction.

### Impact of DCF on the TNFα/TNFR1 interaction in HepG2 cells

**Fig. 4:**
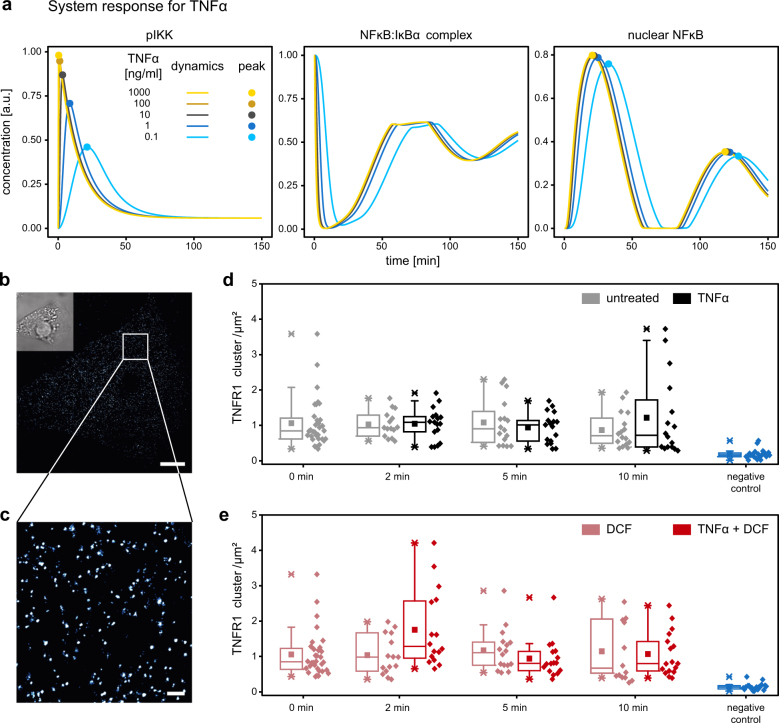
Impact of DCF on TNFα and TNFR1 interaction. **a** Concentration time courses of pIKK, non-phosphorylated NFκB–IκBα complex in the cytoplasm and free, non-phosphorylated NFκB in the nucleus were simulated for TNFα concentrations between 0.1 and 1000 ng/ml. In the experiment 10 ng/ml were applied. The first maximum of pIKK and the first two maxima of nuclear NFĸB are indicated by dots. The different TNFα levels have a large effect on the peak height and position of pIKK relative to NFκB–IκBα complex and nuclear NFκB that remain almost unchanged for higher TNFα concentrations and exhibit a minorly reduced and delayed response for lower TNFα concentrations. **b**–**e** dSTORM imaging of TNFR1 in the plasma membrane of HepG2 cells. **b** Respresentative dSTORM image of TNFR1 labeled via indirect immunocytochemistry on the plasma membrane of HepG2 cells (inset: brightfield image) (scale bar 5 µm). **c** Magnification of the inset indicated in **b** (scale bar 500 nm). **d** Number of TNFR1 clusters on the cell membrane at the indicated time points before and after induction with TNFα (gray: non-stimulated cells, black: TNFα-stimulated cells, blue: negative control with secondary antibody only). **e** Number of TNFR1 clusters for cells pre-treated with DCF (light red) followed by induction with TNFα (red) (number of cells measured under each condition *n* ≥ 13). Stimulation with TNFα shows no significant change to the according unstimulated population. Pre-treatment with DCF shows no significant changes in regard to untreated cells. Box plots in **d** and **e** indicate the median (line in box), lower and upper quartile (box), the mean value (square), and the data range (asterisks). Whiskers represent 1.5× the interquartile distance. Statistical analysis was performed using the Kolmogorov–Smirnov test with significance level *α* = 0.05

The L_1_ regularization approach identified seven reaction rates in the signaling network as possible targets of DCF, including IKK activation. In principle, the predicted reduced IKK activation in TNFα and DCF co-treated cells could be mediated by a direct interference of DCF with TNFα binding to its receptor resulting in reduced TNFα levels available for the initiation of signal transduction. To explore this mechanism and evaluate if reduced TNFα levels alone could explain the observed effects of DCF on TNFα-induced NFĸB signal transduction, we utilized our TNFα signaling DCF model and simulated the dynamics of key components of the signaling pathway, pIKK, NFĸB:IĸBα complex, and nuclear NFĸB in response to different TNFα concentrations. We used the experimentally applied 10 ng/ml TNFα as reference and predicting with the model the dynamics for higher (100 and 1000 ng/ml) and lower (1 and 0.1 ng/ml) TNFα doses (Fig. 4a). The model trajectories showed that increasing TNFα concentrations above the experimentally applied 10 ng/ml of TNFα resulted in an increase of IKK phosphorylation levels but not in an increase in the peak height of nuclear NFĸB. This effect can be understood from the predicted dynamics of the NFĸB:IĸBα complex concentration. Already the reference TNFα concentration of 10 ng/ml lead to the dissociation of almost all NFĸB:IĸBα complexes. Even for TNFα concentrations as low as 0.1 ng/ml, more than 90% of all NFĸB:IĸBα complexes dissociated. Accordingly, the peak height of nuclear NFĸB showed only a small variation between different TNFα concentrations. Interestingly, the model simulations revealed only a minor difference in the time of the first and the second peak of nuclear NFĸB upon lowering the TNFα concentration (dots in Fig. 4a). These predictions were in contrast to the observed impact of DCF (Fig. 2c) characterized by prolonged low levels of NFĸB in the nucleus followed by a much delayed second nuclear peak of NFĸB. Therefore, a reduction in the effective TNFα dose due to interference of DCF with TNFα binding to its receptor apparently would not be sufficient to explain the experimentally observed more transient IKK activation and the shift in the second peak of nuclear NFκB. To corroborate these model-based insights, we analyzed by single-molecule localization microscopy (SMLM) whether DCF affects the number of TNFR1 complexes present on the plasma membrane of HepG2 cells (Fig. 4b–e). HepG2 cells were stimulated with TNFα for 2, 5, and 10 min or were left unstimulated. The TNFR1 cluster density at the cell membrane of unstimulated HepG2 cells showed a broad distribution with a mean value of 1.1 ± 0.1 clusters per µm² (Fig. 4d, Table S10), which is comparable to previous results obtained for HeLa cells.^27^ The stimulation of HepG2 cells with TNFα did not significantly change the density of TNFR1 clusters at the cell membrane at the indicated time points. Similarly, co-treatment with DCF did not induce a change in the number of TNFR1 clusters (Fig. 4e, Table S10). Additionally, we investigated the size of TNFR1 clusters by analyzing SMLM data using the cluster algorithm DBSCAN.^28^ We found a homogeneous distribution of TNFR1 cluster size in unstimulated cells with a mean radius of 28.7 ± 0.4 nm. The cluster radius was not altered upon stimulation with TNFα and co-treatment with DCF (see Table S10) indicating that DCF neither changes the number nor the size of TNFR1 clusters in the plasma membrane of HepG2 cells. Together with the model-based insights these results suggest that DCF does not impact the TNFR1, but rather targets intracellular reactions of the NFκB signaling pathway and thereby shifts the positioning and height of the second peak of nuclear NFκB.

### Mathematical model-based exploration of mechanism explaining the impact of DCF on TNFα-induced NFκB signal transduction

To identify mechanisms explaining the impact of DCF on intracellular TNFα-induced NFκB signal transduction, we again utilized our TNFα signaling DCF model to theoretically examine the dynamic behavior of the concentration of unobserved species in response to TNFα stimulation compared to the co-treatment with TNFα and DCF.

**Fig. 5:**
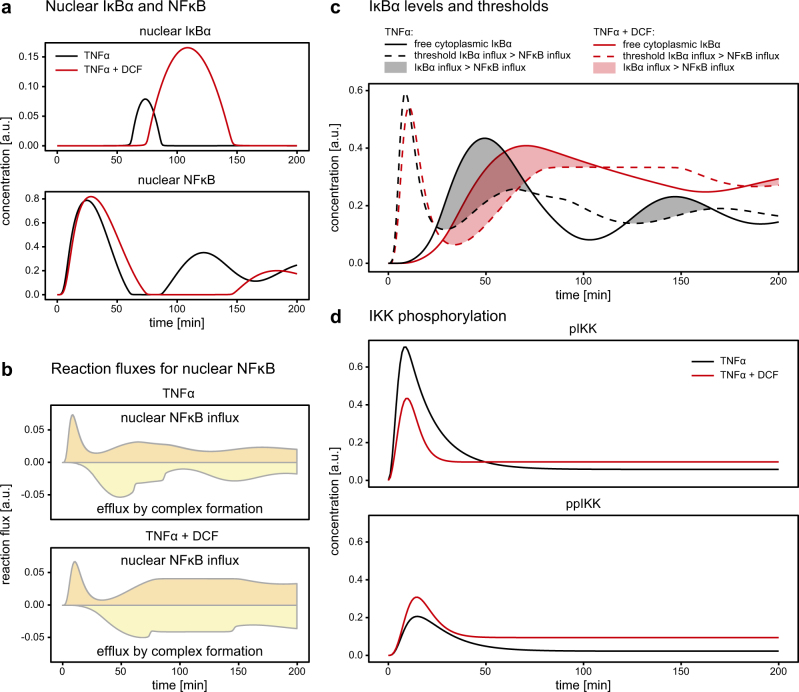
Model prediction of DCF-induced changes in the NFκB pathway. **a** Model-predicted time course of nuclear IκBα and of NFκB in response to TNFα (black line) and upon TNFα and DCF co-treatment (red line). **b** Model simulation of the time course of the influx to the state of nuclear NFκB (brown) and efflux due to complex formation (yellow) in response to TNFα stimulation or upon co-treatment of TNFα and DCF. **c** Model-predicted time course of cytoplasmic IκBα response to TNFα (black solid line) and upon TNFα and DCF co-treatment (red solid line). Depending on the cytoplasmic IκBα concentration, nuclear NFκB influx is lower or higher than IκBα influx. The threshold concentration at which IκBα influx exceeds the NFκB influx is shown as dashed black line for TNFα and as dashed red line for TNFα and DCF. Shaded areas indicate phases of higher IκBα influx leading to IκBα accumulation in the nucleus. **d** Model-predicted time course of active IKK (pIKK) and of inactive IKK (ppIKK) for time points up to *t* = 50 min. The dynamics of pIKK and ppIKK in the presence of TNFα alone (black) and the presence of TNFα and DCF (red) are displayed. The higher pIKK level for DCF treatment at late time points is reflected in the crossing of IκBα thresholds at *t* = 60 min

The comparison of nuclear NFĸB and IĸBα profiles in response to both treatments indicated that nuclear accumulation of IĸBα and low levels of NFĸB in the nucleus are highly correlated (Fig. 5a). One explanation could be that the lack of nuclear NFĸB prevents complex formation between NFĸB and IĸBα and as a consequence IĸBα accumulates in the nucleus. To test this hypothesis, we examined the reaction fluxes controlling the amount of NFĸB in the nucleus: nuclear NFĸB import (positive flux) and complex formation between NFĸB and IĸBα leading to export of NFĸB to the cytoplasm (negative flux) (Fig. 5b). If the lack of nuclear NFĸB prevented complex formation, the fluxes would drop to zero at some time points due to the absence of NFκB. However, according to the prediction of the model, the influx of NFκB into the nucleus and the efflux upon complex formation are different from zero, even if the amount of NFκB is very small. This indicated that any NFĸB molecule transported into the nucleus will, at the same rate, leave the nucleus in a complex with IĸBα. Since the maximal NFĸB flux is limited, the accumulation of nuclear IĸBα can be traced back to the level of IĸBα in the cytoplasm. If the level rises above a certain threshold, the NFĸB flux is insufficient to counteract the increased IĸBα import and IĸBα will accumulate in the nucleus.

Because the phosphorylation of IĸBα by pIKK as well as the production and degradation of IĸBα mRNA were among the DCF-targeted reaction rates, we investigated IκBα levels in the cytoplasm (Fig. 5c). Because the transport flux of IκBα into the nucleus is proportional to the levels of IκBα in the cytoplasm, the threshold of when the IκBα influx balances out the NFκB influx can be computed (dashed lines in Fig. 5c). The shaded areas in Fig. 5c mark the time frames where cytoplasmic IĸBα levels exceed the computed threshold and more IĸBα than NFĸB is transported to the nucleus, and IĸBα is expected to accumulate in the nucleus.

On the one hand, the comparison between TNFα and DCF co-treated and DCF-untreated cells showed that the IĸBα production and degradation are reduced by DCF co-treatment leading to a slower but prolonged IĸBα aggregation in DCF co-treated cells. Accordingly, IĸBα exceeded the threshold slightly later and intersected it again 110 min after TNFα and DCF co-stimulation, corresponding to the position of the maximum concentration of nuclear IĸBα (Fig. 5a). On the other hand, the influx of NFĸB to the nucleus depends on its release from the NFĸB:IĸBα complex, and consequently depends directly on the pIKK levels. We hypothesized that lower levels of IKK activation would reduce the IĸBα threshold concentration of when the IĸBα influx into the nucleus exceeds the NFĸB influx. An extended phase of low concentrations of nuclear NFκB would be the consequence. Therefore, to further examine consequences of the DCF impact on IKK, we predicted the experimentally unobserved concentrations of active pIKK and inactive ppIKK by our dynamic pathway model. The estimated parameters suggested that DCF accelerates the transition from active pIKK to inactive ppIKK (Fig. 5d). Accordingly, the IĸBα threshold shown in Fig. 5c was lowered in TNFα and DCF co-treated cells, at least during the first hour. At later time points, pIKK in TNFα and DCF co-treated cells was predicted to be above the levels of pIKK in TNFα-treated cells. The model predicted that co-treatment with DCF reduces the peak height of pIKK by around 40% (Fig. 5d), whereas the relative change of experimentally measured IKK phosphorylation was much smaller. The discrepancy can be explained by our four-state IKK model with two IKK states phosphorylated at the activation site, active pIKK, and inactive ppIKK, which cannot be distinguished by the antibody-based measurement. Therefore, we propose that DCF decreases pIKK and increases ppIKK, resulting in only a small change in the sum of pIKK and ppIKK.

These results indicate that the experimentally observed shift of the position of the second peak of nuclear NFκB in response to co-treatment with DCF is the integrated result of two regulatory mechanisms: the reduced IκBα production and degradation rates prolong the presence of elevated levels of IκBα in the cytoplasm and reduced pIKK levels lower the threshold of when cytoplasmic IκBα levels exceed the value necessary to compensate the NFĸB influx into the nucleus.

### Model-based analysis of the impact of additional DILI compounds on TNFα-induced NFκB signal transduction

**Fig. 6:**
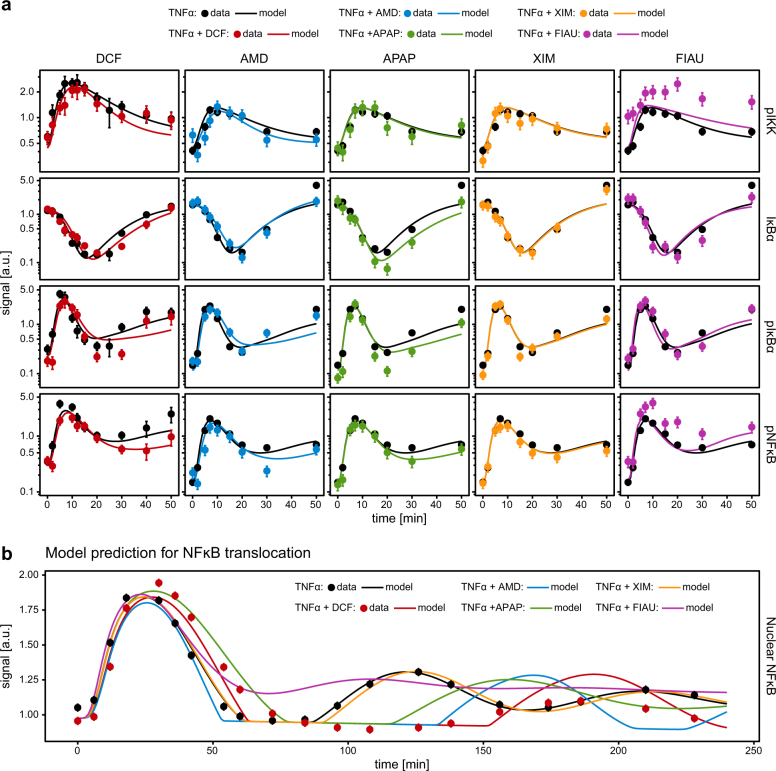
Application of the dynamic pathway model to additional DILI compound data. **a** Impact of four selected compounds, amiodarone (AMD, 35 µM), paracetamol (APAP, 10 mM), ximelagatran (XIM, 800 µM), and fialuridine (FIAU, 500 µM), on cytoplasmic IκBα, pIκBα, pIKK, and pNFĸB levels were measured by quantitative immunoblotting in HepG2 cells treated with either TNFα alone (10 ng/ml) or TNFα (10 ng/ml) and the respective compound (*n* = 3–4, raw data in Figs. S30–S45). Data points with 1*σ* confidence intervals are shown as dots and error bars in different colors for different compounds, black indicating the TNFα treatment alone. The model fit for the reduced compound-impact model is shown as solid line. Model calibration involves only experiment-specific observation parameters and the two compound-specific effect parameters *α* and *β*. **b** The model-predicted dynamics of nuclear NFκB for a time period of 4 h based on the short-term measurements per compound are shown as solid lines. Data points with 1*σ* confidence intervals for TNFα-treated cells and cells co-treated with TNFα and DCF are shown as dots with error bars

After identifying IKK phosphorylation and IĸBα production/degradation as major regulatory mechanisms affected by DCF, additional compounds known to cause DILI including amiodarone (AMD), paracetamol (APAP), fialuridine (FIAU), and ximelagatran (XIM) were tested. These compounds, which were previously reported^2^ to represent different mechanisms of DILI, were examined regarding their impact on TNFα-induced NFκB signal transduction. To select most suitable DILI compound concentrations, we examined, as shown in Fig. S7, the impact of increasing concentrations of AMD, APAP, and XIM on the phosphorylation of IκB 5 min after co-stimulation with TNFα. Increasing AMD concentrations resulted in rapid reduction of IκB phosphorylation that was not further increased by high compound concentrations. In line with a recent publication that reported enhanced AMD toxicity upon co-treatment with of 35 µM AMD and TNFα, we employed a concentration of 35 µM AMD in our experiments. For APAP no impact on TNFα-induced IκB phosphorylation was observed at reasonable APAP concentrations and therefore a concentration of 10 mM APAP was selected in line with a multi-center ring trial.^29^ Since XIM had no effect on TNFα-induced IκB phosphorylation, we decided based on literature information to apply a concentration of 800 µM XIM in our experiments. Finally, for FIAU we observed in test experiments elevated IKKβ, phosphorylation at 20 min of TNFα treatment. Whereas we observed no major impact of FIAU treatment at 20 min on IκB phosphorylation, IKKβ phosphorylation was increased. We chose a concentration of 500 µM FIAU, which caused approximately a doubled IKKβ phosphorylation signal intensity compared to the control. For each of these compounds, total IĸBα, phosphorylated IĸBα, phosphorylated IKK, and phosphorylated NFĸB in the cytoplasm were measured by quantitative immunoblotting (Fig. 6a, data points). For co-treatment experiments with the selected compounds and TNFα, HepG2 cells were incubated with the compounds for 30 min prior to stimulation with TNFα and then lysed at the indicated time points during an observation time of up to 50 min. To facilitate the analysis of the obtained time course data by our TNFα signaling DCF model, the key mechanisms identified for the impact of DCF were further condensed to describe the possible impact of compounds by only two parameters *α* and *β*. The parameter *α* is associated with IKK phosphorylation. The phosphorylation rates of IKK and pIKK are expressed as $k_{{\mathrm{IKK}} \to {\mathrm{pIKK}}}^ \ast$ = $\alpha ^{ - 1} \cdot k_{{\mathrm{IKK}} \to {\mathrm{pIKK}}}$ and $k_{{\mathrm{pIKK}} \to {\mathrm{ppIKK}}}^ \ast$ = $\alpha \cdot k_{{\mathrm{pIKK}} \to {\mathrm{ppIKK}}}$, respectively. Accordingly, larger values of *α* shift the IKK phosphorylation from pIKK to ppIKK. The parameter *β* is associated with the production and degradation rates of IĸBα. The corresponding rates are modified on the mRNA level, $k_{0 \to {\mathrm{mI\kappa B\alpha }}}^ \ast$ = $\beta ^{ - 1} \cdot k_{0 \to {\mathrm{mI\kappa B\alpha }}}$ and $k_{{\mathrm{mI\kappa B\alpha }} \to 0}^ \ast$ = $\beta ^{ - 1} \cdot k_{{\mathrm{mI\kappa B\alpha }} \to 0}$. Accordingly, larger values of *β* slow down IĸBα mRNA production and degradation for fixed equilibrium constant. The parameters *α* and *β* are compound-specific, i.e., they are determined for each compound separately.

First, this simplified model was employed to estimate *α* and *β* from the original data obtained for TNFα and DCF co-stimulation. Only the time points and targets that were also measured for the selected compounds were used. Based on these estimates, the dynamics of nuclear NFĸB was predicted (Fig. 6b). The results revealed that the simplified model is capable to correctly predict, based on cytoplasmic measurements restricted to the first 50 min of stimulation, the position of the second peak of nuclear NFκB at 180 min. The information necessary for this prediction is encoded in the slight time shift of IKK phosphorylation and the decelerated IĸBα dynamics.

Assuming that AMD, XIM, APAP, and FIAU might affect the regulatory mechanisms in a similar way, we used the time course data obtained for these compounds to estimate the compound-specific values for *α* and *β*. The analysis revealed that the mathematical model is capable to describe the time course data for AMD, XIM, and APAP, but not for FIAU (Fig. 6a). Experimental data and model trajectories showed that AMD shifted the TNFα-induced IKK phosphorylation similar to DCF, whereas the effect on IĸBα was barely noticeable. The reduced IKK phosphorylation resulted in an extended time frame of low concentrations of nuclear NFĸB (Fig. 6b). In contrast, APAP showed a strong effect on IĸBα but almost no effect on IKK phosphorylation. Consequently, the second NFĸB peak was delayed but the time frame of low nuclear NFĸB concentration was not as extended as for AMD. Finally, XIM neither showed effects on IĸBα nor on IKK phosphorylation. Accordingly, the predicted nuclear NFĸB profile resembled the TNFα-stimulated control data. Because both AMD and APAP seemed to exhibit only one of the identified drug-induced effects, the delay of the TNFα-induced NFĸB peak was not as strong as for DCF. The measurements taken from TNFα and FIAU co-treated cells showed effects on the basal IKK and NFĸB phosphorylation that were not observed for any of the other compounds. These data indicated that an additional mechanism is involved in the case of FIAU that is not covered by our mathematical model.

For DCF the simplified mechanism of action allowed to estimate drug-specific parameters that enable the prediction of the dynamics of nuclear NFκB. Within the analysis of likely interactions of DCF with the NFκB pathway, we found that DCF apparently directly modifies the production of A20 (direct impact), whereas the delayed dynamics of nuclear NFκB (mediated impact) alone is not sufficient to correctly describe the observed A20 dynamics. An evaluation of the possible impact of the other DILI compounds on the cytoplasmic concentration of A20 is presented in Fig. S6, and compares by model simulations a possible direct and an indirect mechanism. The obtained results indicated that AMD is likely to affect the A20 dynamics in a similar way as DCF. APAP and XIM have a smaller inhibitory effect on the A20 concentration in the cytoplasm and FIAU could even increase the production of A20.

In summary, our model-based approach enables us to quantify the impact of DILI compounds on TNFα-induced NFκB signal transduction using IKK phosphorylation and IĸBα production/degradation as major characteristics of DILI compounds within the TNFα-induced NFκB signaling pathway.

## Discussion

DILI accounts for most of the cases of drug non-approval, withdrawal and abandonment of drugs.^30,31^ In current clinical trials, drug hepatotoxicity still is a major problem because patient-specific effects are unclear and DILI may occur after long latency periods. The heterogeneity of responses suggests that some event during the course of therapy renders the patients particularly sensitive and this could be inflammatory episodes. The histological evaluation of liver biopsies revealed elevated inflammatory scores in samples from DILI patients^32^ and serum proteomic profiling of patients with DILI showed an increase of components associated with inflammation.^33^ In numerous animal studies evidence was provided that even a mild inflammation can decrease the threshold for hepatotoxicity and thereby enhance the sensitivity to chemically induced damage in the liver.^34–36^ Interestingly, it was suggested that the major pro-inflammatory cytokine TNFα is capable of enhancing liver damage in rodents caused by different xenobiotics.^37–39^ An interaction between inflammation and DCF in inducing hepatotoxicity was observed in rats,^40^ suggesting that inflammatory stress is a susceptibility factor for DCF-mediated toxicity. Furthermore, AMD treatment of rats during inflammation led to liver injury and increased TNFα serum concentrations contributing to hepatotoxicity.^41^ For APAP it was observed that TNFα is released in response to APAP intoxication and it was proposed that TNFα is responsible for certain pathological manifestations of APAP-induced hepatotoxicity.^42^ Whereas for these DILI compounds some information is available regarding a potential impact of inflammatory responses on hepatoxicity, for XIM-induced hepatotoxicity an immune-related mechanism was suggested, and FIAU shows a human-specific mitochondrial toxicity,^2^ and hence the impact of inflammation is less clear. Our model-based approach provides the means to systematically evaluate the impact of DILI compounds on TNFα-induced responses and resolve similarities as well as distinctions.

Current approaches are limited in the ability to cover the variability of patients and there is an urgent need for improved test systems. Mathematical modeling offers a powerful approach to tackle these challenges, because several factors affecting the outcome of drug exposure to hepatocytes can be investigated, the effects can be quantified and unobserved states can be predicted. Thereby, regulatory mechanisms affected by DILI compounds can be elucidated to improve possibilities to predict DILI.

One of the top ten DILI-causing drugs is DCF. It was shown that TNFα enhances the cytotoxicity of DCF^4^ and that DCF interferes with TNFα-induced NFκB signal transduction^4^ and causes several stress responses.^5,43^ TNFα is a pleiotropic cytokine that promotes proliferative but also cytotoxic responses,^7,9,44–47^ and therefore interference with the tightly regulated balance of responses due to drug exposure could shift the threshold toward hepatotoxicity. However, the underlying mechanisms of how compounds such as DCF affect TNFα-induced NFκB signal transduction in particular during early time points was previously not resolved. We developed a dynamic pathway model of TNFα-induced NFκB signal transduction that enabled us to quantitatively examine the perturbations caused by compounds and to analyze the processes at the beginning of a cellular decision, which appear to be crucial for cell survival under drug exposure.

Compared to previous mathematical models of NFκB signal transduction,^11–14,17,48^ we extended the dynamic pathway model of TNFα-induced NFκB signal transduction by several aspects. First, we introduced all four states of IKK upon TNFα stimulation into our mathematical model. In the study conducted by Behar and colleagues, the authors suggested a particularly important role of IKK in modulating NFκB signal transduction. This proposition is supported by our mathematical model, which identified reduced IKK activation as a major effect of DCF. Further, our analysis suggested that DCF enhanced the inactivation of phosphorylated IKK (pIKK) due to phosphorylation at the C terminus (ppIKK). Both effects lead to reduced levels of pIKK, and consequently delayed NFκB signal transduction. Second, we applied four states of the NFκB:IκBα complex. Based on NFκB and IκBα phosphorylation data in combination with Co-IP experiments, we were able to distinguish these four states.

The resulting identifiable mathematical model was capable of describing the experimental data generated for HepG2 cells and for PHHs and identified by employing L_1_ regularization two major interaction points of DCF in the NFκB signal transduction pathway. We showed that both regulatory levels, the upstream kinase IKK and the free, cytoplasmic IκBα, define the hepatotoxic outcome of compound treatments. To confirm the broad applicability of the established dynamic pathway model, we translated the regulatory mechanisms found for DCF to test scenarios with four other DILI-causing drugs with diverse putative mechanisms.^2^ Although their different mechanisms might lead to diverse rate modifications within the NFκB pathway, it is likely that the key regulators identified for DCF would be affected by the other DILI compounds as well. Model calibration based on the TNFα-induced dynamics of the NFκB pathway upon co-treatment with amiodarone, paracetamol, ximelagatran, and fialuridine allowed us to quantify the drug-induced effect strengths relative to DCF and predict the dynamics of nuclear translocation of NFκB upon co-treatment with these compounds.

Amiodarone is an antiarrhythmic lipophilic compound that was described to accumulate in the liver,^49^ a scenario that is often excluded in pharmacokinetic studies.^50^ It was observed that amiodarone induces severe hepatotoxicity in rats and TNFα contributes to a decrease of the drug toxicity threshold.^41^ Furthermore, TNFα co-treatment in Hepa1c1c7 cells revealed that TNFα potentiated the amiodarone-induced cytotoxicity.^51^ Concordant with these studies, our studies showed an early effect of amiodarone on TNFα-induced NFκB signal transduction and therefore highlight the importance of the integration of inflammatory responses in amiodarone-induced liver injury. Interestingly, co-treatment of amiodarone with TNFα caused similar death (caspases) and stress responses as co-treatment with DCF,^4,5,51^ which is reflected by our observed similar effects of DCF and amiodarone on the TNFα-induced dynamics of pIKK, pIκBα, and pNFκB (Fig. 6). However, the rather small differences on the dynamics of IκBα indicated potential differences in the modes of subsequent cell death, as seen in comparison to paracetamol, which does not induce the activation of caspases but rather necroptosis.^52,53^ In contrast to DCF and amiodarone, paracetamol is affecting neither the phosphorylation of IKK nor IκBα, but is decreasing the levels of IκBα and the phosphorylation of NFκB (Fig. 6). The impact of paracetamol could thereby be described by our dynamic pathway model. These observations point to a slightly different mechanism that includes the activation of other pathways, e.g., the JNK by paracetamol.^54–56^ Even though the hepatotoxicity caused by paracetamol is rather complex and other mechanisms might also play a role,^2^ we could detect and integrate the effects caused by paracetamol with our dynamic pathway model.

As seen in the case of fialuridine our model still has limitations. It is proposed that this compound has mitotoxic effects^57^; however, previous *in vitro* assays failed to detect fialuridine as hepatotoxic compound and underlying mechanisms apparently are very complex. Only recently, a 3D PHH spheroid model system was described which facilitated the detection of fialuridine toxicity *in vitro*,^58^ thus providing a starting point for further studies, which can be combined with our approach and used to further improve the established dynamic pathway model.

Ximelagatran showed no effects on TNFα-induced NFκB signal transduction in our studies, which is in line with previous reports suggesting an immune-mediated mechanism. Extensive studies were performed to unravel mechanisms contributing to the hepatotoxicity of ximelagatran; however, no underlying mechanism could be defined. The metabolism of ximelagatran does not involve the CYP450 system and reactive metabolites are not formed.^2^ Neither did hepatotoxicity by ximelagatran show a dose dependency.^59^ Due to a distinct geographic distribution of liver injury after ximelagatran treatment, the involvement of genetic factors was suggested.^59^ Recently, it was reported based on *in silico* and *in vitro* studies that ximelagatran directly interacts with a human leukocyte antigen strengthening the hypothesis of an immune response mechanism.^60^

DCF is known to generate protein adducts in hepatocytes.^61^ In addition, previous studies showed that DCF interferes with TNFα-induced NFκB signal transduction.^4,5^ Because membrane receptors are the starting point of various signal transduction pathways, we quantified the impact of TNFα and of the co-treatment with DCF on the number of TNFR1 receptor clusters per µm² and their size at the plasma membrane. A previous study indicated that subtle changes in TNFα–TNFR1 interactions might lead to higher-order clustering.^62^ We showed by SMLM that co-treatment with TNFα and DCF did neither significantly change the number of TNFR1 clusters per µm² both for stimulated or for non-stimulated cells nor did it change the cluster size. In line with these results our model-based studies revealed that reduced activation of the TNFR1 due to interference of DCF is not sufficient to explain the impact we detected on the dynamics of TNFα-induced NFκB signal transduction.

Taken together, the developed mathematical model provides a unique tool to quantitatively assess already in the preclinical phase the impact of compounds on a major aspect of inflammatory signaling such as TNFα-induced NFκB signal transduction. Our approach enables us to predict the interaction points of several compounds within the TNFα-induced NFκB signaling pathway even though the tested compounds have different putative toxicity mechanisms.^2^ Apparently, even if the induced death pathways differ between the tested compounds, for example, mainly via apoptosis for DCF^4,63,64^ or via necroptosis as for paracetamol,^52,53^ already rather early effects on TNFα-induced NFκB signal transduction are detectable. Based on these quantitative data, our dynamic pathway model is capable to quantify the compound-induced impact on nuclear NFκB and thereby predict how these compounds interfere with NFκB-induced transcription.

## Methods

### Reagents and antibodies

Recombinant human TNFα (210-TA) was acquired from R&D Systems and was reconstituted in sterile PBS containing 0.3% BSA. Diclofenac sodium (DCF, D6899), amiodarone hydrochloride (AMD, A8423), paracetamol (APAP, A7085), and fialuridine (FIAU, SML0632) were obtained from Sigma. Ximelagatran (XIM) was obtained from AstraZeneca. All compounds were dissolved in DMSO (Sigma). The antibodies against pIKKα/β (#2697), IKKβ (#2370), IKKα (#2682), pNFκB (#3031), NFκB (#6956), pIκBα (#2859), and IκBα (#9242) were from purchased from Cell Signaling Technologies. Secondary horseradish peroxidase-coupled antibodies were obtained from Dianova.

### Cell lines, cell culture, and cell stimulation

Human hepatoma HepG2 cells (HB8065) were obtained from American Type Culture Collection, cultured in Dulbecco’s Modified Eagle Medium (DMEM) without phenol red (Gibco 31053-044) supplemented with 10% (v/v) fetal bovine serum, 1% (v/v) Penicillin/Streptomycin (Gibco), 2 mM L-Glutamine (Gibco), and 1 mM Sodium Pyruvate (Gibco) and used for experiments between passage 5 and 20. The HepG2 cell line was authenticated using Multiplex Cell Authentication and the purity of cell line was validated using the Multiplex Cell Contamination Test by Multiplexion (Heidelberg, Germany) as described recently.^65,66^ HepG2 cells were serum-starved over night with DMEM supplemented with 1 mg/ml BSA, 1% (v/v) Penicillin/Streptomycin (Gibco), and 2 mM L-Glutamine (Gibco). DCF was dissolved in DMSO as 200-fold stock and prepared freshly for every experiment. Cells were incubated for 30 min with DCF (final concentration 500 µM) or the respective compound (final concentration AMD 35 µM; APAP 10 mM; XIM 800 µM; FIAU 500 mM) before treatment with TNFα (final concentration 10 ng/ml).

### Primary human hepatocytes

Fresh PHHs were isolated as previously described^29^ from three different donors (104, 105 and, 107 from UoL) with the following characteristics:

**Table:** 

ID	General information							
	Sex	Age	Weight (kg)	BMI	Ethnicity	BP	Alcohol units/week	Cigarettes/day
104	M	85	80.7	30	Caucasian	178/75		Ex smoker 20 years
105	F	45	70.9	25.4	Caucasian	132/87	10	Ex smoker
107	M	60	81.6	26.3	Caucasian	104/64		Ex smoker

ID	Patient health	
	Diagnosis	Disease
104	×3 CRLM (right hepatectomy ×1 VIII, ×2 VI)	Anterior resection (BowelCa T4bN2M0), AF, hypertension, DMT2, osteoarthritis
105	Bismuth 3A + caudate lobe cholangioCA (radical bile duct excision + en bloc right hemihepatectomy + caudate lobectomy + partial IVC resection)	Hysterectomy
107	×3 CRLM (VI resected, III metastasectomy, IV ablated)	Defun ileosotomy, AP resection (rectalCa T4N2M0 R1), relapse lung + liver, lung ablation, liver ablation, lung + liver ablation

ID	Medication excluding chemotherapy		
	Drug	Dose	Frequency
104	NKDA, metformin, bisoprolol, perindopril	500 mg/1.25 mg**/**4 mg	Three daily/once daily/once daily
105	Morphine sensitivity, omeprazole, vitamin B12, paracetamol, piriton, HRT		
107	NKDA, loperamide		PRN

ID	Chemotherapy
	Drug
104	5FU
105	None
107	Chemoradio cetuximab, irinotecan/FOLFOX**/**FOLFOX

Liver resections were received as surgical waste from Aintree Hospital, Liverpool, UK, with full patient consent and ethical approval from the National Research Ethics Service (REC reference: 11/NW/0327). After isolation, cells were seeded onto collagen I coated 6-well plates and after an adhesion time of 3 h medium and cells were left for overnight incubation. Cells were then washed three times the next day and medium was replaced by growth factor depletion medium (Williams Medium E (Biochrome), 2 mM L-Glutamine (Gibco), and 1% (v/v) Penicillin/Streptomycin (Gibco)) for 4 h prior to the experiment.

### Time-resolved experiments and cell lysis

For time course experiments, HepG2 cells or PHHs were stimulated with recombinant human TNFα after DCF pre-treatment of 30 min for the indicated times. Cells were then lysed using a cell lysis buffer (150 mM NaCl; 20 mM Tris-HCl pH 7.4; 10 mM NaF; 1 mM EDTA pH 8; 1 mM ZnCl_2_ pH 4.0; 1 mM MgCl_2_; 1 mM Na_3_VO_4_; 10% glycerol; 1% (v/v) NP-40; 0.1% Aprotinin; 0.1% 4-(2-Aminoethyl) benzenesulfonyl-fluoride hydrochloride) and lysates were rotated for 20 min at 4 °C, the total protein concentration of the supernatant was measured (bicinchoninic acid assay, Pierce) and aliquots of 30 µg were made for quantitative immunoblotting.

### Immunoprecipitation

For immunoprecipitation with IκBα antibody (Cell Signaling #9242) and NFκB antibody (Cell Signaling #6956), 500 µg of the protein lysate was used. Protein A-Sepharose (GE Healthcare) and indicated antibodies were added to the cell lysates and immunoprecipitation was performed overnight at 4 °C.

### SDS-PAGE, quantitative immunoblotting, and data processing

Total cell lysate aliquots or immunoprecipitations were loaded in a randomized order on 10% SDS-polyacrylamide gel electrophoresis gel (SDS-PAGE), separated by electrophoresis and transferred onto a polyvinylidene difluoride membrane (Millipore). Primary antibodies were incubated overnight at 4 °C and secondary antibodies for 1 h at room temperature. Chemiluminescence detection was performed using enhanced chemiluminescence substrate (GE Healthcare) and charge-coupled device camera-based signal detection was performed using an ImageQuant LAS 4000 system (GE Healthcare). Quantification was performed using ImageQuant TL (GE Healthcare). Appropriate lanes were manually selected and the width of all lanes was fixed. Protein bands of interest were manually selected and the height of the quantification square was adjusted to optimize the coverage of the selected protein band and reduce the amount of background included. Compensations for band distortions were added as necessary. The background was subtracted with the rolling ball method.

### Live-cell imaging

HepG2 RelA-GFP (NF-kB, p65) and A20-GFP BAC reporter cell lines were generated and characterized as described previously.^67^ Accumulation of GFP levels or nuclear translocation, and Hoechst staining was monitored using a Nikon TiE2000 confocal laser microscope (lasers: 640, 540, 488, and 408 nm). This microscope is equipped with an automated stage and perfect focus system at 37 °C with humidified atmosphere and 5% CO_2_/air mixture. During starvation overnight, HepG2 cells were stained with 50 ng/ml Hoechst_33342_ to visualize the nuclei. The Hoechst medium was replaced with exposure medium containing DCF. After 30 min pre-exposure to compound only, the medium was spiked with TNFα (1:20 dilution in starvation medium) up to a final concentration of 10 ng/ml TNFα. To prevent a delay in TNFα response in the oscillations of the RelA-GFP reporter, TNFα was added at the microscope per well, directly upon imaging of the first image (*t* = 0). Quantitative image analysis was performed with CellProfiler version 2.1.1^68^ with an in house developed module implementing the watershed masked algorithm for improved nuclear segmentation.^69^ Image analysis results were stored as HDF5 files, png images with the segmentation results were stored for quality control. Data analysis and further quality control was performed using the in house developed R package H5CellProfiler. Quantitative data of three independent experiments were used for model input.

### Single-molecule localization microscopy and data analysis

For single-molecule microscopy experiments, cells were seeded into 8-well chamber slides (Sarstedt) and grown until confluency was achieved. FCS-free medium containing 0.1% BSA was applied at least 12 h prior to experiments to the cells for starvation. For DCF treatment, cells were either treated with 500 µM DCF in DMSO (final concentration of DMSO: 0.5% (v/v)) or with DMSO (0.5% (v/v) in culture media) as control. For TNFα (ImmunoTools) treatment, cells were either treated with 10 ng/ml in PBS supplemented with 0.3% BSA (final concentration of BSA 3 × 10^−4^% (w/v)) or with BSA only as a control condition (final concentration of BSA 3 × 10^−4^%). After incubation with DCF for 30 min, TNFα was added to the medium and co-incubated for 0, 2, 5, or 10 min. After incubation, cells were fixed and prepared for immunostaining.

For fixation, medium was removed and cells were washed with pre-warmed 400 mM sucrose (37 °C) (Merck). Cells were incubated in fixation buffer (4% formaldehyde (methanol free; Thermo Scientific), 0.1% glutaraldehyde (Sigma), and 400 mM sucrose) for 15 min. Samples were extensively washed (at least three times) in PBS, followed by blocking in 2% BSA for 30 min. Samples were incubated with a primary antibody solution (monoclonal mouse anti-hTNFR1 (Abcam, clone: H398), 3 µg/ml in 2% BSA) for 1 h at room temperature. Primary antibody solution was removed by at least three washing steps in PBS. Cells were incubated in secondary antibody solution (F(ab′)2 fragment-goat anti-mouse labeled with AlexaFluor647 (A-21237, Life Technologies), 2 µg/ml in 2% BSA) for 1 h at room temperature, followed by three washing steps in PBS. After fixation with 4% formaldehyde, cells were washed at least three times with PBS. For negative control measurements, cells were incubated with secondary antibody only.

SMLM experiments were performed with a custom-built setup as described earlier.^70^ Briefly, an inverted microscope (Olympus IX71, Olympus) equipped with a nose piece (Olympus) was used. A read out laser emitting at 643 nm (diode laser, iBEAM smart, Toptica) was coupled into an acousto-optic tunable filter (AAOptics) for illumination intensity adjustment. A second laser emitting at 405 nm (CUBE 405-50C, Coherent) was combined with the 643 nm laser with an appropriate dichroic mirror. Widefield illumination was achieved by focusing the laser beams onto the back-focal plane of a 100× oil immersion objective (PLAPO 100× TIRFM, NA ≥ 1.45, Olympus). A translatable mirror was used for switching between widefield and total internal reflection illumination. Fluorescence emission was detected by the same objective, and fluorescence light was filtered with a bandpass filter (ET 700/75, AHF) and imaged with an EMCCD camera (DU-897U-CSO-#BV, iXon Ultra, Andor). The magnification optics led to an effective image pixel size of 157 nm.

SMLM imaging was performed following the dSTORM protocol.^71^ An imaging buffer (10% w/v glucose (Sigma), 40 mg/ml catalase (Sigma), 0.5 mg/ml glucose oxidase (Sigma), and 100 mM β-mercaptoethylamine (MEA; Sigma) in PBS, pH 7.8–8) was added to the sample. Laser intensities of 2–2.5 kW/cm² (643 nm) were used for read out of the fluorescence signal. Appropriate switching rates were achieved by UV illumination ranging between 0–10 W/cm². An image series of 20,000 frames per cell were recorded with an integration time of 30 ms per frame.

Single-molecule data were analyzed with rapidSTORM.^72^ Super-resolved images were generated at a pixel size of 10 nm and further analyzed using the LAMA package^73^ which can be obtained free of charge (http://share.smb.uni-frankfurt.de/).

The localization precision was calculated using a nearest neighbor analysis.^74^ The number of TNFR1 clusters was determined by an image-based analysis routine as described earlier.^27^ Briefly, a region of interest (ROI) was chosen for each cell and the number of TNFR1 clusters per µm² was determined using the Fiji Plugin “Analyze Particles”.^75^ The size of individual clusters was determined for five ROI per cell, each with a size of 2 × 2 µm² using Density-Based Spatial Clustering of Applications with Noise (DBSCAN)^28^ with input parameters *N*_min_ = 10 and *ε* = 30 nm. Subsequently, the radius of coextensive clusters with circular shape was calculated. For each condition, at least 13 cells were analyzed from at least 3 different independent experiments. Statistical hypotheses were tested using Kolmogorov–Smirnov test implemented in OriginPro 9.1G (OriginLab). This test allows probing similarity of small, non-normal distributed populations (significance level *α* = 0.05).

### Mathematical modeling

The TNF⍺/NFκB signaling pathway is modeled by ODEs. Interactions between species are assumed to follow mass-action kinetics. The model topology and details about the model equations are described in Supplementary Material 1. All modeling steps, from model setup to identifiability analysis, parameter estimation, and uncertainty analysis were carried out using the R package dMod available on the Comprehensive R Archive Network.

The dynamic model is described by ODEs $\dot x$ = *f*(*x*, *p*_dyn_) where *x* denotes the vector of dynamic states such as IKK, NFκB, IκB⍺, etc., and *p*_dyn_ denotes the vector of model parameters such as activation rates, dissociation rates, etc. Initial values *x*_0_ = *x*(*t* = 0) for all dynamic states are treated like model parameters. The dynamic states are linked to experimental observations via an observation function *y* = *g*(*x*, *p*_obs_). This function accounts for the fact that (i) only certain combinations of dynamic states can be experimentally observed, that (ii) measurements from fluorescence microscopy suffer from photobleaching and that (iii) observations are evaluated on the log scale.

### Identifiability analysis

The structure of the differential equations and the observation function can induce symmetries, i.e., a functional relationship between the model, initial value, and observation parameters that guarantee the invariance of the predicted observation. The TNF⍺/NFκB model was systematically scanned for such relationships using a Lie-group approach.^76^ The number of free parameters necessary to describe the model was thereby reduced.

### Parameterization and steady states

The same model and observation structure is employed to describe different experimental conditions such as TNFα/no TNFα or DCF/no DCF. However, depending on the condition, parameters are set differently: model, initial value, and observation parameters are obtained by condition-specific functions, (*p*_dyn_, *x*_0_, *p*_obs_)_*i*_ = *ϕ*_*i*_(*θ*) from one single, overarching vector of parameters, *θ*. Different experimental conditions are denoted by *i*. The functions *ϕ*_*i*_ used for the TNF⍺/NFκB model account for (i) DCF/TNF-specific setting of parameters, (ii) a general log transform of all parameters, and (iii) a steady-state transformation where all initial values *x*_0_ are computed from the dynamic parameters *p*_dyn_ and total levels of IKK and NFκB based on the condition *f*(*x*_0_, *p*_dyn_) = 0.

### Data preprocessing

Experiments were performed in at least three biological replicates. Samples for quantitative immunoblot analysis from different replicates but equal experimental conditions were partially measured on the same gel, whereas others were measured on different gels. Replicates measured by fluorescence microscopy were analyzed on independent plates on different days. Accordingly, to account for a systematic deviance between equal conditions but different gels/plates, we used the scaling model $S_{i,k,n} = \frac{{y_{i,n}}}{{s_k}}$ with the measurements *S*_*i*,*k*,*n*_ for different conditions *i*, gels/plates *k*, and timepoints *t*_*n*_. The symbols *y*_*i*,*n*_ indicate time course parameters and *s*_*k*_ denotes scaling parameters. The time course parameters were obtained by maximum-likelihood estimation. They represent the average dynamics over all biological replicates. As such they condense the original replicate data and replace it for dynamic modeling.

### Parameter estimation by the maximum-likelihood method

The model response, i.e., the dynamics of the pathway species and the corresponding observation are parameterized by the parameters *θ*. These parameters are estimated by the maximum-likelihood method. Our likelihood is based on the normal distribution, i.e., differences between the model’s prediction for the observation at timepoints *t*_*n*_ and the actual observations *y*_*i*,*n*_ are normally distributed: (*g* ∘ *x* ∘ *ϕ*_*i*_ (*t*_*n*_, *θ*) − *y*_*i*,*n*_) ~ *N*(0, *σ*^2^(*θ*)). Observations for different timepoints are assumed to be statistically independent. The expected variance *σ*^2^(*θ*) of the residuals is constant per observed target, but unknown, and is therefore expressed by additional parameters collected in *θ*. From these assumptions, the deviance, i.e., minus twice the log-likelihood is derived:

1 $$l\left( \theta \right) = \mathop {\sum }\limits_{i,n} \left( {\frac{{g \circ x \circ \phi _i\left( {t_n,\theta } \right) - y_{i,n}}}{{\sigma \left( \theta \right)}}} \right)^2 + {\mathrm {log}}\left[ {\sigma ^2\left( \theta \right)} \right].$$

The function *l*(*θ*) is minimized with respect to *θ* to obtain parameter estimates $\hat \theta$.

### Uncertainty analysis and model reduction

Parameter uncertainty was analyzed by the profile-likelihood method.^77^ The method is not only suited to determine parameter uncertainty in nonlinear models, it is also convenient to determine relationships between the model parameters and to propose possible model reductions.^78^ Based on the profile-likelihood approach (Fig. S4) and the experimental data for TNFα-induced NFκB signaling, the initial model was reduced to a core model (Fig. S5). Within this model, key parameters to be altered to describe the TNFα-induced response of DCF-treated cells were identified.

### L_1_ regularization

Identification of DCF-specific parameter alterations is based on the LASSO method.^26^ At first, every reaction rate was considered as a possible target of DCF. The altered rate k^*^ of any reaction rate *k* is related to its original value by the equation k^*^ = *k* · *r*_*k*_ or, equivalently, applying the logarithm, log(k^*^) = log(*k*) + Δ_*k*_, where Δ_*k*_ = log(*r*_*k*_). Introducing the L_1_ regularization function $\phi _\lambda \left( {{\mathrm{\Delta }}_k} \right) = \lambda \mathop {\sum }\limits_k \left| {\Delta _k} \right|$, the augmented objective function can be expressed as:

2 $$l_\lambda \left( {k,{\mathrm{\Delta }}_k} \right) = l\left( {k{\mathrm{|data}}_{{\mathrm{control}}}} \right) + l\left( {k \cdot e^{{\mathrm{\Delta }}_k}{\mathrm{|data}}_{{\mathrm{DCF}}}} \right) + \phi _\lambda \left( {{\mathrm{\Delta }}_k} \right).$$

Here, $l\left( {k{\mathrm{|data}}_{{\mathrm{control}}}} \right)$ and $l\left( {k \cdot e^{{\mathrm{\Delta }}_k}{\mathrm{|data}}_{{\mathrm{DCF}}}} \right)$ describe twice the negative log-likelihood of the model given the TNFα-stimulated control data and of the model with modified rates given the data of TNFα and DCF co-treated cells, respectively. First, the objective function *l*_*λ*_(*k*, Δ_*k*_) was minimized for *λ* = 0. In this setting, the difference parameters Δ_*k*_ could be freely estimated, allowing changes in all parameters in response to DCF treatment. Subsequently*, λ* was gradually increased. Larger values of *λ* increase the pressure to reduce the Δ-values, eventually leading to zero values. In this case, no parameter differences between the control and the DCF data is allowed.

### Data availability

The reactions of the full and the reduced model are shown in Tables S1 and S5, respectively, with the corresponding differential equations in Tables S2 and S6. The observation functions linking the states of the ODE model to the experimentally observed quantities are listed in Table S3. The estimated model parameters are shown in Tables S7–S9. The density of TFNFR1 clusters and corresponding cluster radii are shown in Table S10. Quantitative immunoblots for all the experimental data is shown in Figs. S8–S45. Data that have been used for mathematical modeling is provided as Data Set csv file.

### Code availability

The custom R packages blotIt2 and dMod used for data preprocessing with a scaling model and parameter estimation in ODE models, respectively, are available on github as open source R packages under the GPL license (https://github.com/dkaschek/blotIt2, https://github.com/dkaschek/dMod). The ODE model as being implemented in dMod is available on https://github.com/dkaschek/dMod_Examples. The concepts and usage of dMod have been introduced previously.^79^

## Electronic supplementary material

Supplementary material 1

Data used for model calibration as CSV file

## Electronic supplementary material

**Supplementary information** accompanies the paper on the *npj Systems Biology and Applications* website (10.1038/s41540-018-0058-z).
