# Supplementary material for: Model-based identification of TNFα-induced IKKβ-mediated and IκBα-mediated regulation of NFκB signal transduction as a tool to quantify the impact of drug-induced liver injury compounds
Source: NPJ Syst Biol Appl. 2018 Jun 11;4:23. doi: 10.1038/s41540-018-0058-z (PMC5995845; doi:10.1038/s41540-018-0058-z)
Supplement: Supplementary file 1 — Supplementary material 1 [file 41540_2018_58_MOESM1_ESM.pdf]

Supplementary Material 1

Angela Oppelt, Daniel Kaschek, Suzanna Huppelschoten, Rowena Sison-Young, Fang Zhang, Marie Buck-Wiese, Franziska Herrmann, Sebastian Malkusch, Carmen L. Krüger, Mara Meub, Benjamin Merkt, Lea Zimmermann, Amy Schofield, Robert P. Jones, Hassan Malik, Marcel Schilling, Mike Heilemann, Bob van de Water, Christopher E. Goldring, B. Kevin Park, Jens Timmer, Ursula Klingmüller

April 6, 2018

Contents

1 Fluorescence microscopy data **2**

2 Dynamic pathway modeling **4**
2.1 The full model . . . 4
2.1.1 Reactions and differential equations . . . 4
2.1.2 Observation function . . . 7
2.1.3 Steady-state assumptions . . . 7
2.1.4 Parameter transformations . . . 8
2.1.5 Parameter estimation . . . 8
2.2 Model reduction . . . 9
2.3 The reduced model . . . 10

3 Impact of DCF on the TNFa/TNFR interaction in HepG2 cells **17**

4 Predicted A20 dynamics for DILI compounds **17**

5 Data availability **18**

6 Dose response curves **19**

7 Quantitative immunoblot data **20**

1 Fluorescence microscopy data

Single-cell fluorescence microscopy data is combined with average-cell quantitative immunoblot data in a mathematical model based on ordinary differential equations to describe the average dynamics. In this context, it is important to check whether the average cell shows the same dynamics as the averaged dynamics over all cells. To this end, the distance of the NFκB dynamics of each cell from the average NFκB dynamics has been computed by the equation

$$D_k = \sqrt{\sum_i (x_{ik} - \langle x_i \rangle)^2}, \quad (1)$$

where k denotes different cells, $\langle x_i \rangle$ is the average NFκB signal at time point t_i and x_{ik} is the NFκB signal of the cell k at time point t_i. Figure S1 shows 62 randomly chosen cells, sorted by their distance to the average dynamics.

The plot shows that different cells are well synchronized over 360 minutes. The single-cell data is highly consistent with the average data corroborating our reduction approach of using the mean fluorescence signal for model calibration.

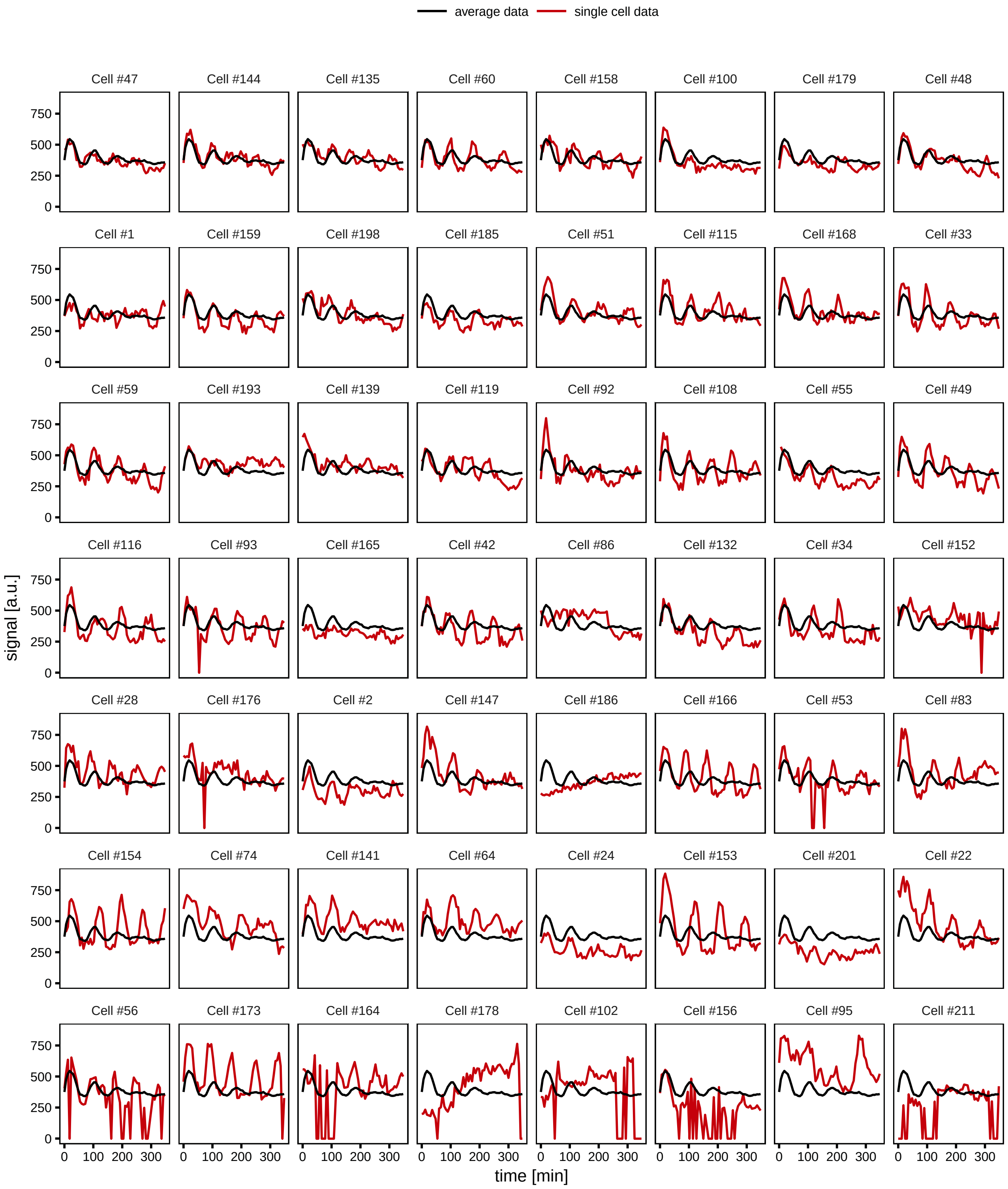

Figure S1: Single-cell versus average data. The plot shows nuclear signals of 62 randomly selected HepG2 cells expressing NFκB-GFP treated with TNFα (red curves). The cells are sorted by their similarity to the average fluorescence signal (black curve). The graphical analysis indicates that all cells share the same frequency of oscillation and consistently react to TNFα stimulation.

2 Dynamic pathway modeling

2.1 The full model

2.1.1 Reactions and differential equations

The model topology is represented by Figure S2.

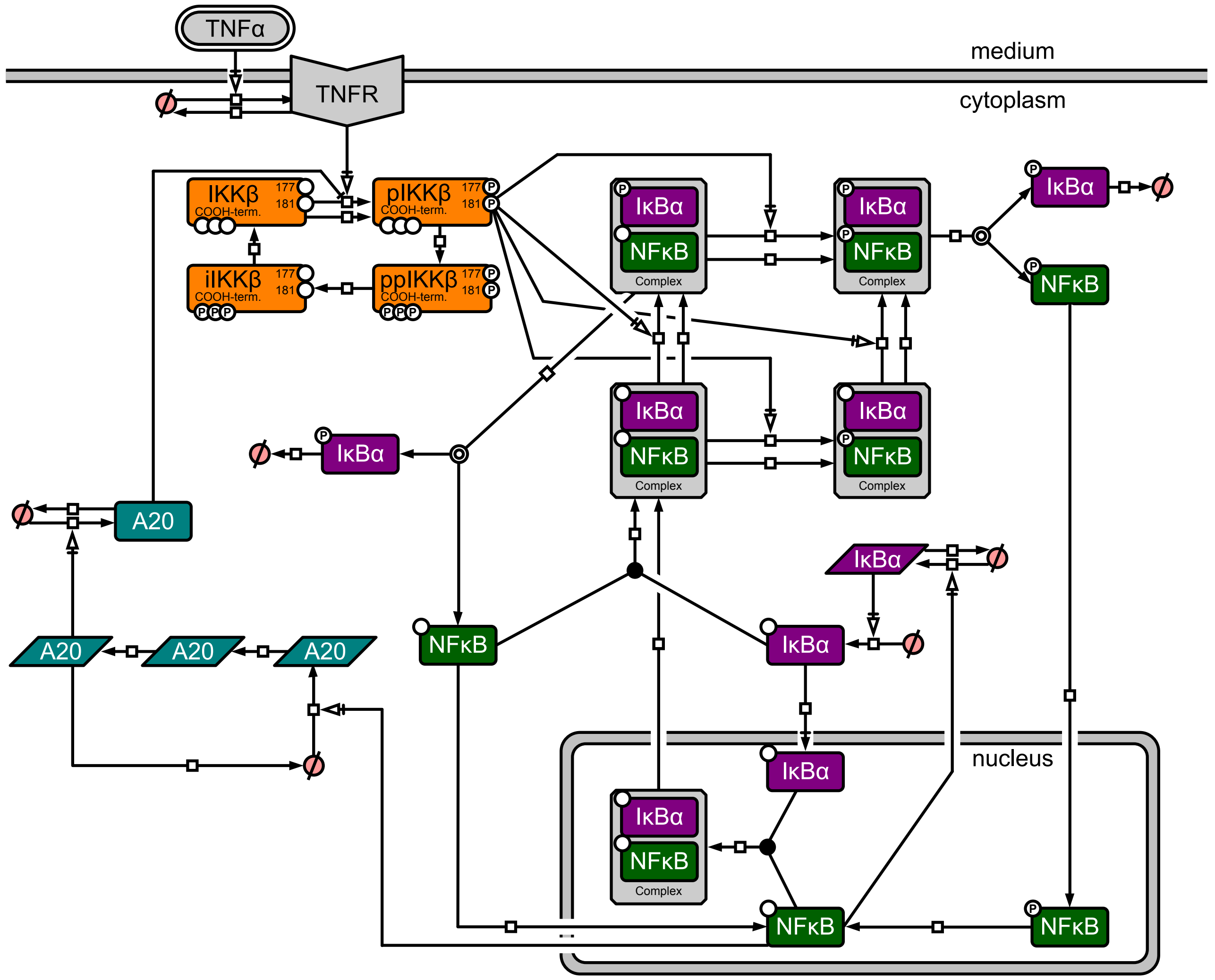

Figure S2: Reaction scheme of the full model. Schematic representation of the model indicating the considered components and reactions. Arrows indicate biochemical reactions. Boxes with round corners symbolize proteins, parallelograms represent RNA. Slashed circles represent degradation or production. Black lines indicate reactions induced by pathway activation.

Each arrow in the schematics corresponds to an elementary process in the mathematically formulated reaction network. Table S1 shows a list of all reactions included in the full model. Some of the reaction rates are formulated as products of a reference rate parameter and additional rate parameters, indicated by the prefix `eta_`, to systemmatically deviate from the reference rate.

	Educt	→	Product	Rate
1		→	TNFR	uptake · TNF
2	TNFR	→		deact_TNFR · TNFR
3	Ikk	→	pIkk	(act_Ikk_by_TNF / (1 + A20 / inh_Ikk)) · TNFR · Ikk
4	Ikk	→	pIkk	act_Ikk_Base · Ikk
5	pIkk	→	ppIkk	act_pIkk · pIkk
6	ppIkk	→	iIkk	deact_ppIkk · ppIkk
7	iIkk	→	Ikk	trigger_iIkk · iIkk
8	NfkIkb	→	NfkpIkb	(act_Ikb_Base) · NfkIkb
9	NfkIkb	→	NfkpIkb	(act_Ikb_by_Ikk) · pIkk · NfkIkb
10	NfkIkb	→	NfkpIkb	(act_Ikb_by_Ikk · eta_s_ppIkk) · ppIkk · NfkIkb
11	NfkIkb	→	pNfkIkb	(act_Nfk_Base) · NfkIkb
12	NfkIkb	→	pNfkIkb	(act_Nfk_by_Ikk) · pIkk · NfkIkb
13	NfkIkb	→	pNfkIkb	(act_Nfk_by_Ikk · eta_s_ppIkk) · ppIkk · NfkIkb
14	pNfkIkb	→	pNfkpIkb	(act_Ikb_Base · eta_act_Ikb_Base_c_pNfkIkb) · pNfkIkb
15	pNfkIkb	→	pNfkpIkb	(act_Ikb_by_Ikk · eta_act_Ikb_c_pNfkIkb) · pIkk · pNfkIkb
16	pNfkIkb	→	pNfkpIkb	(act_Ikb_by_Ikk · eta_s_ppIkk · eta_act_Ikb_c_pNfkIkb) · ppIkk · pNfkIkb
17	NfkpIkb	→	pNfkpIkb	(act_Nfk_Base · eta_act_Nfk_Base_c_NfkpIkb) · NfkpIkb
18	NfkpIkb	→	pNfkpIkb	(act_Nfk_by_Ikk · eta_act_Nfk_c_NfkpIkb) · pIkk · NfkpIkb
19	NfkpIkb	→	pNfkpIkb	(act_Nfk_by_Ikk · eta_s_ppIkk · eta_act_Nfk_c_NfkpIkb) · ppIkk · NfkpIkb
20	NfkpIkb	→	Nfk + pIkb	split_NfkpIkb · NfkpIkb
21	pNfkpIkb	→	pNfk + pIkb	(split_NfkIkb · eta_split_pNfkpIkb) · pNfkpIkb
22	Nfk + Ikb	→	NfkIkb	form_complex · Nfk · Ikb
23		→	mIkb	prod_mIkb_by_nNfk · nNfk
24	mIkb	→		degrad_mIkb · mIkb
25		→	Ikb	prod_Ikb · mIkb
26	pIkb	→		degrad_Ikb · pIkb
27	Ikb	→	nIkb	(int_Nfk · eta_int_Ikb) · Ikb
28	pNfk	→	pnNfk	(int_Nfk · eta_int_pNfk) · pNfk
29	Nfk	→	nNfk	(int_Nfk) · Nfk
30	pnNfk	→	nNfk	deact_pnNfk · pnNfk
31	nIkb + nNfk	→	nNfkIkb	(form_complex · eta_form_compex_nuc) · nNfk · nIkb
32	nNfkIkb	→	NfkIkb	ext_nNfkIkb · nNfkIkb
33		→	RnaA20_1	build_RnaA20 · nNfk
34	RnaA20_1	→	RnaA20_2	shuttle_RnaA20 · RnaA20_1
35	RnaA20_2	→	RnaA20	shuttle_RnaA20 · RnaA20_2
36	RnaA20	→		degrad_RnaA20 · RnaA20
37		→	A20	build_A20 · RnaA20
38	A20	→		degrad_A20 · A20

Table S1: Table of reactions of the full model.

The table of reactions with the specified rate expressions uniquely determines the set of differential equations describing the dynamics of the system, see Table S2.

$\frac{d}{dt}$TNFR	=	1 · (uptake · TNF) - 1 · (deact_TNFR · TNFR)
$\frac{d}{dt}$Ikk	=	- 1 · ((act_Ikk_by_TNF / (1 + A20 / inh_Ikk)) · TNFR · Ikk) - 1 · (act_Ikk_Base · Ikk) + 1 · (trigger_iIkk · iIkk)
$\frac{d}{dt}$pIkk	=	1 · ((act_Ikk_by_TNF / (1 + A20 / inh_Ikk)) · TNFR · Ikk) + 1 · (act_Ikk_Base · Ikk) - 1 · (act_pIkk · pIkk)
$\frac{d}{dt}$ppIkk	=	1 · (act_pIkk · pIkk) - 1 · (deact_ppIkk · ppIkk)
$\frac{d}{dt}$iIkk	=	1 · (deact_ppIkk · ppIkk) - 1 · (trigger_iIkk · iIkk)
$\frac{d}{dt}$NfkIkb	=	- 1 · ((act_Ikb_Base) · NfkIkb) - 1 · ((act_Ikb_by_Ikk) · pIkk · NfkIkb) - 1 · ((act_Ikb_by_Ikk · eta_s_ppIkk) · ppIkk · NfkIkb) - 1 · ((act_Nfk_Base) · NfkIkb) - 1 · ((act_Nfk_by_Ikk) · pIkk · NfkIkb) - 1 · ((act_Nfk_by_Ikk · eta_s_ppIkk) · ppIkk · NfkIkb) + 1 · (form_complex · Nfk · Ikb) + 1 · (ext_nNfkIkb · nNfkIkb) · (Vnuc / 1)
$\frac{d}{dt}$NfkpIkb	=	1 · ((act_Ikb_Base) · NfkIkb) + 1 · ((act_Ikb_by_Ikk) · pIkk · NfkIkb) + 1 · ((act_Ikb_by_Ikk · eta_s_ppIkk) · ppIkk · NfkIkb) - 1 · ((act_Nfk_Base · eta_act_Nfk_Base_c_NfkpIkb) · NfkpIkb) - 1 · ((act_Nfk_by_Ikk · eta_act_Nfk_c_NfkpIkb) · pIkk · NfkpIkb) - 1 · ((act_Nfk_by_Ikk · eta_s_ppIkk · eta_act_Nfk_c_NfkpIkb) · ppIkk · NfkpIkb) - 1 · (split_NfkpIkb · NfkpIkb)
$\frac{d}{dt}$pNfkIkb	=	1 · ((act_Nfk_Base) · NfkIkb) + 1 · ((act_Nfk_by_Ikk) · pIkk · NfkIkb) + 1 · ((act_Nfk_by_Ikk · eta_s_ppIkk) · ppIkk · NfkIkb) - 1 · ((act_Ikb_Base · eta_act_Ikb_Base_c_pNfkIkb) · pNfkIkb) - 1 · ((act_Ikb_by_Ikk · eta_act_Ikb_c_pNfkIkb) · pIkk · pNfkIkb) - 1 · ((act_Ikb_by_Ikk · eta_s_ppIkk · eta_act_Ikb_c_pNfkIkb) · ppIkk · pNfkIkb)
$\frac{d}{dt}$pNfkpIkb	=	1 · ((act_Ikb_Base · eta_act_Ikb_Base_c_pNfkIkb) · pNfkIkb) + 1 · ((act_Ikb_by_Ikk · eta_act_Ikb_c_pNfkIkb) · pIkk · pNfkIkb) + 1 · ((act_Ikb_by_Ikk · eta_s_ppIkk · eta_act_Ikb_c_pNfkIkb) · ppIkk · pNfkIkb) + 1 · ((act_Nfk_Base · eta_act_Nfk_Base_c_NfkpIkb) · NfkpIkb) + 1 · ((act_Nfk_by_Ikk · eta_act_Nfk_c_NfkpIkb) · pIkk · NfkpIkb) + 1 · ((act_Nfk_by_Ikk · eta_s_ppIkk · eta_act_Nfk_c_NfkpIkb) · ppIkk · NfkpIkb) - 1 · ((split_NfkIkb · eta_split_pNfkpIkb) · pNfkpIkb)
$\frac{d}{dt}$pNfk	=	1 · ((split_NfkIkb · eta_split_pNfkpIkb) · pNfkpIkb) - 1 · ((int_Nfk · eta_int_pNfk) · pNfk)
$\frac{d}{dt}$Nfk	=	1 · (split_NfkpIkb · NfkpIkb) - 1 · (form_complex · Nfk · Ikb) - 1 · ((int_Nfk) · Nfk)
$\frac{d}{dt}$pIkb	=	1 · (split_NfkpIkb · NfkpIkb) + 1 · ((split_NfkIkb · eta_split_pNfkpIkb) · pNfkpIkb) - 1 · (degrad_Ikb · pIkb)
$\frac{d}{dt}$Ikb	=	- 1 · (form_complex · Nfk · Ikb) + 1 · (prod_Ikb · mIkb) - 1 · ((int_Nfk · eta_int_Ikb) · Ikb)
$\frac{d}{dt}$mIkb	=	1 · (prod_mIkb_by_nNfk · nNfk) - 1 · (degrad_mIkb · mIkb)
$\frac{d}{dt}$nIkb	=	1 · ((int_Nfk · eta_int_Ikb) · Ikb) · (1 / Vnuc) - 1 · ((form_complex · eta_form_compex_nuc) · nNfk · nIkb)
$\frac{d}{dt}$pnNfk	=	1 · ((int_Nfk · eta_int_pNfk) · pNfk) · (1 / Vnuc) - 1 · (deact_pnNfk · pnNfk)
$\frac{d}{dt}$nNfk	=	1 · ((int_Nfk) · Nfk) · (1 / Vnuc) + 1 · (deact_pnNfk · pnNfk) - 1 · ((form_complex · eta_form_compex_nuc) · nNfk · nIkb)
$\frac{d}{dt}$nNfkIkb	=	1 · ((form_complex · eta_form_compex_nuc) · nNfk · nIkb) - 1 · (ext_nNfkIkb · nNfkIkb)
$\frac{d}{dt}$RnaA20_1	=	1 · (build_RnaA20 · nNfk) - 1 · (shuttle_RnaA20 · RnaA20_1)
$\frac{d}{dt}$RnaA20_2	=	1 · (shuttle_RnaA20 · RnaA20_1) - 1 · (shuttle_RnaA20 · RnaA20_2)
$\frac{d}{dt}$RnaA20	=	1 · (shuttle_RnaA20 · RnaA20_2) - 1 · (degrad_RnaA20 · RnaA20)
$\frac{d}{dt}$A20	=	1 · (build_A20 · RnaA20) - 1 · (degrad_A20 · A20)

Table S2: List of differential equations of the full model

2.1.2 Observation function

The states of the ODE model, also called the internal states, are related to experimentally observed quantities, the observables, via an observation function. The observation function employed for our model is summarized in Table S3.

observable		relation with internal states
nuclear NFkB	=	log((s_Nuclei · (pnNfk + nNfk + nNfkIkb) + off_Nuclei) · exp(- tau_Nuclei · time))
cytoplasmic NFkB	=	log((s_Cytoplasm · (NfkIkb + NfkpIkb + pNfkIkb + pNfkpIkb + pNfk + Nfk) + off_Cytoplasm) · exp(- tau_Cytoplasm · time))
A20	=	log((s_A20 · A20 + off_A20) · exp(- tau_A20 · time))
IkBa	=	log(s_IkBa · (NfkIkb + pNfkIkb + NfkpIkb + pIkb + Ikb) + off_IkBa)
pNFkB	=	log(s_pp65 · (pNfkpIkb + pNfkIkb + pNfk) + off_pp65)
pIkBa	=	log(s_pIkBa · (NfkpIkb + pNfkpIkb + pIkb) + off_pIkBa)
pIKK	=	log(s_pIKK · (pIkk + ppIkk) + off_pIKK)
IP:IkBa | IB:IkBa	=	log(s_IkBa_IP · (NfkIkb + pNfkIkb + NfkpIkb + pIkb + Ikb) + off_IkBa_IP)
IP:NFkB | IB:NFkB	=	log(s_p65_IP · (NfkIkb + pNfkIkb + NfkpIkb + pNfkpIkb + pNfk + Nfk) + off_p65_IP)
IP:NFkB | IB:pIkBa	=	log(s_pIkBalpha_tp65_IP · (NfkpIkb + pNfkpIkb) + off_pIkBalpha_tp65_IP)
IP:NFkB | IB:pNFkB	=	log(s_pp65_IP · (pNfkpIkb + pNfkIkb + pNfk) + off_pp65_IP)
IP:IkBa | IB:pNFkB	=	log(s_tIkBalpha_pp65_IP · (pNfkpIkb + pNfkIkb) + off_tIkBalpha_pp65_IP)
IP:IkBa | IB:NFkB and IP:NFkB | IB:IkBa	=	log(s_tIkBalpha_tp65_IP · (NfkIkb + NfkpIkb + pNfkIkb + pNfkpIkb) + off_tIkBalpha_tp65_IP)
NFkB/IkBa ratio	=	log((NfkIkb + NfkpIkb + pNfkIkb + pNfkpIkb + pNfk + Nfk) / (NfkIkb + NfkpIkb + pNfkIkb + pNfkpIkb + pIkb + Ikb))

Table S3: List of observables

The observation functions collect all internal states contributing to the measurement by antibody- or fluorescent labeling-based methods. In addition, the equations contain scaling parameters (prefix `s_`) and offset parameters (prefix `off_`). The variance of the measurement data is most uniformly described on log-scale. Therefore, the log-transformation is applied to all observables. Fluorescence measurements suffer from photobleaching leading to an exponentally decaying signal which is accounted for by exponential functions within the observation function.

2.1.3 Steady-state assumptions

The system is assumed to be in a steady state at $t = 0$ min. Since the system has two conserved quantities, see Table S4, the dynamic parameters are not sufficient to determine the steady-state concentrations uniquely. Instead, additional quantities `total_NFkB` (c_1) and `total_Ikk` (c_2) being additional parameters, need to be provided to determine the steady-state together with the rate parameters p.

observable		relation with internal states
total_NFKB	=	1 · NfkIkb + 1 · NfkpIkb + 1 · pNfkIkb + 1 · pNfkpIkb + 1 · pNfk + 1 · Nfk + Vnuc · (1 · pnNfk + 1 · nNfk + 1 · nNfkIkb)
total_IKK	=	Ikk + pIkk + ppIkk + iIkk

Table S4: Conserved quantities

Let $C(x)$ be the conserved quantity expressions, i.e., $C_1(x)$ and $C_2(x)$ correspond to total NFκB and total IKK, respectively. The system is in a steady state when the model states do not change: $\dot{x} = f(x, p) = 0$. Steady-state values are determined solving the implicit equations

$$\begin{aligned} f(x(p), p) &= 0 \\ C(x(p)) - c &= 0 \end{aligned} \tag{2}$$

jointly. Given (p, c), the solution x^* of equation (2) is determined by Newton's method. The mapping $\phi_{\mathrm{SS}} : p \mapsto (p, x^*)$ is treated as a usual parameter transformation, see section 2.1.4. Here, c has been absorbed into the parameter vector p.

2.1.4 Parameter transformations

The solution of the dynamic system $\dot{x} = f(x, p)$ depends on dynamic parameters (rate constants, volumes, etc.) and initial value parameters x_0. The initial values are fully determined by the dynamic parameters and two additional parameters, `total_NFKB` and `total_IKK`. We chose to parameterize our system on a log-scale, meaning that any parameter p_i is expressed by its corresponding log-parameter θ_i via the relation $\theta_i = \log(p_i)$ or equivalently

$$p_i = \phi_i(\theta) = e^{\theta_i}. \tag{3}$$

In addition, parameters which are fixed to certain values are expressed by the parameter transformation ϕ, too, e.g., $p_i = \phi_i(\theta) = 1$ whenever i refers to a parameter fixed to 1. Finally, the steady-state transformation $\phi_{\mathrm{SS}} : p \mapsto (p, x^*)$ is concatenated with the transformation ϕ to yield the full transformation,

$$\phi_{\mathrm{SS}} \circ \phi : \theta \mapsto (p(\theta), x^*(p(\theta))), \tag{4}$$

translating a set of log-parameters into positive rate constants and positive initial values in steady-state.

2.1.5 Parameter estimation

Parameter estimation is based on the maximum-likelihood method. We assume that noise in the measurements are log-normally distributed. The objective function to be minimized to obtain parameter values is the negative log-likelihood

$$-2\log(L(\theta)) = \sum_i \left\| \frac{g \circ x \circ \phi_{\mathrm{SS}} \circ \phi(t_i, \theta) - y_i^D}{\sigma_i(\theta)} \right\|^2 - \log \sigma_i(\theta)^2 + \left\| \frac{\theta - \mu}{\sigma_\mu} \right\|^2. \tag{5}$$

Here, g denotes the observation function, x is the model prediction function, y_i^D refers to the data and $\sigma_i(\theta)$ is the measurement uncertainty. The first term of the sum donetes the least-squares contribution. Given the time point t_i and parameter vector θ, the model prediction $g \circ x \circ \phi_{\mathrm{SS}} \circ \phi$ is computed. The expected uncertainty $\sigma_i(\theta)$ of the ith residual is either taken from the data or computed from an error model (for observables Nuclei, Cytoplasm and A20). Here, we assume that each observable has a constant error on the log-scale which gives us one error model parameter per observable to be estimated. The second term reflects that the uncertainty is estimated. Small values of σ are penalized. The third term incorporates prior knowledge about the parameter position and range. Ideally, all necessary information to estimate the parameters is contained in the data. In practice, priors are used to regularize the estimation process. For the final result, the prior strength is lowered until its effect on the parameter estimates becomes negligible.

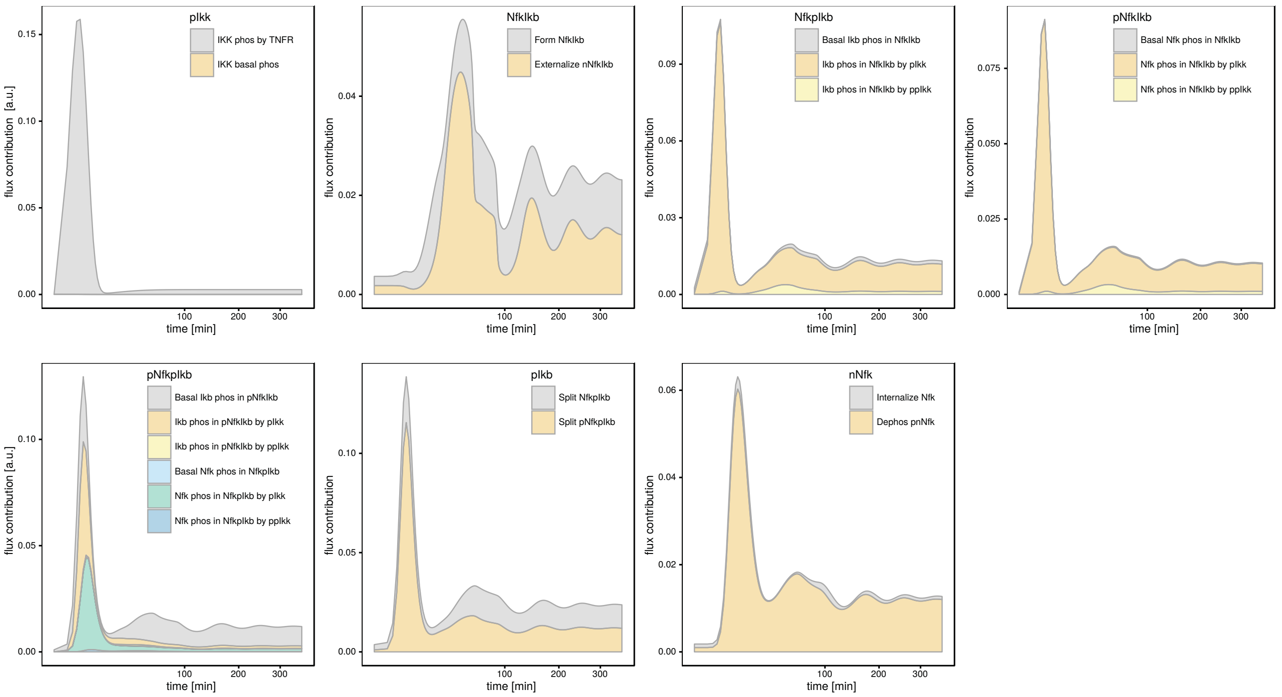

Figure S3: Reaction fluxes. The increase of concentration per time caused by several processes is shown for seven different targets, indicated in the figure legends. The different fluxes, distinguished by color, are accumulated to the total influx (topmost contour). Small fluxes appear as thin stripes.

2.2 Model reduction

The full model has been fitted to the experimental data. Based on the estimated parameters, the mathematical expressions as being collected in the Rate column of Table S1 were evaluated. Each expression is denoted as a reaction flux, describing the change of concentration per time caused by the corresponding reaction. Figure S3 shows all influxes for seven different internal states. Different reaction fluxes, shown in different colors, are stacked, i.e., the value of the flux is given by the height of the corresponding colored band. The accumulated height of all bands corresponds to the total influx.
The plot of pIkk fluxes indicates that basal IKK phosphorylation is negligible relative to IKK phosphorylation by TNFR. We can therefore drop the basal phosphorylation from the model without deteriorating the fit between model and data. Similarly, basal IkB phosphorylation in NfkIkb and basal Nfk phosphorylation in NfkIkb is so low that it is removed from the model. Ikb and Nfk phosphorylation by ppIkk is a negligible contribution in all targets and is therefore removed. Concerning the pNfkpIkb-complex, only basal Ikb phosphorylation of pNfkIkb, Ikb phosphorylation by pIkk and Nfk phosphorylation by pIkk is considered.

The inspection of reaction fluxes yields negligible fluxes, i.e., elementary processes which can be removed from the reaction network. Another means to reduce the model is the inspection of the parameter profile likelihood. The profile likelihood is computed by fixing a parameter of interest to certain values. For fixed parameter value, the objective function is reoptimized with respect to all other parameters. Compared to the log-likelihood at the original optimum, the new objective value is higher. The difference is denoted as $\Delta\chi^2$. The objective function is composed of two contributions: (1) the log-likelihood of the data and (2) the parameter prior, see eq. (5). Figure S4 shows the result of evaluating the profile likelihood for selected parameters. Investigating the profiles of the parameters `deact_TNFR`, `int_Nfk`, `tau_A20` and `off_IkBa_IP` we fixed those values to 0 ($-\infty$ on log-scale). Reaction rates `ext_nNfkIkb` and `eta_form_complex_nuc` can become arbitrarily large without deteriorating the fit between model and data, reflected by the solid line flattening out towards larger parameter values. We fixed the values of these parameters to 1000 (6.9 on log-scale).

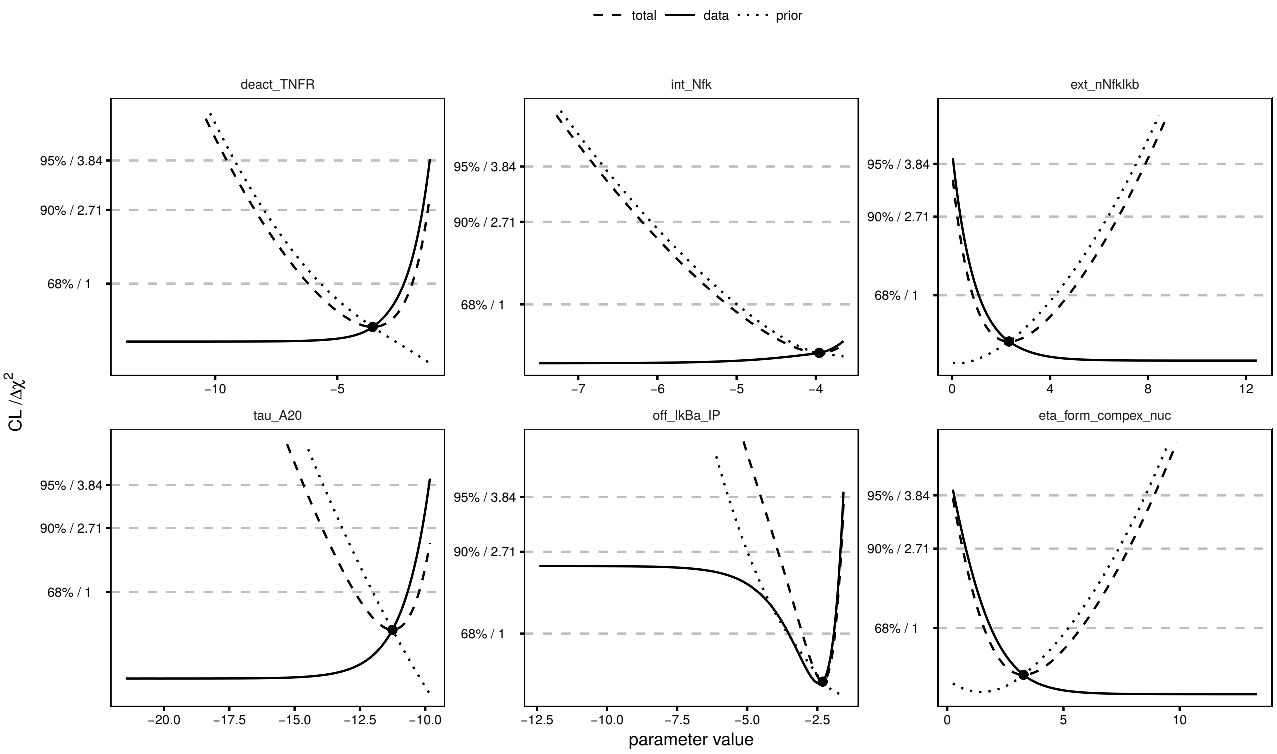

Figure S4: Profile likelihood for selected parameters. The total profile likelihood (dashed line) is composed of a contribution originating from the data points (solid line) and the parameter prior (dotted line).

2.3 The reduced model

Model reduction by flux and profile-likelihood analysis yields the reduced list of reactions as shown in Table S5.

	Educt	→	Product	Rate	Description
1		→	TNFR	uptake · TNF	TNFR act
2	TNFR	→		deact_TNFR · TNFR	TNFR deact
3	Ikk	→	pIkk	act_Ikk_by_TNF · TNFR · Ikk	IKK phos by TNFR
4	pIkk	→	ppIkk	act_pIkk · pIkk	pIKK phos
5	ppIkk	→	iIkk	deact_ppIkk · ppIkk	ppIKK dephos
6	iIkk	→	Ikk	trigger_iIkk · iIkk	IKK triggering
7	NfkIkb	→	NfkpIkb	(act_Ikb_by_Ikk) · pIkk · NfkIkb	Ikb phos in NfkIkb by pIkk
8	pNfkIkb	→	pNfkpIkb	(act_Ikb_complex) · pNfkIkb	Basal Ikb phos in pNfkIkb
9	pNfkIkb	→	pNfkpIkb	(act_Ikb_by_Ikk) · pIkk · pNfkIkb	Ikb phos in pNfkIkb by pIkk
10	NfkIkb	→	pNfkIkb	(act_Nfk_by_Ikk) · pIkk · NfkIkb	Nfk phos in NfkIkb by pIkk
11	NfkpIkb	→	pNfkpIkb	(act_Nfk_by_Ikk_complex) · pIkk · NfkpIkb	Nfk phos in NfkpIkb by pIkk
12	NfkpIkb	→	Nfk + pIkb	split_NfkpIkb · NfkpIkb	Split NfkpIkb
13	pNfkpIkb	→	pNfk + pIkb	split_NfkIkb · pNfkpIkb	Split pNfkpIkb
14	Nfk + Ikb	→	NfkIkb	form_complex · Nfk · Ikb	Form NfkIkb
15		→	mIkb	prod_mIkb_by_nNfk · nNfk	Prod mIkb by nNfk
16	mIkb	→		degrad_mIkb · mIkb	Degrad mIkb
17		→	Ikb	prod_Ikb · mIkb	Prod Ikb
18	pIkb	→		degrad_Ikb · pIkb	Degra pIkb
19	Ikb	→	nIkb	int_Ikb · Ikb	Internalize Ikb
20	pNfk	→	pnNfk	(int_Nfk · eta_int_pNfk) · pNfk	Internalize pNfk
21	Nfk	→	nNfk	(int_Nfk) · Nfk	Internalize Nfk
22	pnNfk	→	nNfk	deact_pnNfk · pnNfk	Dephos pnNfk
23	nIkb + nNfk	→	nNfkIkb	form_complex_nuc · nNfk · nIkb	Form nNkfIkb
24	nNfkIkb	→	NfkIkb	ext_nNfkIkb · nNfkIkb	Externalize nNfkIkb
25		→	RnaA20_1	build_RnaA20 · nNfk	build RNA A20
26	RnaA20_1	→	RnaA20	shuttle_RnaA20 · RnaA20_1	shuttle RNA A20
27	RnaA20	→		degrad_RnaA20 · RnaA20	degrad RNA A20
28		→	A20	build_A20 · RnaA20	Prod A20
29	A20	→		degrad_A20 · A20	Degrad A20

Table S5: List of reactions for the reduced model

Accordingly, the reduced set of ordinary differential equations is shown in Table S6.

$\frac{d}{dt}$TNFR	=	1 · (uptake · TNF) - 1 · (deact_TNFR · TNFR)
$\frac{d}{dt}$Ikk	=	- 1 · (act_Ikk_by_TNF · TNFR · Ikk) + 1 · (trigger_iIkk · iIkk)
$\frac{d}{dt}$pIkk	=	1 · (act_Ikk_by_TNF · TNFR · Ikk) - 1 · (act_pIkk · pIkk)
$\frac{d}{dt}$ppIkk	=	1 · (act_pIkk · pIkk) - 1 · (deact_ppIkk · ppIkk)
$\frac{d}{dt}$iIkk	=	1 · (deact_ppIkk · ppIkk) - 1 · (trigger_iIkk · iIkk)
$\frac{d}{dt}$NfkIkb	=	- 1 · ((act_Ikb_by_Ikk) · pIkk · NfkIkb) - 1 · ((act_Nfk_by_Ikk) · pIkk · NfkIkb) + 1 · (form_complex · Nfk · Ikb) + 1 · (ext_nNfkIkb · nNfkIkb) · (Vnuc / 1)
$\frac{d}{dt}$NfkpIkb	=	1 · ((act_Ikb_by_Ikk) · pIkk · NfkIkb) - 1 · ((act_Nfk_by_Ikk_complex) · pIkk · NfkpIkb) - 1 · (split_NfkpIkb · NfkpIkb)
$\frac{d}{dt}$pNfkIkb	=	- 1 · ((act_Ikb_complex) · pNfkIkb) - 1 · ((act_Ikb_by_Ikk) · pIkk · pNfkIkb) + 1 · ((act_Nfk_by_Ikk) · pIkk · NfkIkb)
$\frac{d}{dt}$pNfkpIkb	=	1 · ((act_Ikb_complex) · pNfkIkb) + 1 · ((act_Ikb_by_Ikk) · pIkk · pNfkIkb) + 1 · ((act_Nfk_by_Ikk_complex) · pIkk · NfkpIkb) - 1 · (split_NfkIkb · pNfkpIkb)
$\frac{d}{dt}$pNfk	=	1 · (split_NfkIkb · pNfkpIkb) - 1 · ((int_Nfk · eta_int_pNfk) · pNfk)
$\frac{d}{dt}$Nfk	=	1 · (split_NfkpIkb · NfkpIkb) - 1 · (form_complex · Nfk · Ikb) - 1 · ((int_Nfk) · Nfk)
$\frac{d}{dt}$pIkb	=	1 · (split_NfkpIkb · NfkpIkb) + 1 · (split_NfkIkb · pNfkpIkb) - 1 · (degrad_Ikb · pIkb)
$\frac{d}{dt}$Ikb	=	- 1 · (form_complex · Nfk · Ikb) + 1 · (prod_Ikb · mIkb) - 1 · (int_Ikb · Ikb)
$\frac{d}{dt}$mIkb	=	1 · (prod_mIkb_by_nNfk · nNfk) - 1 · (degrad_mIkb · mIkb)
$\frac{d}{dt}$nIkb	=	1 · (int_Ikb · Ikb) · (1 / Vnuc) - 1 · (form_complex_nuc · nNfk · nIkb)
$\frac{d}{dt}$pnNfk	=	1 · ((int_Nfk · eta_int_pNfk) · pNfk) · (1 / Vnuc) - 1 · (deact_pnNfk · pnNfk)
$\frac{d}{dt}$nNfk	=	1 · ((int_Nfk) · Nfk) · (1 / Vnuc) + 1 · (deact_pnNfk · pnNfk) - 1 · (form_complex_nuc · nNfk · nIkb)
$\frac{d}{dt}$nNfkIkb	=	1 · (form_complex_nuc · nNfk · nIkb) - 1 · (ext_nNfkIkb · nNfkIkb)
$\frac{d}{dt}$RnaA20_1	=	1 · (build_RnaA20 · nNfk) - 1 · (shuttle_RnaA20 · RnaA20_1)
$\frac{d}{dt}$RnaA20	=	1 · (shuttle_RnaA20 · RnaA20_1) - 1 · (degrad_RnaA20 · RnaA20)
$\frac{d}{dt}$A20	=	1 · (build_A20 · RnaA20) - 1 · (degrad_A20 · A20)

Table S6: List of differential equations of the reduced model

Upon parameter estimation, the profile likelihood as been computed for all parameters veryfying that all model parameters can be constrained within finite confidence intervals. The profile likelihood is shown in Figure S5.

Based on the profiles, the parameter values and their lower and upper bounds according to a 95% confidence level were extracted. The result is shown in Table S7.

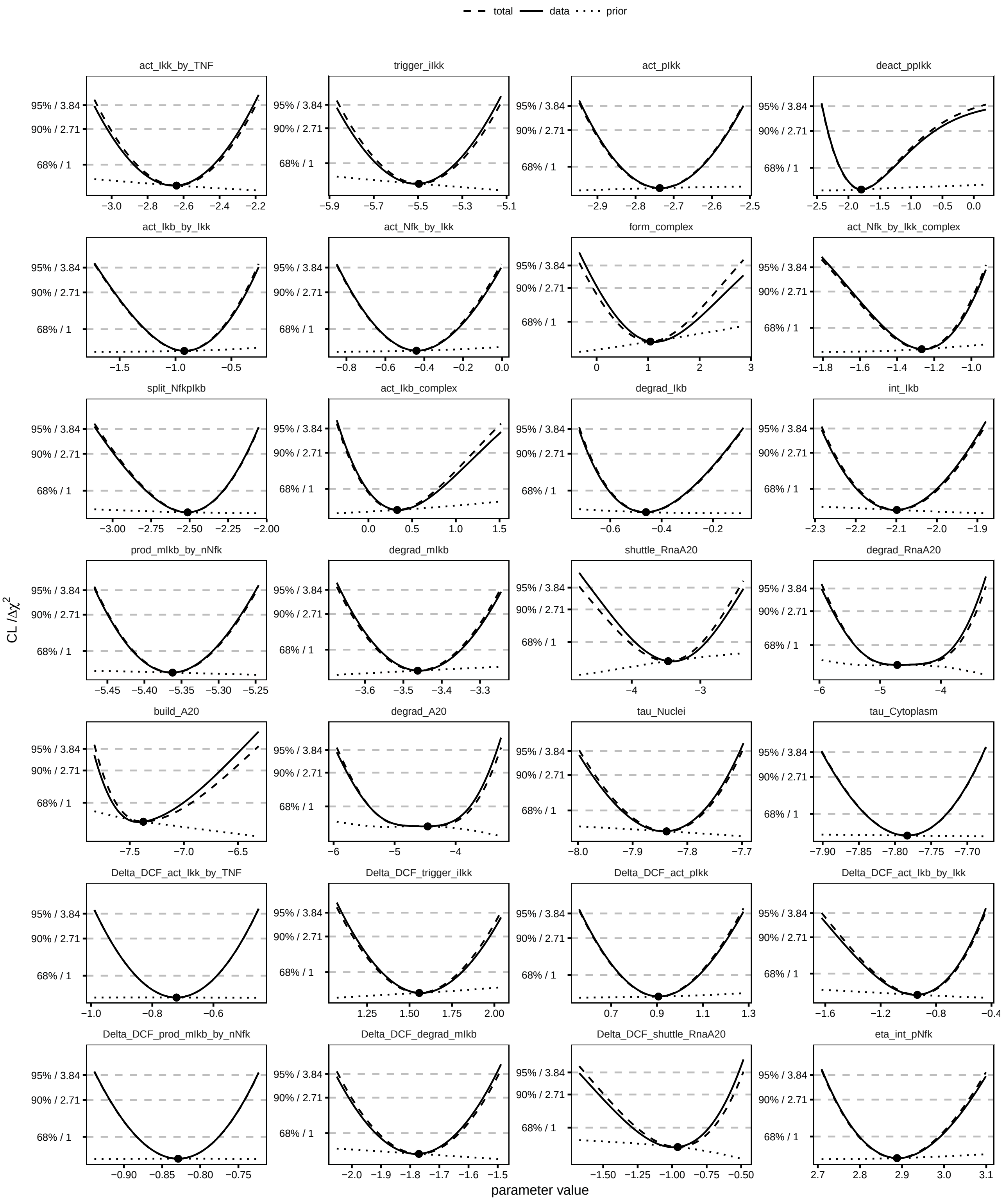

Figure S5: Profile likelihood for the reduced model.

	value	lower (95%)	upper (95%)
act_Ikb_by_Ikk	-0.92	-1.70	-0.26
act_Ikb_complex	0.33	-0.33	1.56
act_Ikk_by_TNF	-2.64	-3.10	-2.21
act_Nfk_by_Ikk	-0.44	-0.84	-0.00
act_Nfk_by_Ikk_complex	-1.27	-1.76	-0.92
act_pIkk	-2.74	-2.94	-2.52
build_A20	-7.38	-7.84	-6.46
deact_ppIkk	-1.80	-2.42	0.42
degrad_A20	-4.46	-5.97	-3.32
degrad_Ikb	-0.46	-0.72	-0.08
degrad_mIkb	-3.46	-3.66	-3.24
degrad_RnaA20	-4.72	-5.97	-3.32
Delta_DCF_act_Ikb_by_Ikk	-0.94	-1.66	-0.45
Delta_DCF_act_Ikk_by_TNF	-0.72	-0.98	-0.46
Delta_DCF_act_pIkk	0.91	0.57	1.27
Delta_DCF_degrad_mIkb	-1.77	-2.06	-1.50
Delta_DCF_prod_mIkb_by_nNfk	-0.83	-0.94	-0.73
Delta_DCF_shuttle_RnaA20	-0.96	-1.68	-0.52
Delta_DCF_trigger_iIkk	1.56	1.10	2.05
eta_int_pNfk	2.89	2.71	3.10
form_complex	1.04	-0.21	3.03
int_Ikb	-2.10	-2.29	-1.89
prod_mIkb_by_nNfk	-5.36	-5.47	-5.25
shuttle_RnaA20	-3.47	-4.60	-2.36
split_NfkpIkb	-2.51	-3.11	-2.06
tau_Cytoplasm	-7.78	-7.90	-7.68
tau_Nuclei	-7.84	-8.00	-7.70
trigger_iIkk	-5.50	-5.87	-5.15

Table S7: List of estimated parameters with lower and upper 95% confidence bounds.

Tables S8 and S9 show the values of the untransformed parameters, i.e., the parameter values as they enter in numeric integration of the model ODEs. Those parameters coinciding with state names refer to initial value parameters whereas all other parameters are rate constants and dynamic parameters.

	no+TNF	DCF+TNF	
A20	0.0000	0.0000	
act_Ikb_by_Ikk	0.3980	0.1562	*
act_Ikb_complex	1.3897	1.3897	
act_Ikk_by_TNF	0.0714	0.0347	*
act_Nfk_by_Ikk	0.6438	0.6438	
act_Nfk_by_Ikk_complex	0.2816	0.2816	
act_pIkk	0.0648	0.1603	*
build_A20	0.0006	0.0006	
build_RnaA20	1.0000	1.0000	
deact_pnNfk	1000.0000	1000.0000	
deact_ppIkk	0.1660	0.1660	
deact_TNFR	0.0010	0.0010	
degrad_A20	0.0116	0.0116	
degrad_Ikb	0.6308	0.6308	
degrad_mIkb	0.0313	0.0053	*
degrad_RnaA20	0.0089	0.0089	
eta_int_pNfk	17.9585	17.9585	
ext_nNfkIkb	1000.0000	1000.0000	
form_complex	2.8390	2.8390	
form_complex_nuc	1000.0000	1000.0000	
iIkk	0.0000	0.0000	
Ikb	0.0000	0.0000	
Ikk	1.0000	1.0000	
int_Ikb	0.1226	0.1226	
int_Nfk	0.0100	0.0100	
mIkb	0.0000	0.0000	
Nfk	0.0000	0.0000	
NfkIkb	1.0000	1.0000	
NfkpIkb	0.0000	0.0000	
nIkb	0.0000	0.0000	
nNfk	0.0000	0.0000	
nNfkIkb	0.0000	0.0000	
off_A20	0.6623	0.6623	
off_Cytoplasm	0.7859	0.7859	
off_IkBa	0.0000	0.0000	
off_IkBa_IP	0.0000	0.0000	
off_Nuclei	0.9765	0.9765	
off_p65_IP	0.8554	0.8554	
off_pIkBa	0.2278	0.2278	
off_pIkBalpha_tp65_IP	0.2036	0.2036	
off_pIKK	0.4393	0.4393	
off_pp65	0.2969	0.2969	
off_pp65_IP	0.4303	0.4303	
off_tIkBalpha_pp65_IP	0.4437	0.4437	
off_tIkBalpha_tp65_IP	0.2716	0.2716	
pIkb	0.0000	0.0000	

Table S8: List of model parameters (part 1) as being employed for numeric integration of the model ODEs. Parameters differing between the no-DCF and DCF conditions are marked by asterisks in the last column.

	no+TNF	DCF+TNF	
pIkk	0.0000	0.0000	
pNfk	0.0000	0.0000	
pNfkIkb	0.0000	0.0000	
pNfkpIkb	0.0000	0.0000	
pnNfk	0.0000	0.0000	
ppIkk	0.0000	0.0000	
prod_Ikb	1.0000	1.0000	
prod_mIkb_by_nNfk	0.0047	0.0020	*
RnaA20	0.0000	0.0000	
RnaA20_1	0.0000	0.0000	
s_A20	1.0000	1.0000	
s_Cytoplasm	0.3244	0.3244	
s_IkBa	1.1868	1.1868	
s_IkBa_IP	1.7393	1.7393	
s_Nuclei	1.1236	1.1236	
s_p65_IP	0.1694	0.1694	
s_pIkBa	5.5684	5.5684	
s_pIkBalpha_tp65_IP	6.5120	6.5120	
s_pIKK	2.4711	2.4711	
s_pp65	4.9452	4.9452	
s_pp65_IP	2.1543	2.1543	
s_tIkBalpha_pp65_IP	4.6047	4.6047	
s_tIkBalpha_tp65_IP	1.3426	1.3426	
shuttle_RnaA20	0.0311	0.0119	*
sigma_A20	0.0585	0.0585	
sigma_Cytoplasm	0.0251	0.0251	
sigma_Nuclei	0.0294	0.0294	
split_NfkIkb	1.0000	1.0000	
split_NfkpIkb	0.0811	0.0811	
tau_A20	0.0000	0.0000	
tau_Cytoplasm	0.0004	0.0004	
tau_Nuclei	0.0004	0.0004	
TNF	1.0000	1.0000	
TNFR	0.0000	0.0000	
total_IKK	1.0000	1.0000	
total_NFKB	1.0000	1.0000	
trigger_iIkk	0.0041	0.0195	*
uptake	1.0000	1.0000	
Vnuc	1.0000	1.0000	

Table S9: List of model parameters (part 2) as being employed for numeric integration of the model ODEs. Parameters differing between the no-DCF and DCF conditions are marked by asterisks in the last column.

3 Impact of DCF on the TNFa/TNFR interaction in HepG2 cells

**Pre-treatment**	**Stimulation time**	**Stimulation**	**TNFR1 [clusters/µm²]**	**Cluster radius (DBSCAN) [nm]**
**- DCF**	0 min	-	1.1 ± 0.1	28.7 ± 0.4
	2 min	-	1.0 ± 0.1	26.7 ± 0.4
		TNFα	1.0 ± 0.1	28.7 ± 0.5
	5 min	-	1.1 ± 0.2	27.1 ± 0.4
		TNFα	0.9 ± 0.1	29.3 ± 0.5
	10 min	-	0.9 ± 0.1	26.2 ±0.4
		TNFα	1.2 ± 0.3	31.1 ± 0.6
	negative control		0.2 ± 0	-
**+ DCF**	0 min	-	1.1 ± 0.1	28.8 ± 0.4
	2 min	-	1.0 ± 0.1	32.4 ± 0.7
		TNFα	1.8 ± 0.3	26.8 ± 0.4
	5 min	-	1.2 ± 0.2	28.2 ± 0.4
		TNFα	0.9 ± 0.1	29.4 ± 0.7
	10 min	-	1.0 ± 0.2	30.5 ± 0.7
		TNFα	1.1 ± 0.1	28.4 ± 0.5
	negative control		0.1 ± 0	-

Table S10: Density of TNFR1 clusters on the membrane of HepG2 cells without and with treatment with DCF. Cells were stimulated with TNFa with incubation times of 2 min, 5 min and 10 min. Cells stained with secondary antibody only were used as a negative control. Cluster radii were obtained from DBSCAN analysis.

4 Predicted A20 dynamics for DILI compounds

The simplified mechanism of action of diclofenac (DCF) allowed to estimate drug-specific parameters enabling the prediction of nuclear NFκB dynamics, see main text. We found that DCF could possibly modify A20 production and that the delayed dynamics of nuclear NFκB is not sufficient to correctly describe the A20 dynamics.

In order to investigate how the A20 dynamics could be impacted by the DILI compounds amiodarone (AMD), acetaminophen (APAP), ximelagatran (XIM) and fialuridine (FIAU), we assumed that either A20 production is affected in the same way as for DCF (direct impact) or A20 production is only altered due to the different dynamics of nuclear NFκB (mediated impact). For both scenarios, A20 time courses are predicted, as shown in Figure S6.

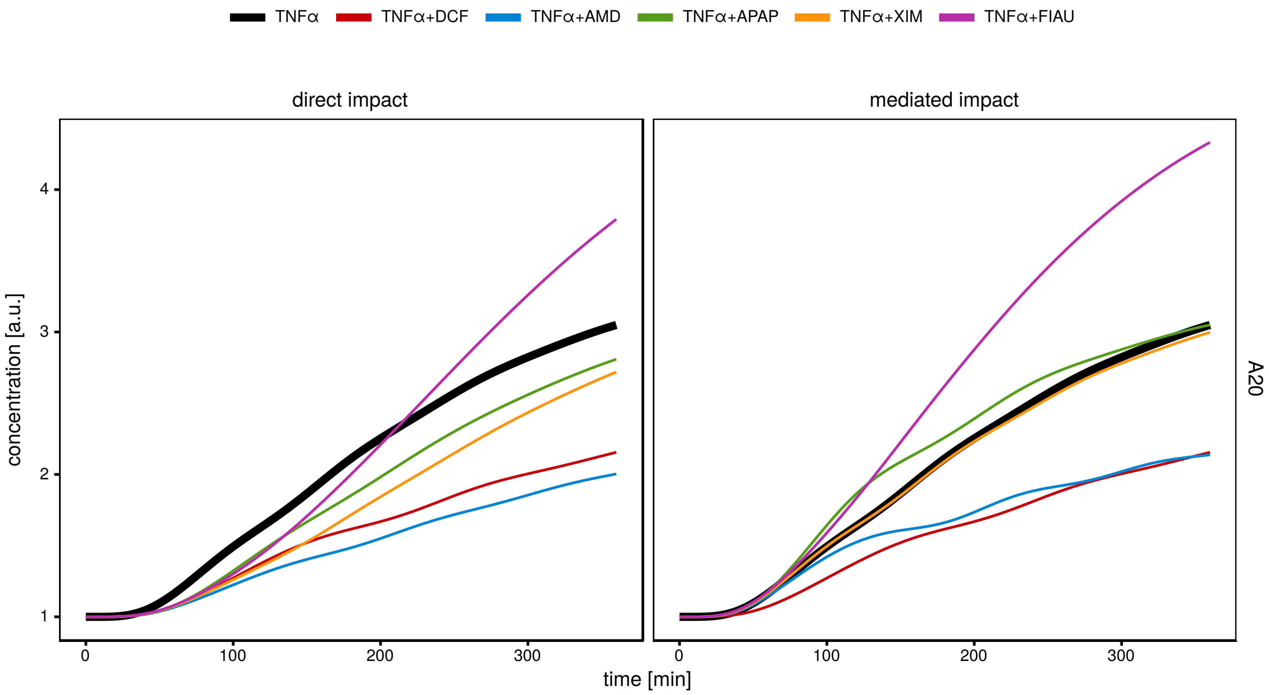

Figure S6: Predicted A20 dynamics. The dynamics of A20 was simulated for different DILI compounds, indicated by different colors, and two scenarios of how the DILI compounds interact with A20.

Both scenarios show that AMD is likely to affect the A20 dynamics in a similar way as DCF. In contrast, APAP and XIM have a smaller inhibitory effect on A20. Finally, FIAU could even increase the production of A20.

5 Data availability

All data used for model calibration is available as separate CSV file:

`data_TNFa_171110.csv`

Table S11: Data used for modeling is available in a separate CSV file. The column description is as follows: **name** (Name of the measured target according to names in the manuscript), **time** (Time in minutes value Measured value on log scale sigma Uncertainty of the measurement), **condition** (Unique identifier of the measurement condition), **treatment** (Treatment of the cells: no, TNF), **compound** (Compound: no, DCF, AMD, APAP, FIAU, XIM) **cell** (Cell type: HepG2, PHH) **scale** (Scale of the measurement: 1, 2, 3. If observations agree by name and scale, their values can be directly compared. observations with the same name but different scale have no common point of reference. Their values are shifted by an unknown offset (log-values), corresponding to an unknown scaling factor on nominal scale.)

6 Dose response curves

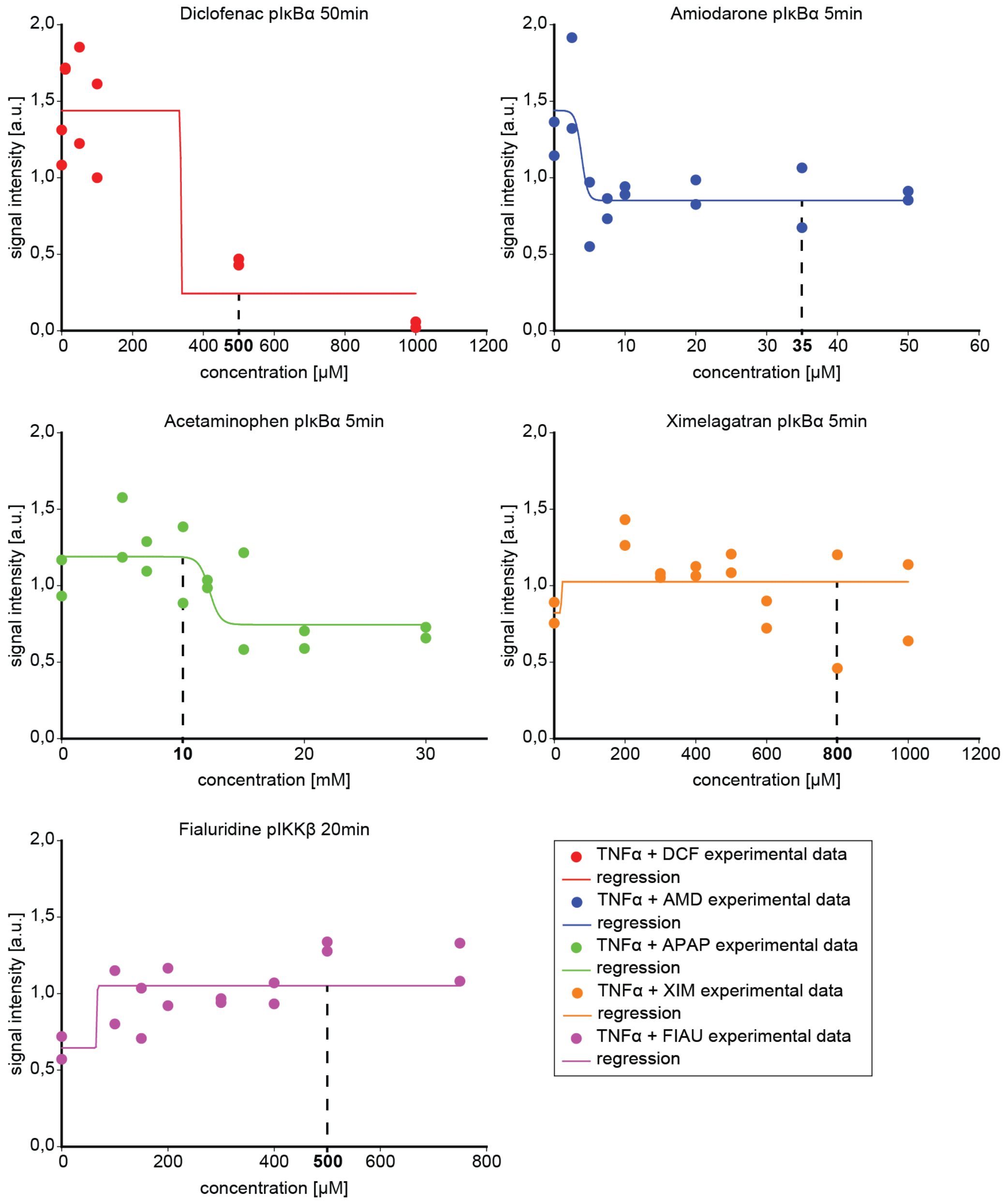

Figure S7: Dose response. IκBα phosphorylation was determined at different time points characteristic for different doses of diclofenac, amiodarone, acetaminophen and ximelagatran. $IKK\beta$ phosphorylation was determined for different doses of fialuridine. Data is shown as dots. Model fits by a non-linear regression model are shown as solid lines.

7 Quantitative immunoblot data

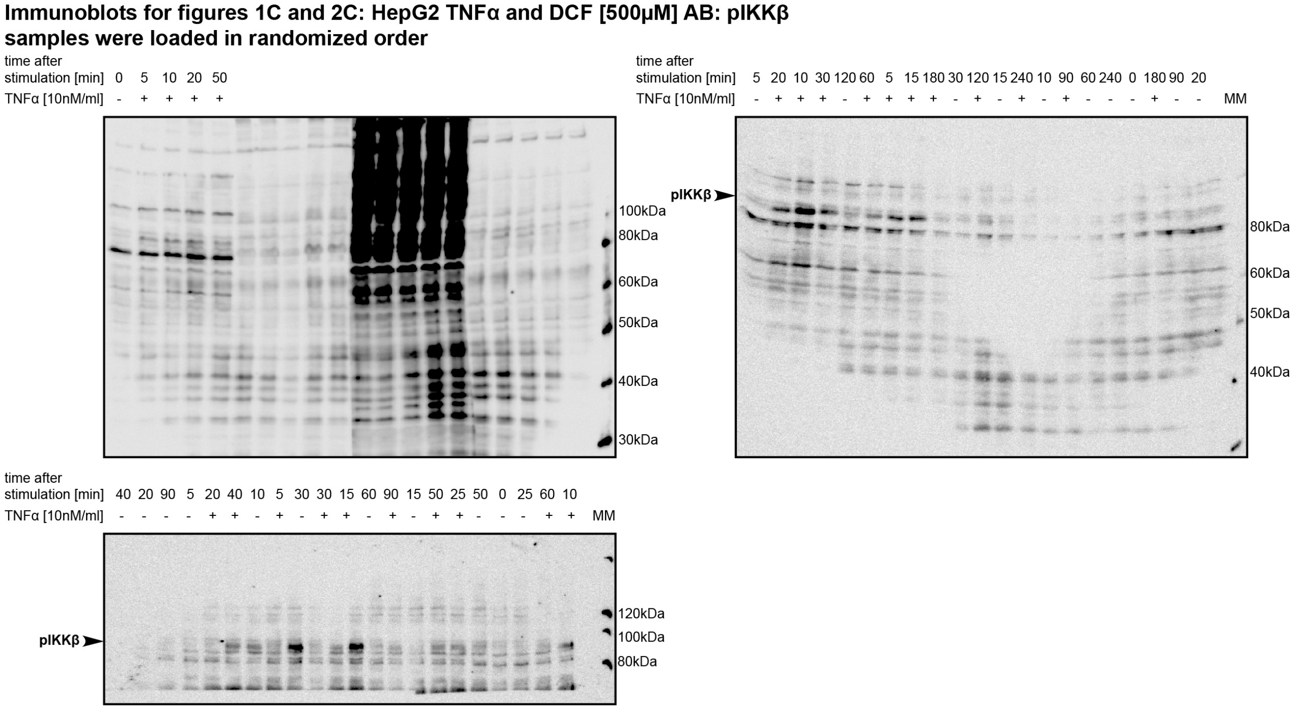

Figure S8: IKK phosphorylation in HepG2 cells.

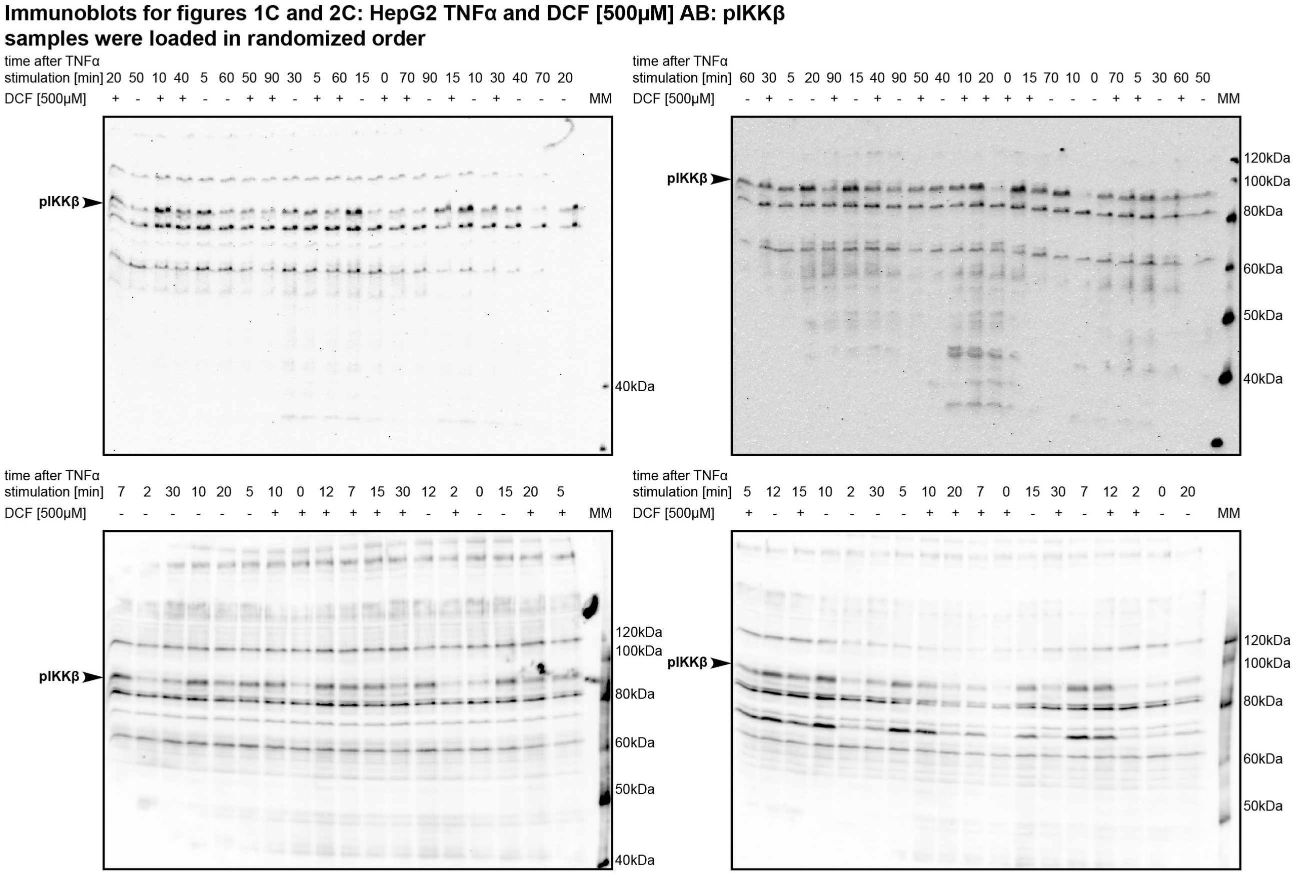

Figure S9: IKK phosphorylation in HepG2 cells.

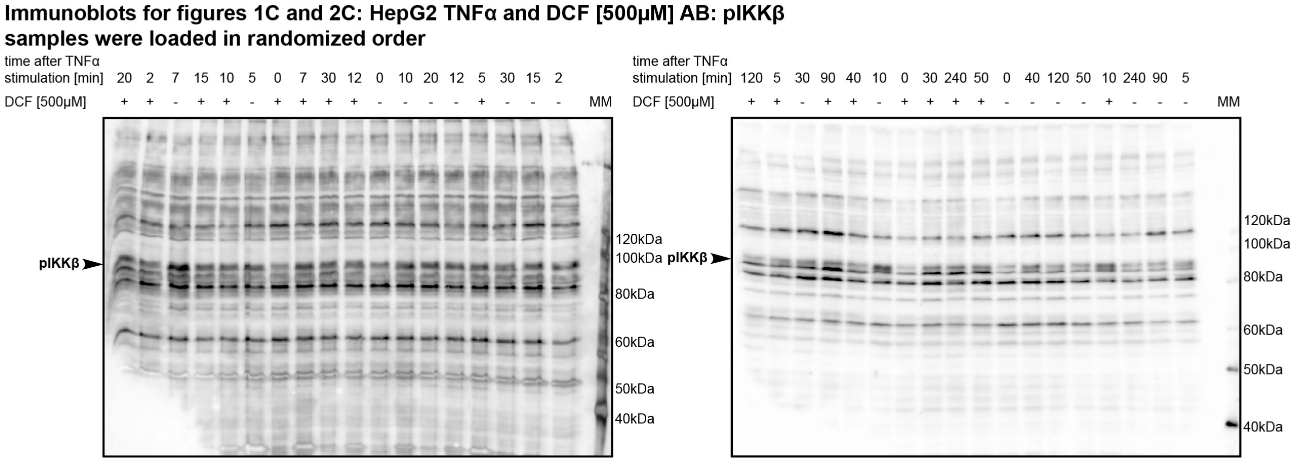

Figure S10: IKK phosphorylation in HepG2 cells.

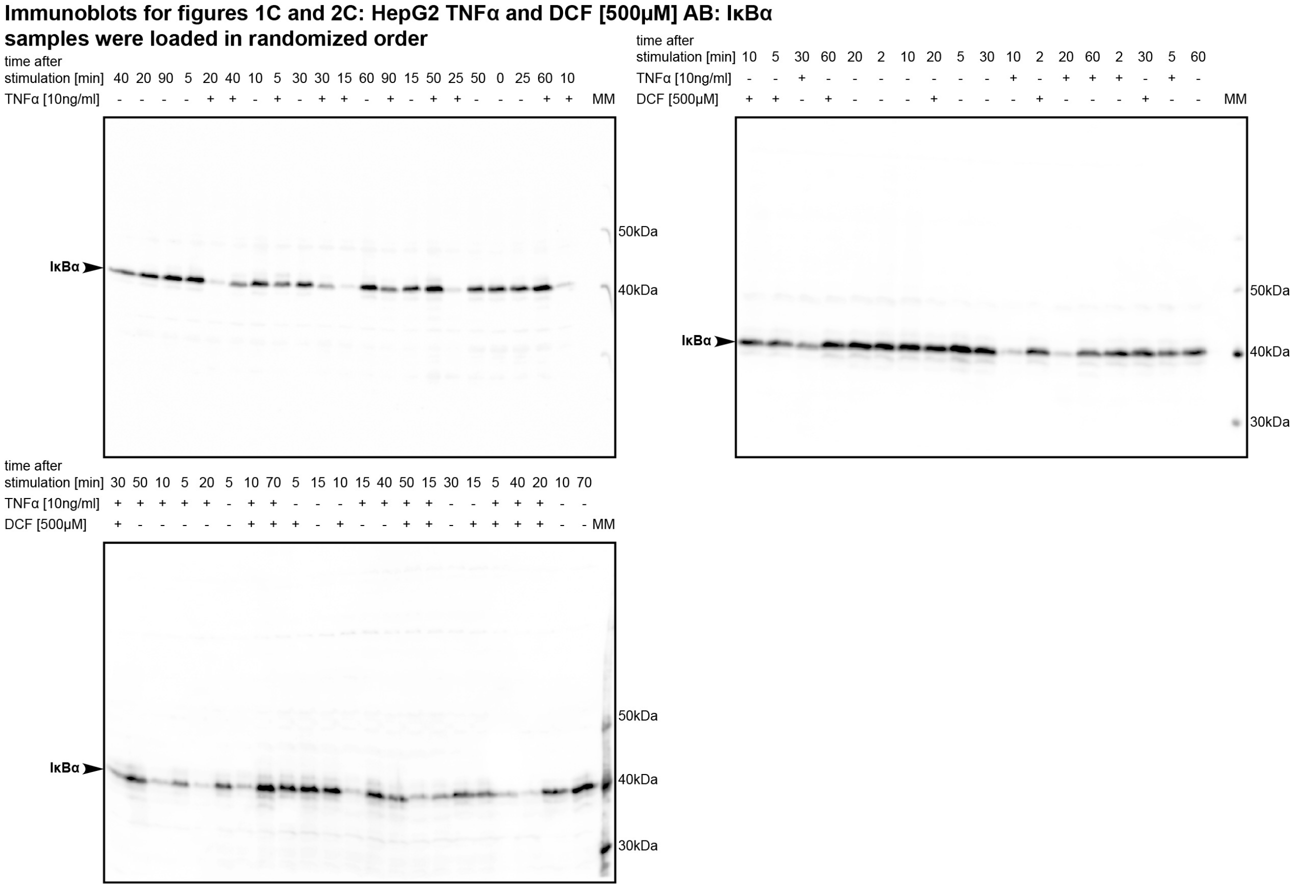

Figure S11: IκBα in HepG2 cells.

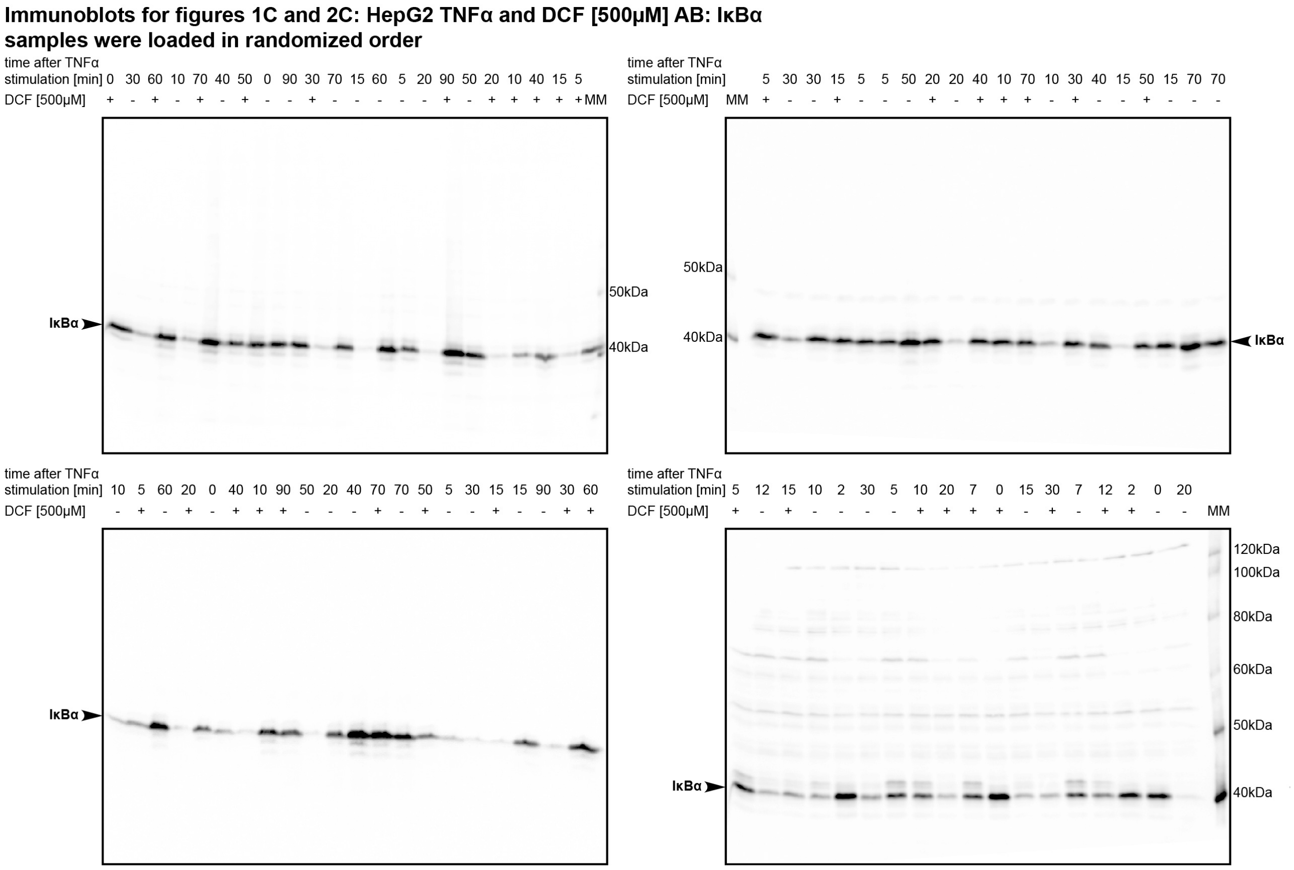

Figure S12: IκBα in HepG2 cells.

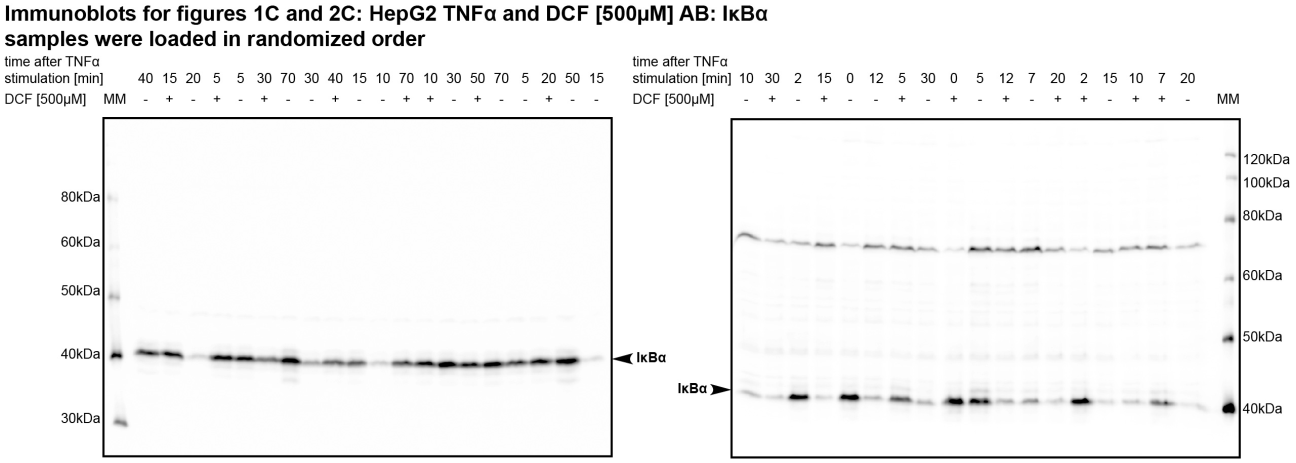

Figure S13: IκBα in HepG2 cells.

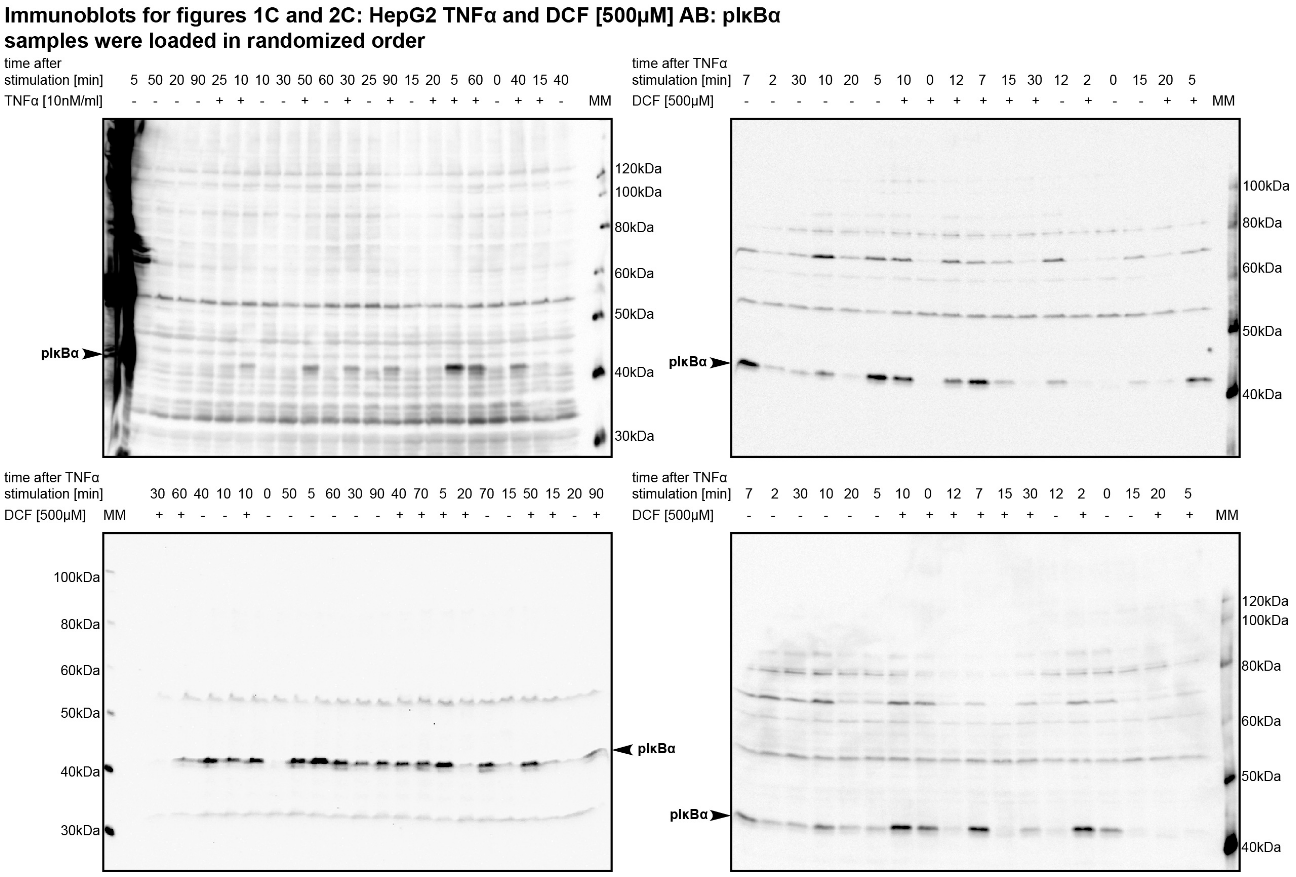

Figure S14: IκBα phosphorylation in HepG2 cells.

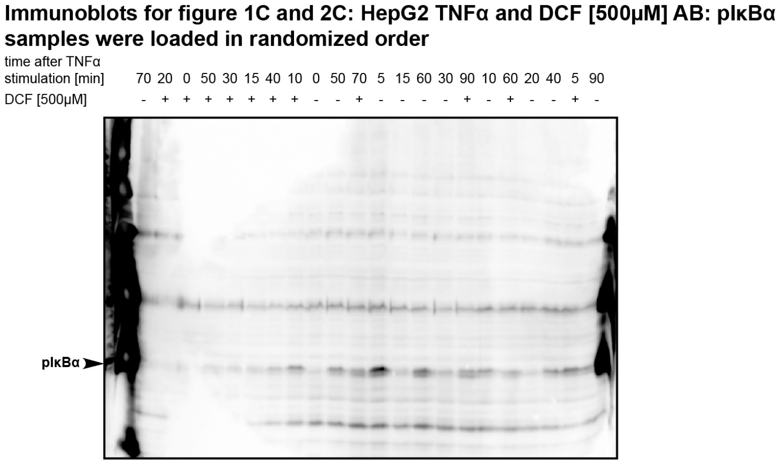

Figure S15: IκBα phosphorylation in HepG2 cells.

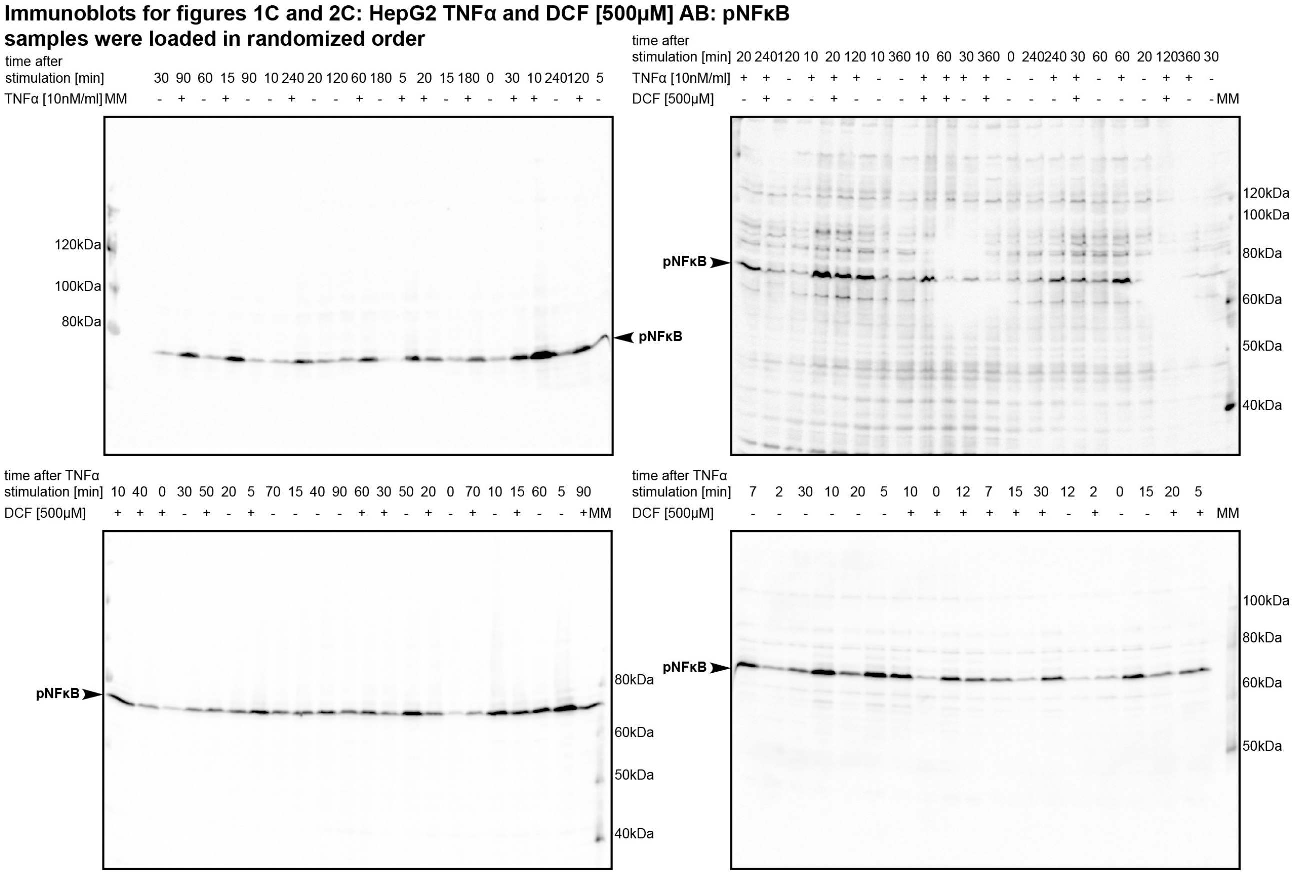

Figure S16: NFκB phosphorylation in HepG2 cells.

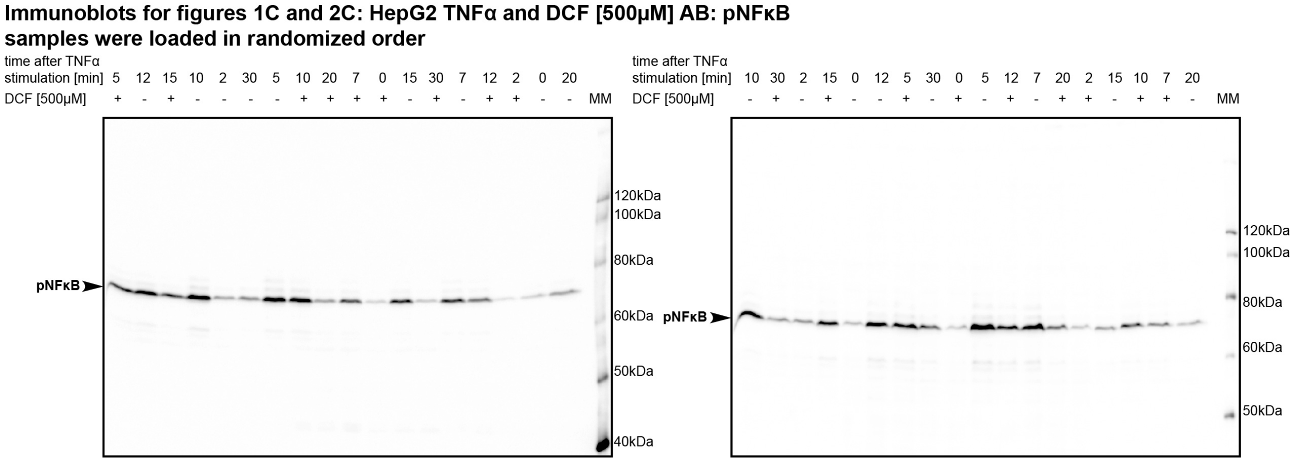

Figure S17: NFκB phosphorylation in HepG2 cells.

Immunoblots for figures 1D and 3: Primary human hepatocytes (PHH) TNFα and DCF [500μM] AB: pIKKβ samples were loaded in randomized order

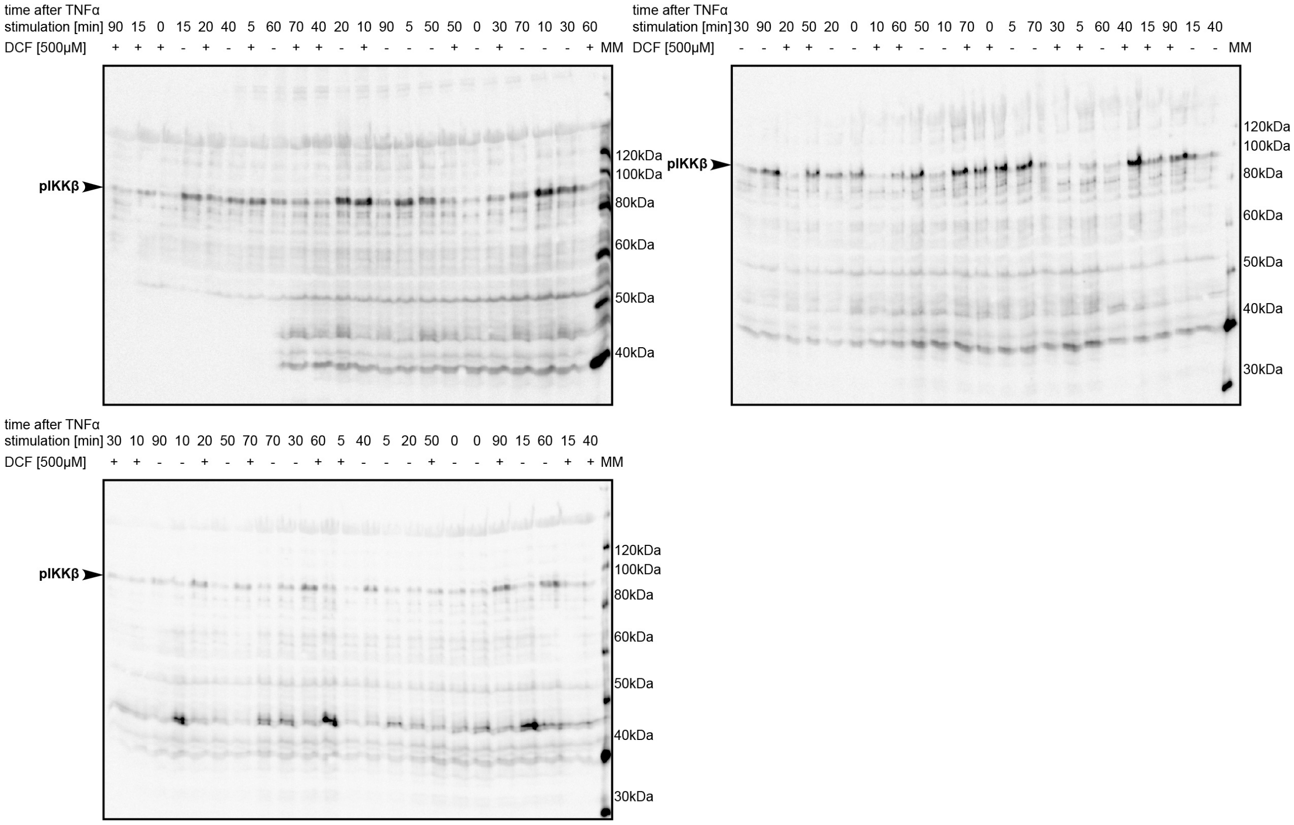

Figure S18: Primary Human Hepatocytes.

Immunoblots for figures 1D and 3: Primary human hepatocytes (PHH) TNFα and DCF [500µM] AB: IκBα samples were loaded in randomized order

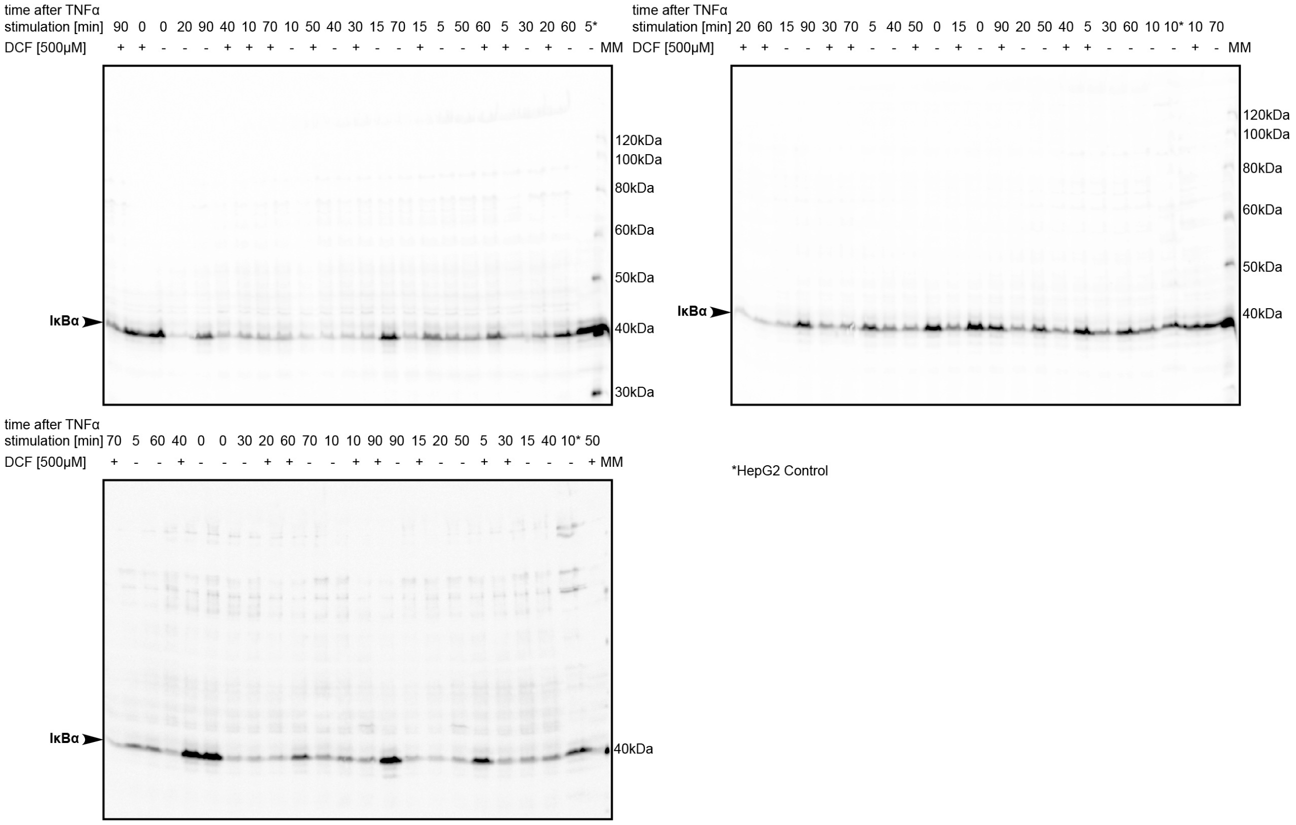

Figure S19: Primary Human Hepatocytes.

Immunoblots for figures 1D and 3: Primary human hepatocytes (PHH) TNFα and DCF [500µM] AB: pIκBα samples were loaded in randomized order

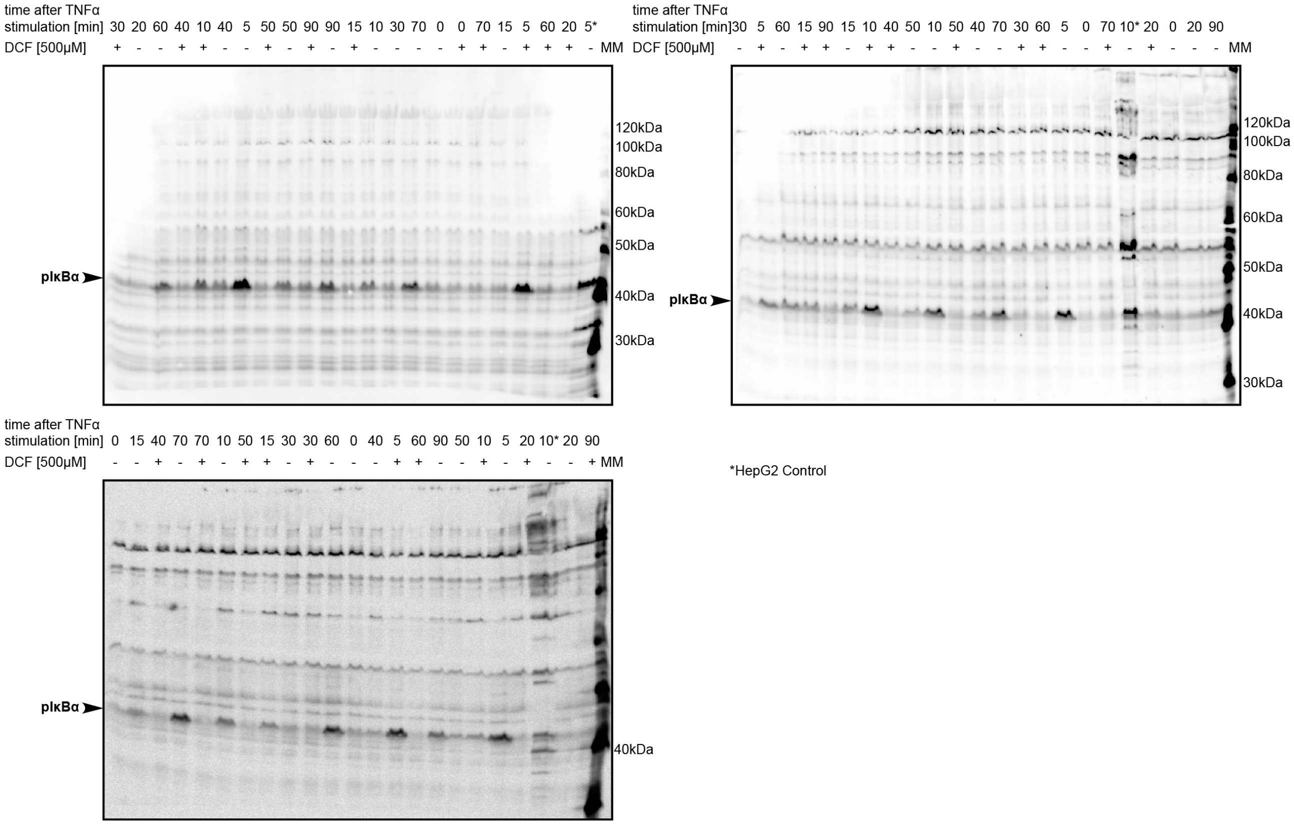

Figure S20: Primary Human Hepatocytes.

Immunoblots for figures 1D and 3: Primary human hepatocytes (PHH) TNFα and DCF [500μM] AB: pNFκB samples were loaded in randomized order

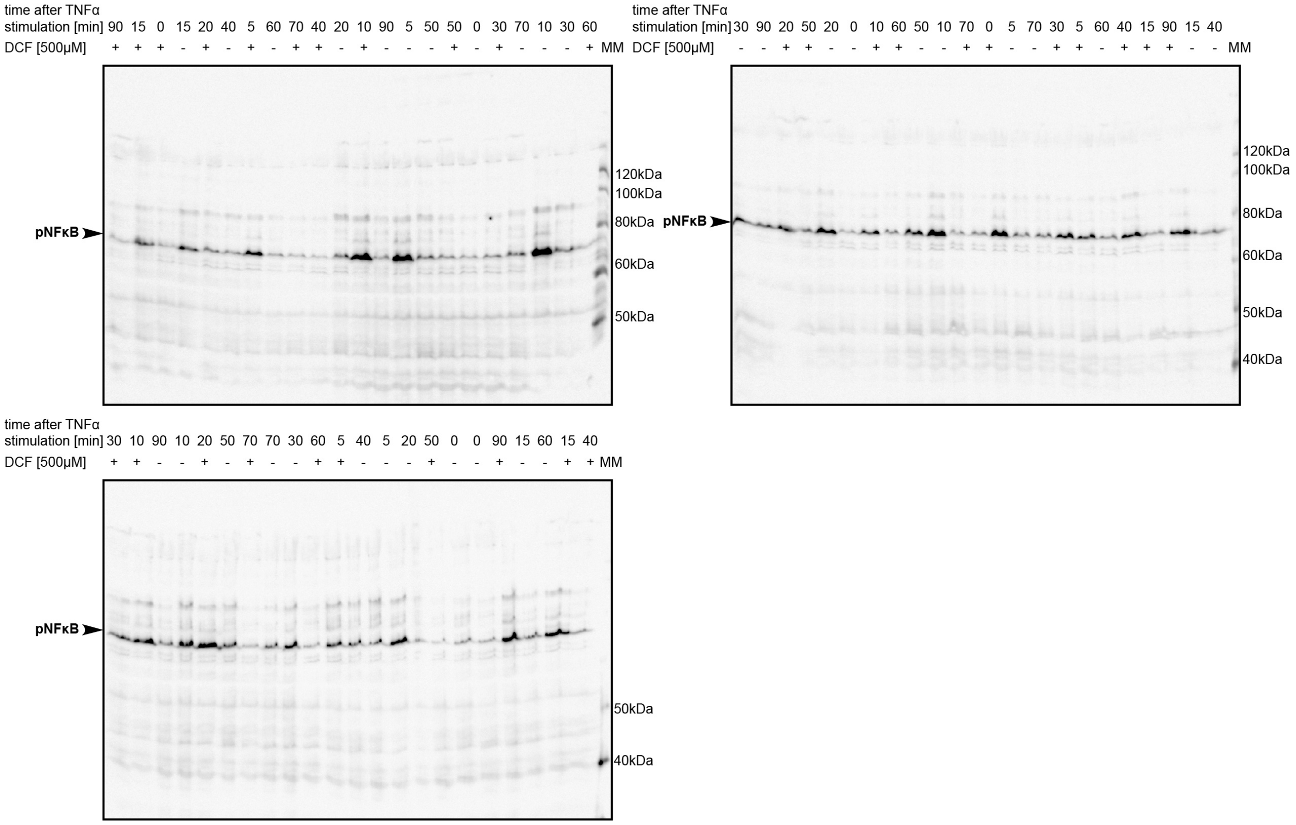

Figure S21: Primary Human Hepatocytes.

Immunoblots for figures 1E and 2C: HepG2 TNFα and DCF [500μM] IP: IκBα IB: IκBα
samples were loaded in randomized order

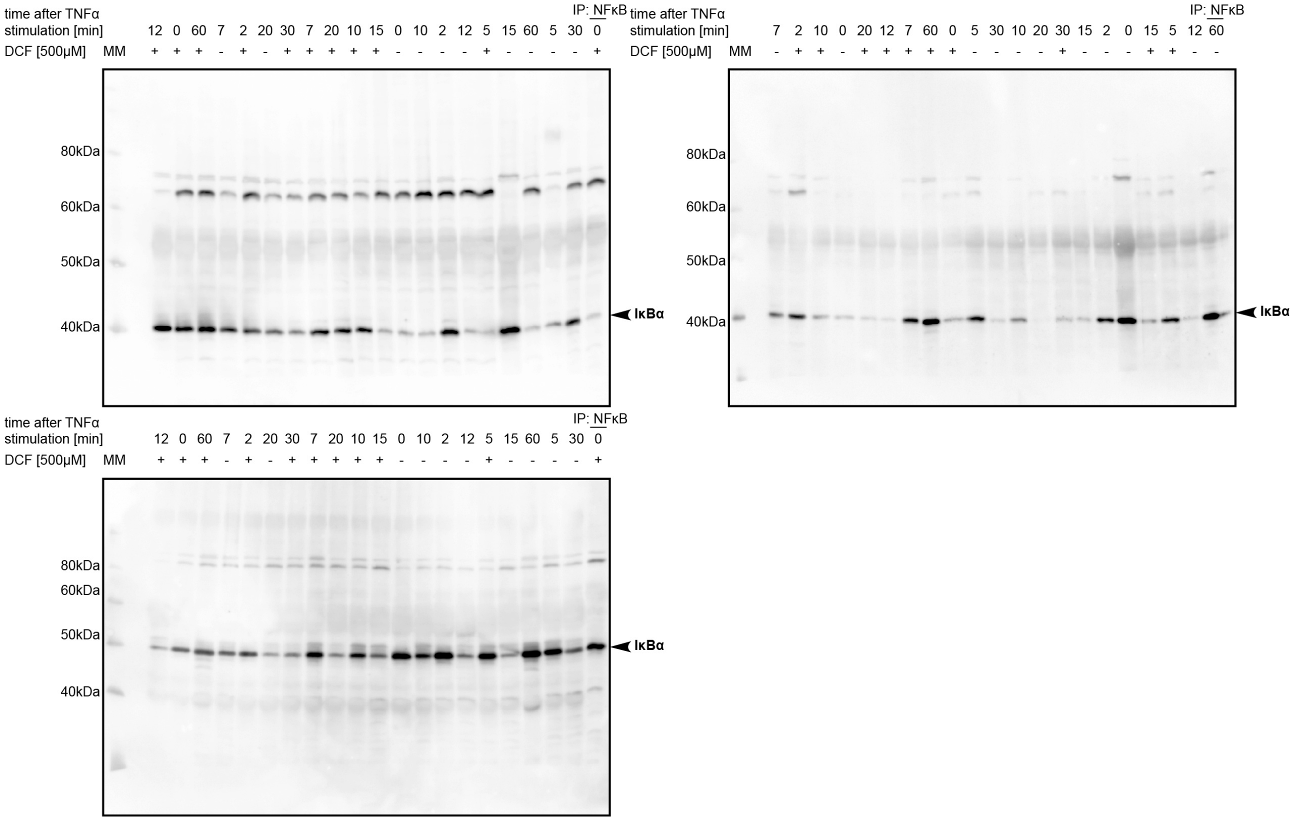

Figure S22: IκBα in Co-IP.

Immunoblots for figures 1E and 2C: HepG2 TNFα and DCF [500μM] IP: IκBα IB: pIκBα
samples were loaded in randomized order

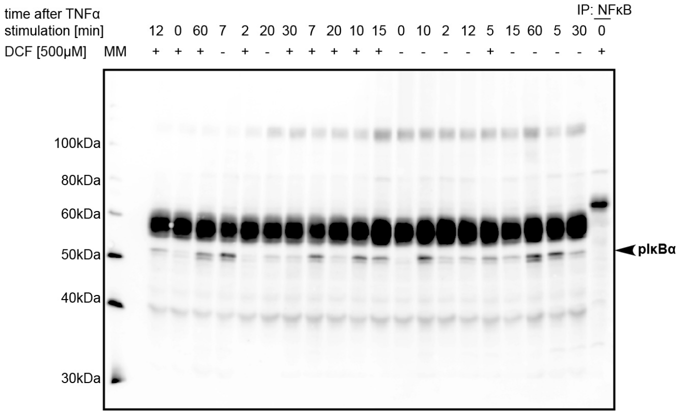

Figure S23: IκBα in Co-IP.

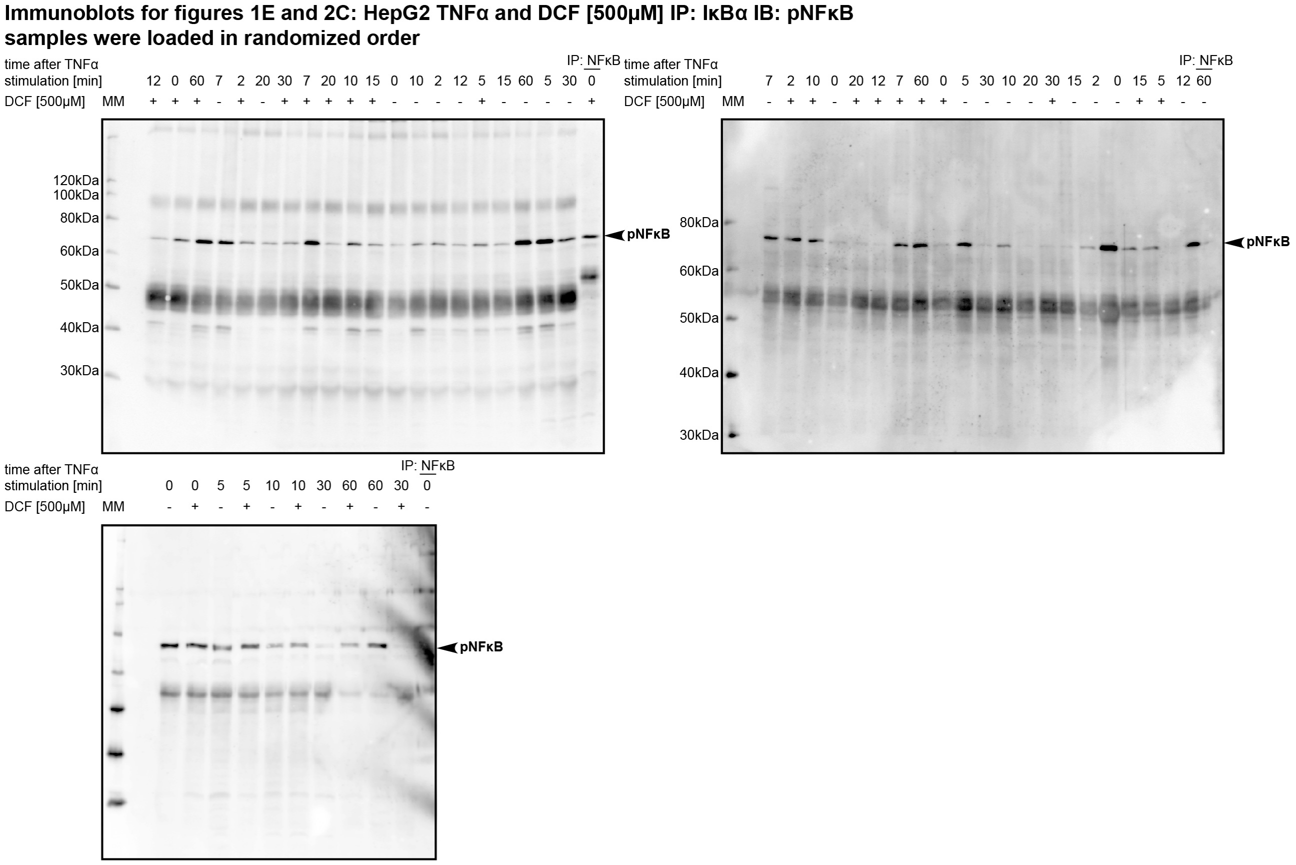

Figure S24: IκBα in Co-IP.

Immunoblots for figures 1E and 2C: HepG2 TNFα and DCF [500μM] IP: IκBα IB: NFκB
samples were loaded in randomized order

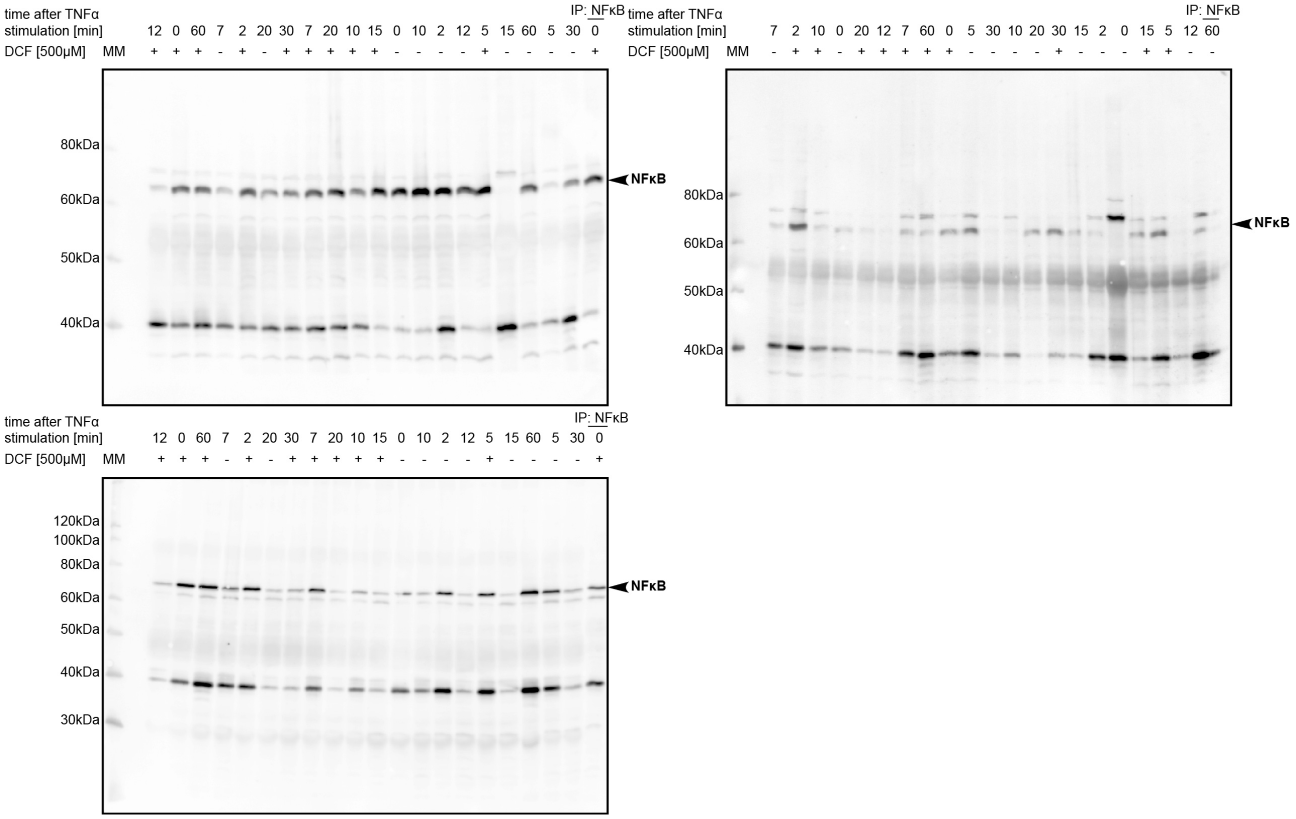

Figure S25: IκBα in Co-IP.

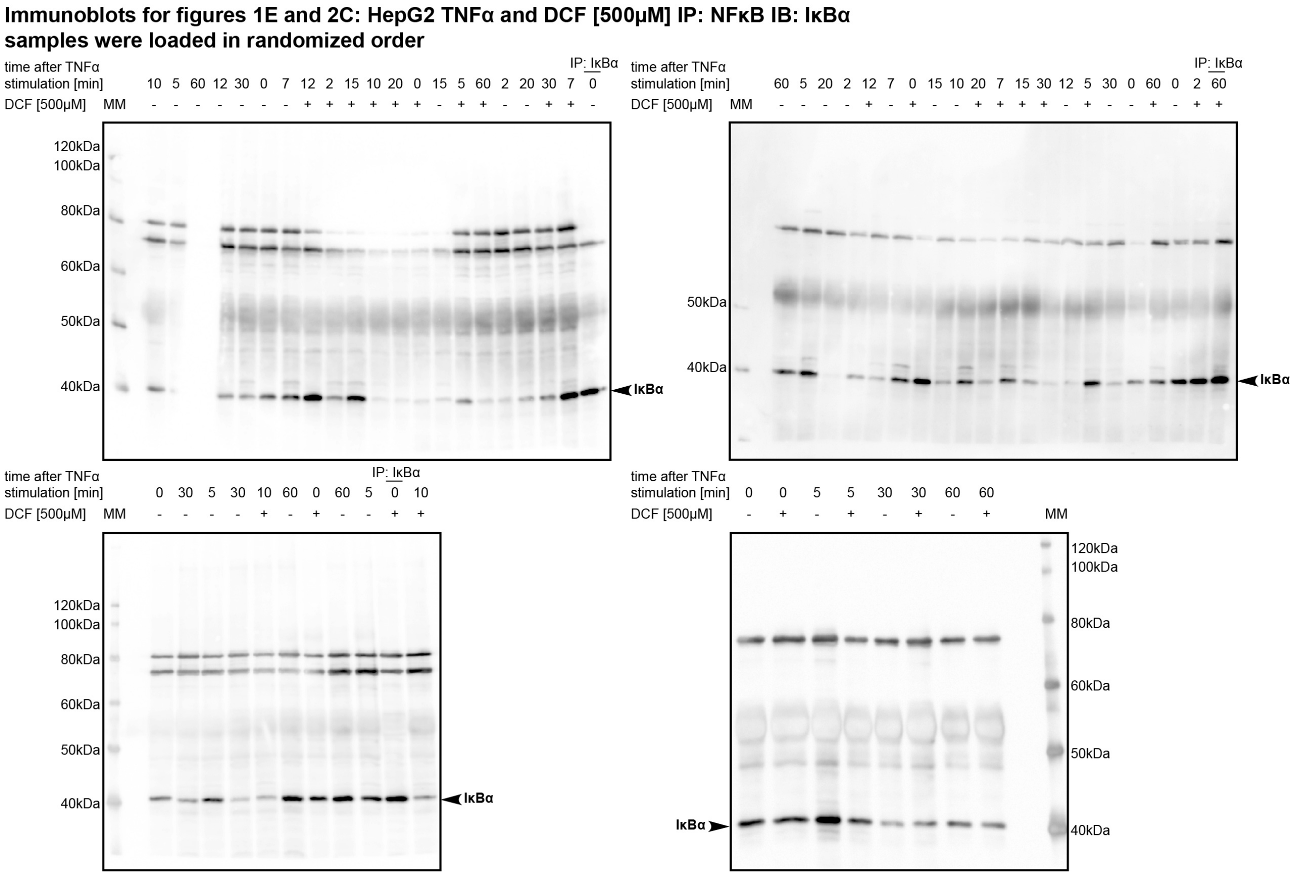

Figure S26: NFκB in Co-IP.

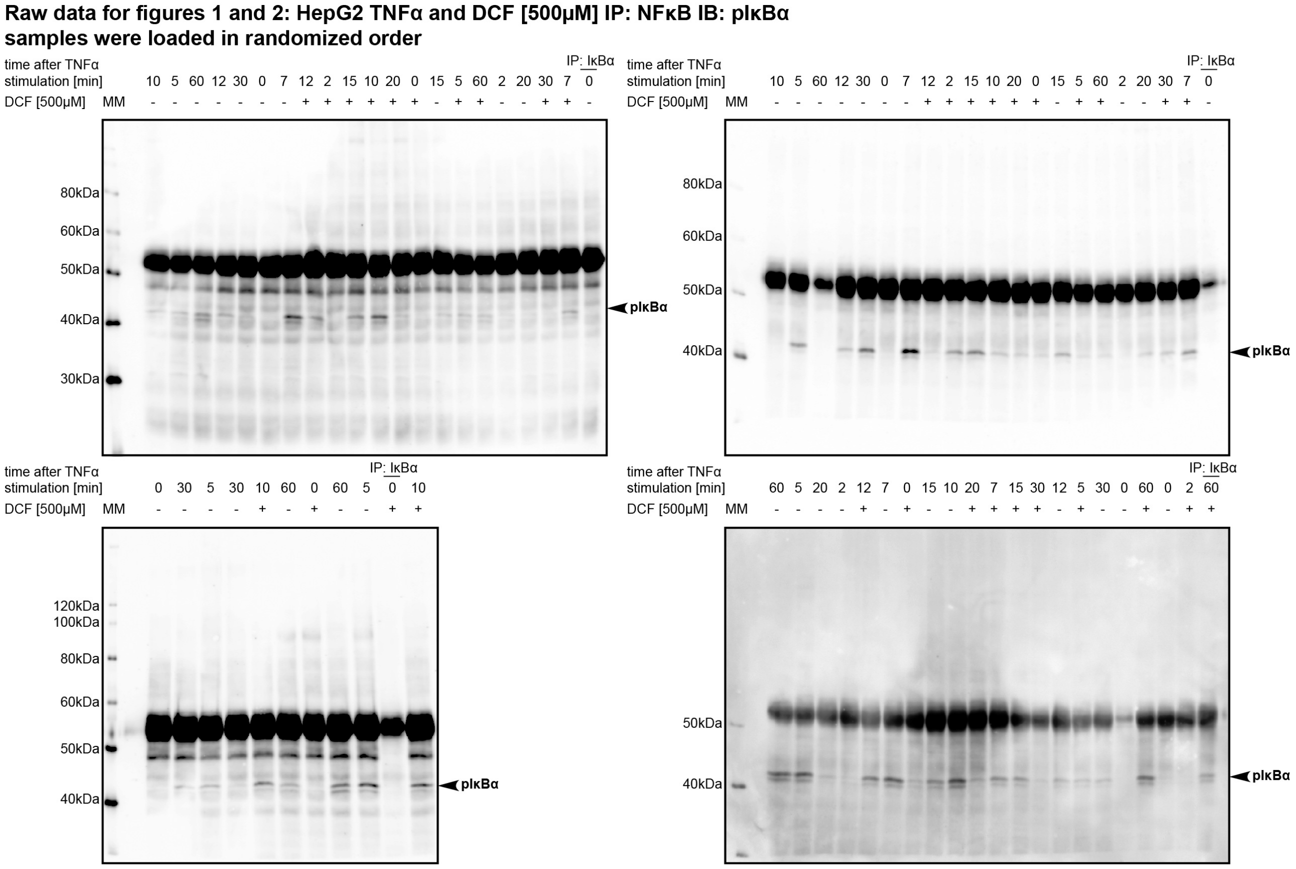

Figure S27: NFκB in Co-IP.

Immunoblots for figures 1E and 2C: HepG2 TNFα and DCF [500µM] IP: NFκB IB: pNFκB samples were loaded in randomized order

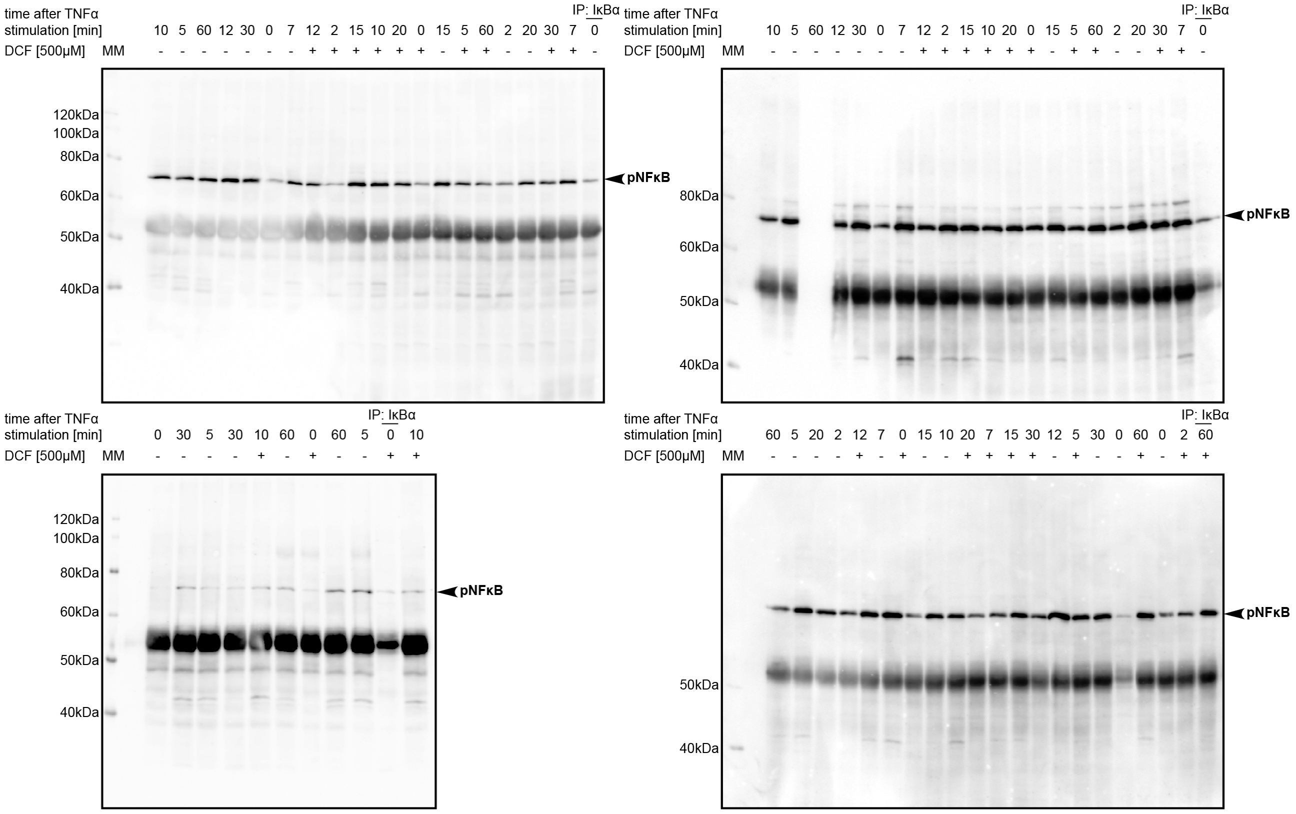

Figure S28: NFκB in Co-IP.

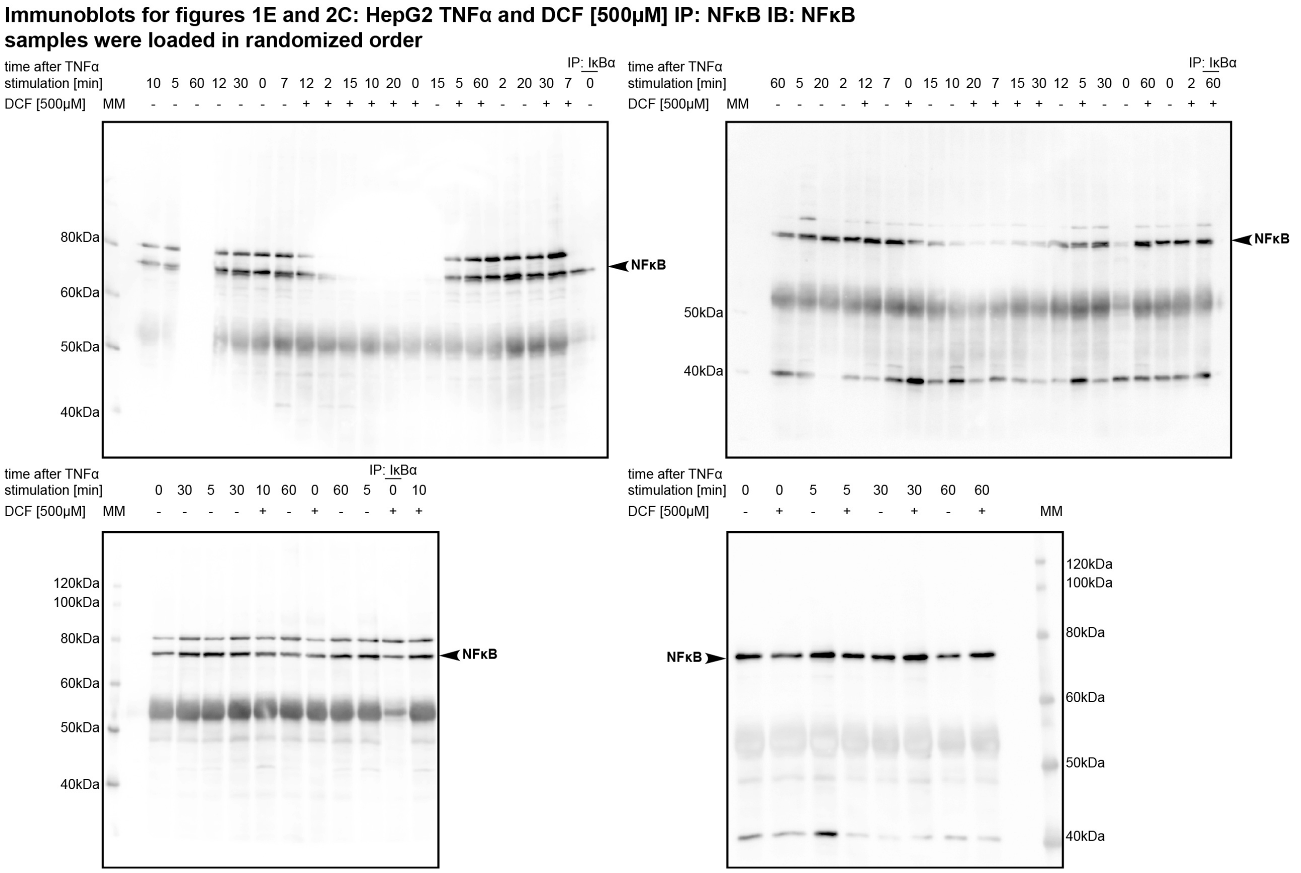

Figure S29: NFκB in Co-IP.

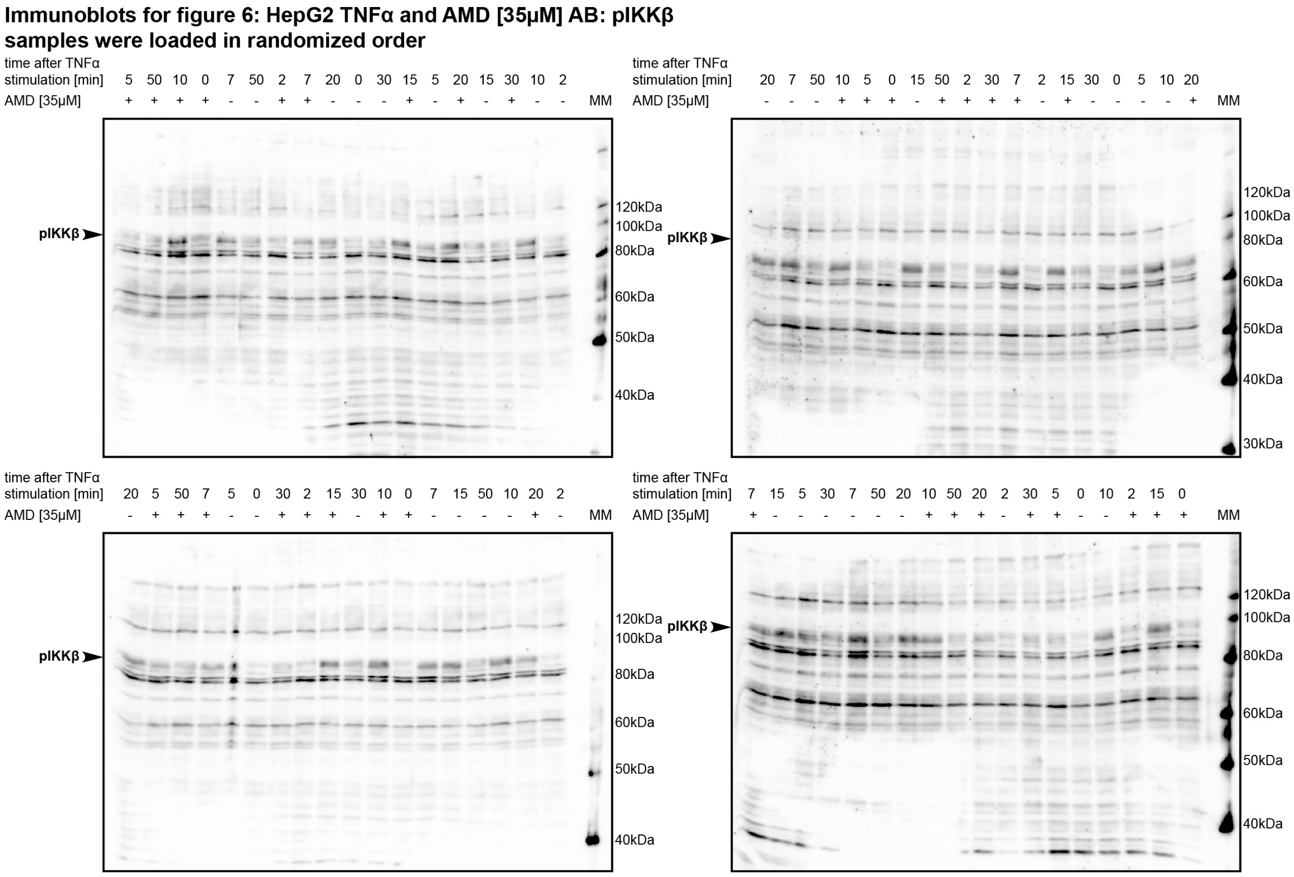

Figure S30: Amiodarone treatment.

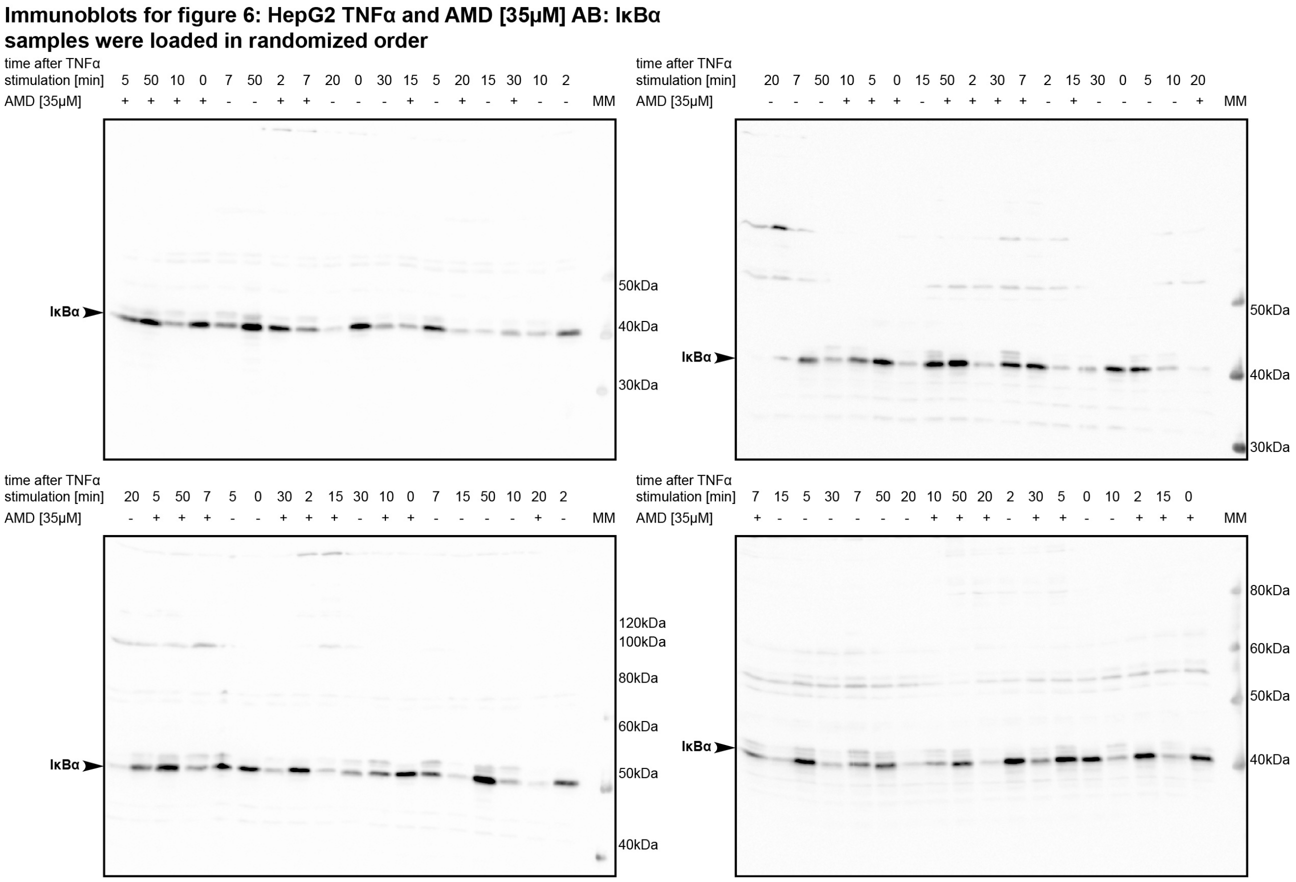

Figure S31: Amiodarone treatment.

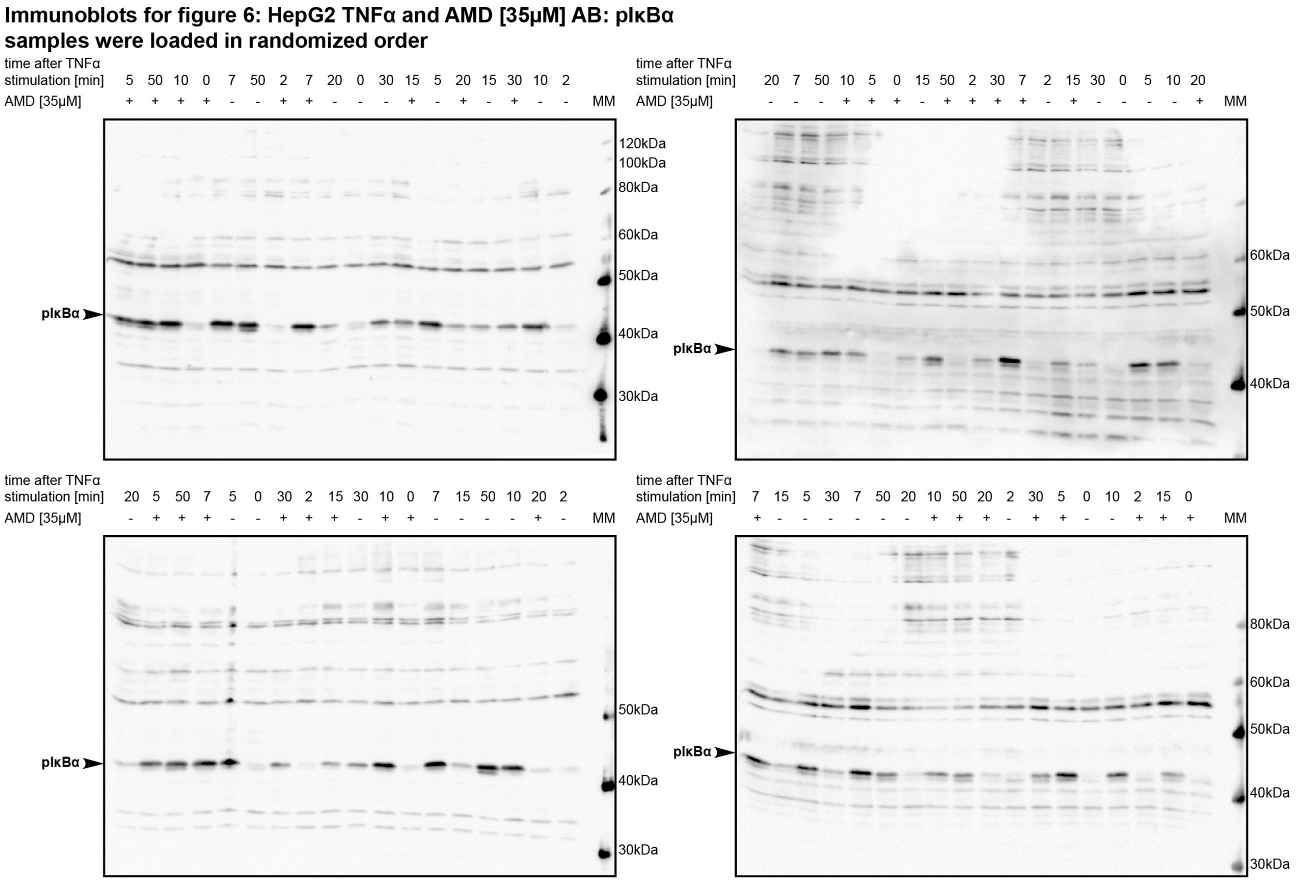

Figure S32: Amiodarone treatment.

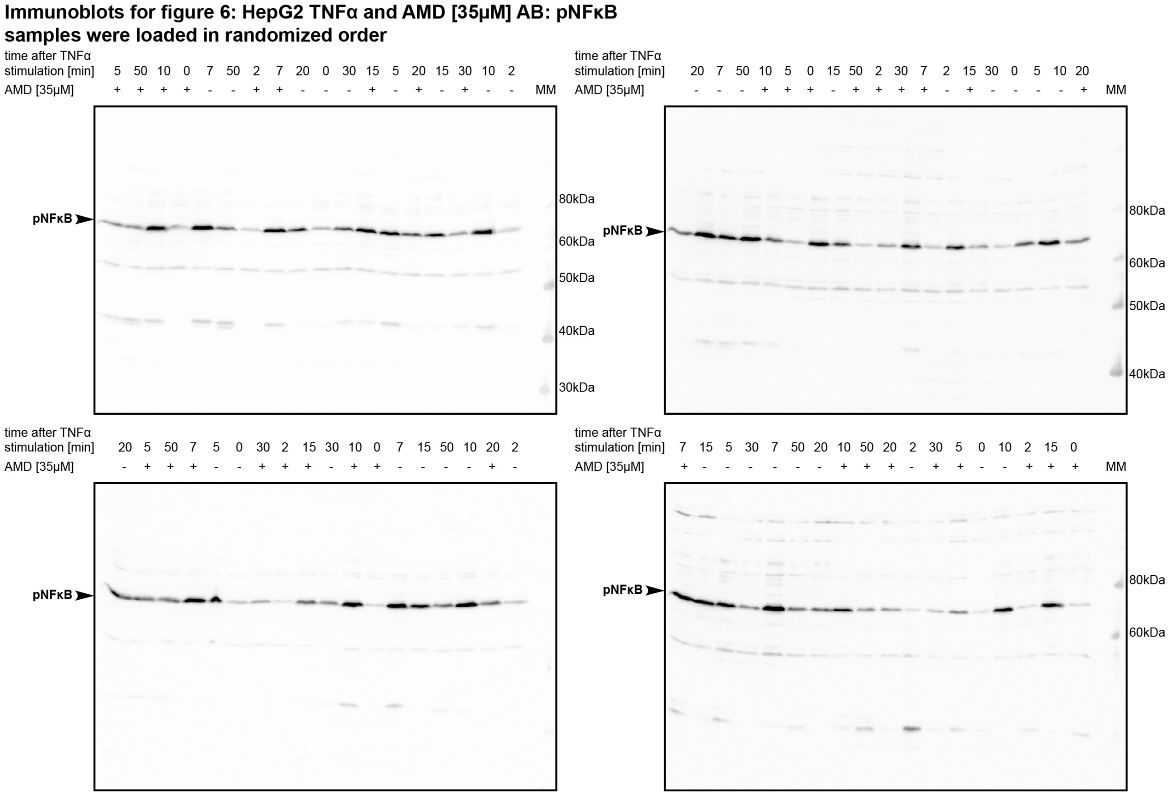

Figure S33: Amiodarone treatment.

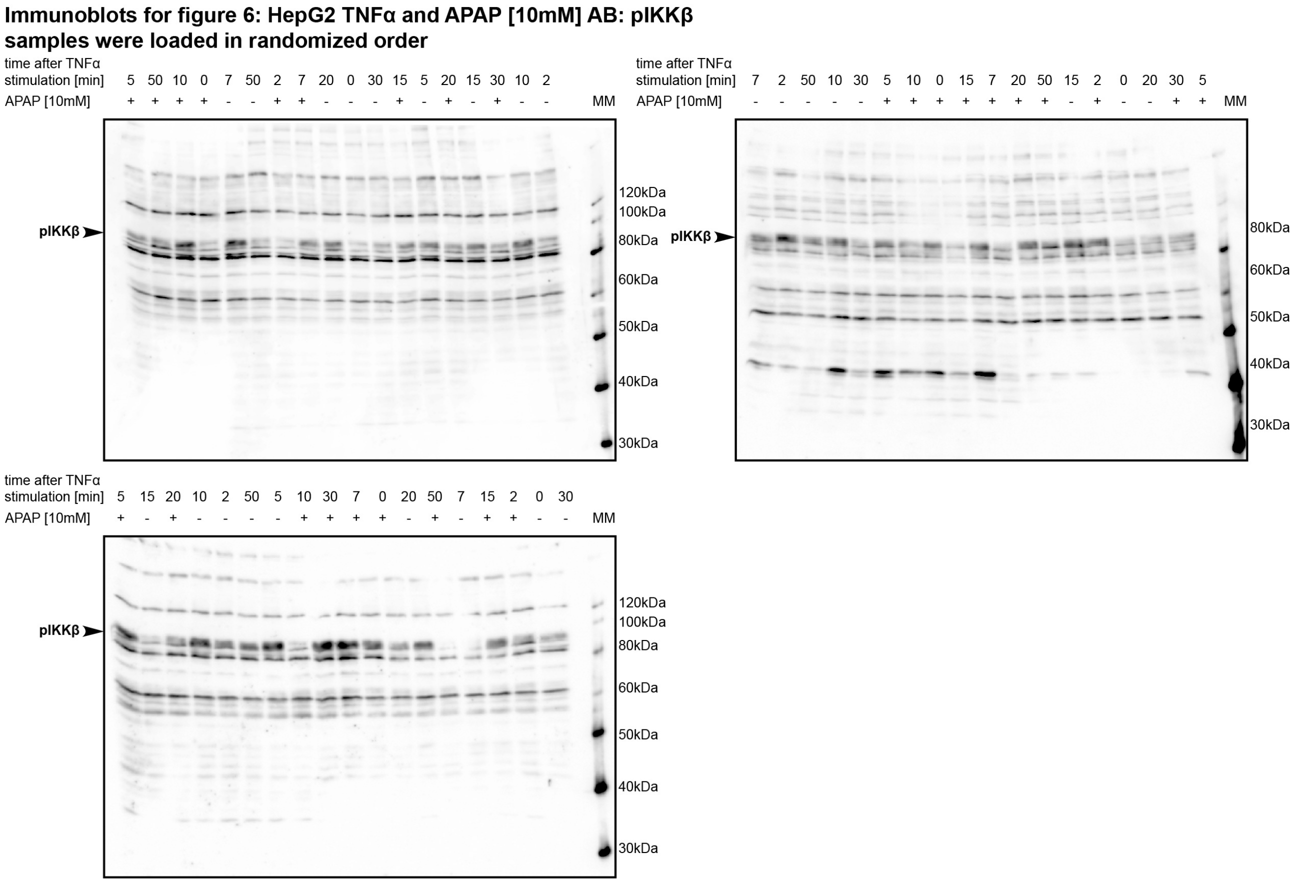

Figure S34: Acetaminophen treatment.

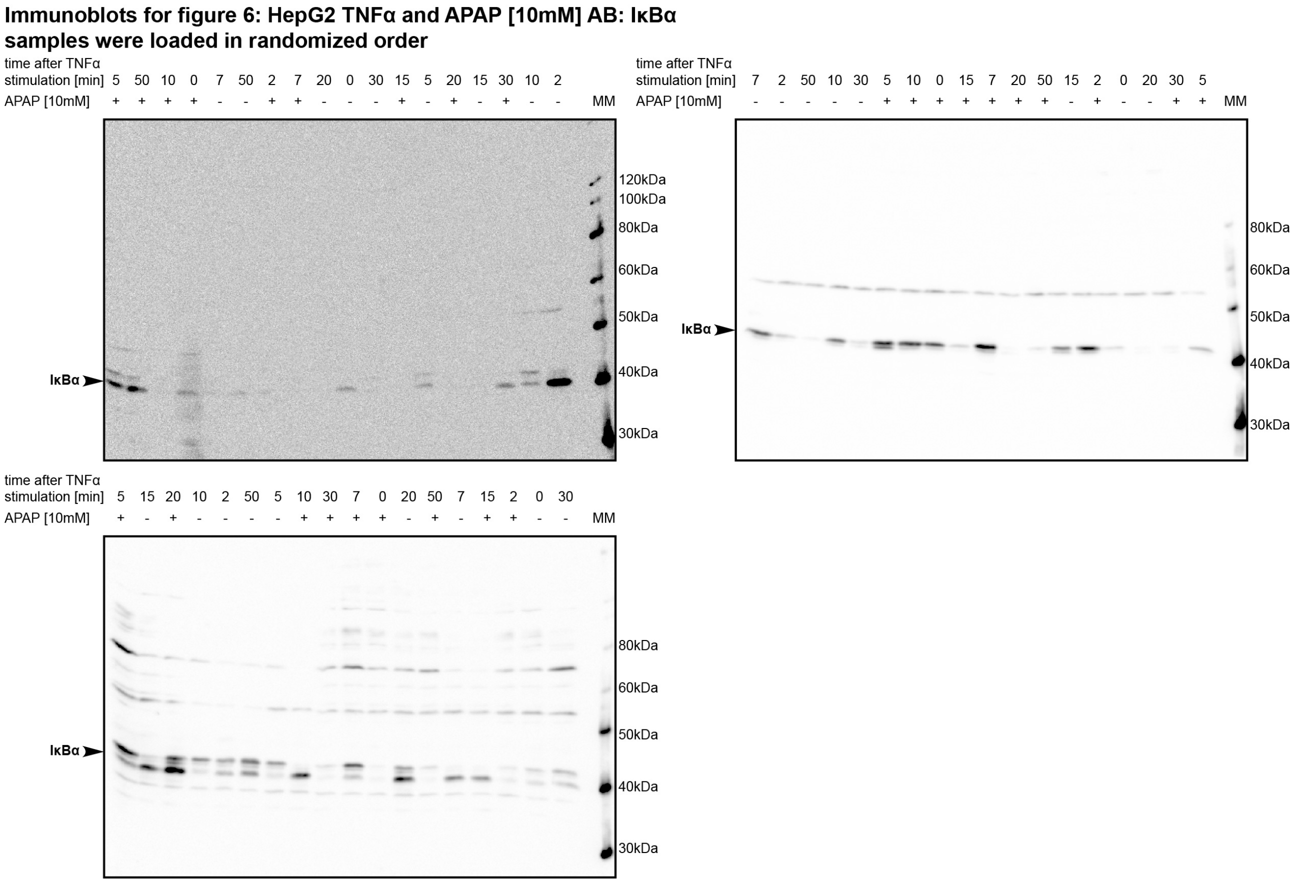

Figure S35: Acetaminophen treatment.

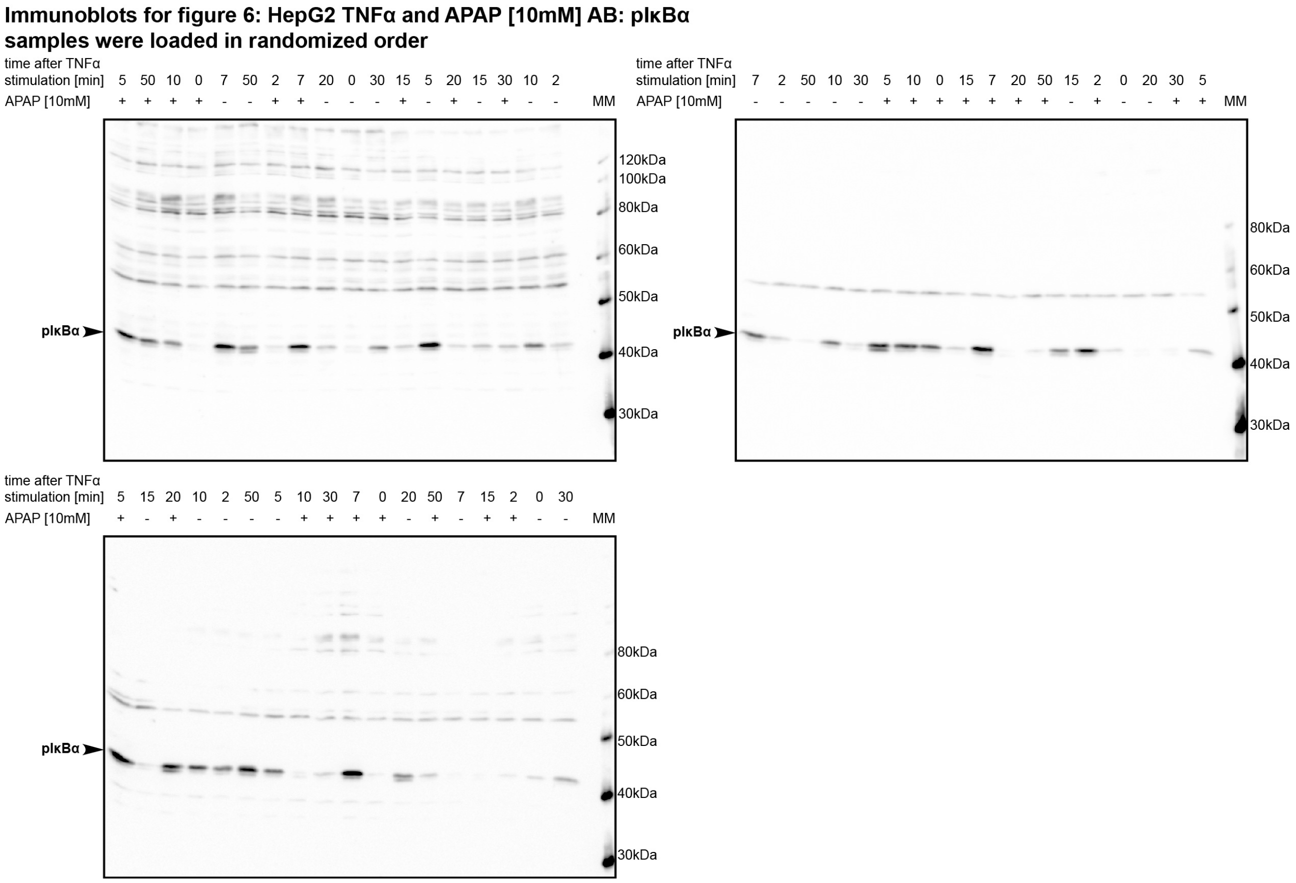

Figure S36: Acetaminophen treatment.

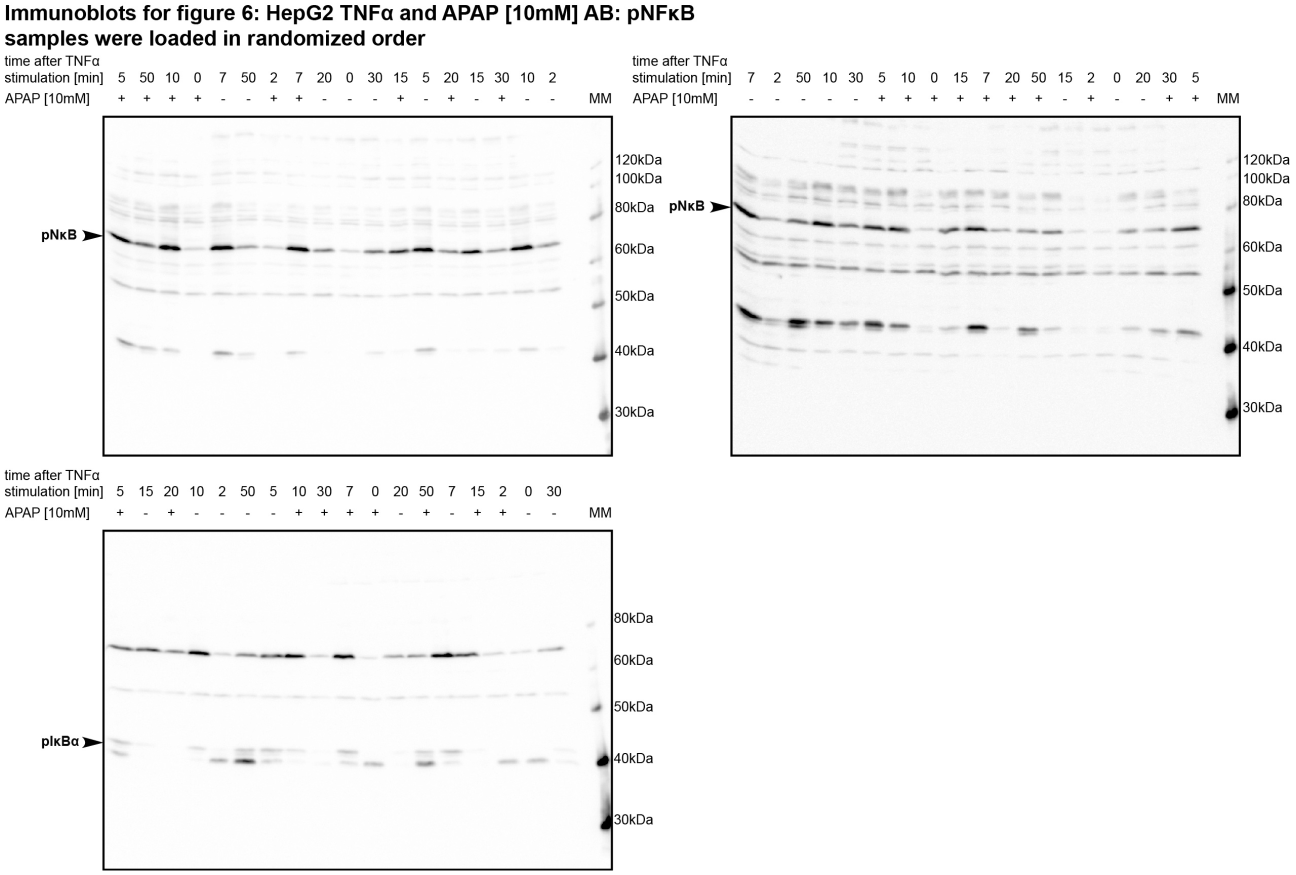

Figure S37: Acetaminophen treatment.

Immunoblots for figure 6: HepG2 TNFα and XIM [800μM] AB: pIKKβ samples were loaded in randomized order

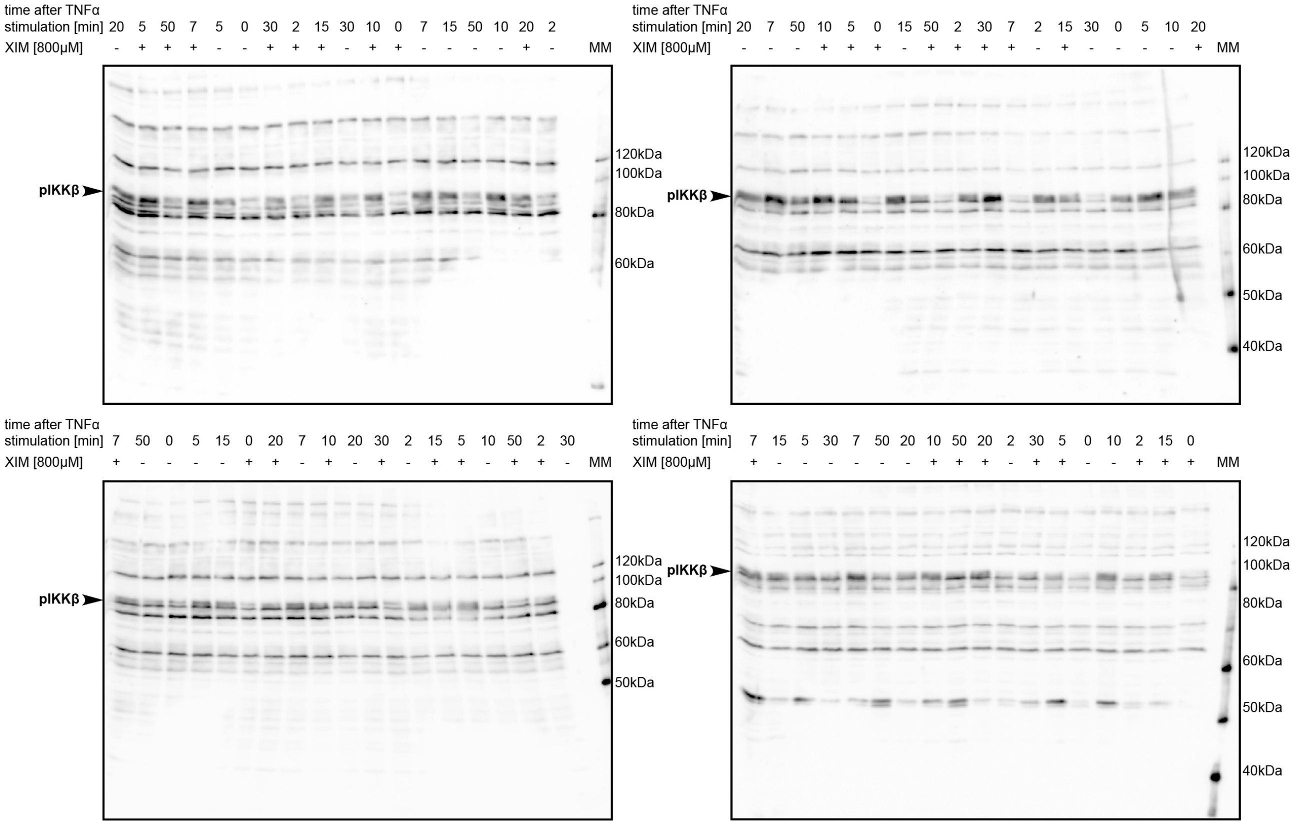

Figure S38: Ximelagatran treatment.

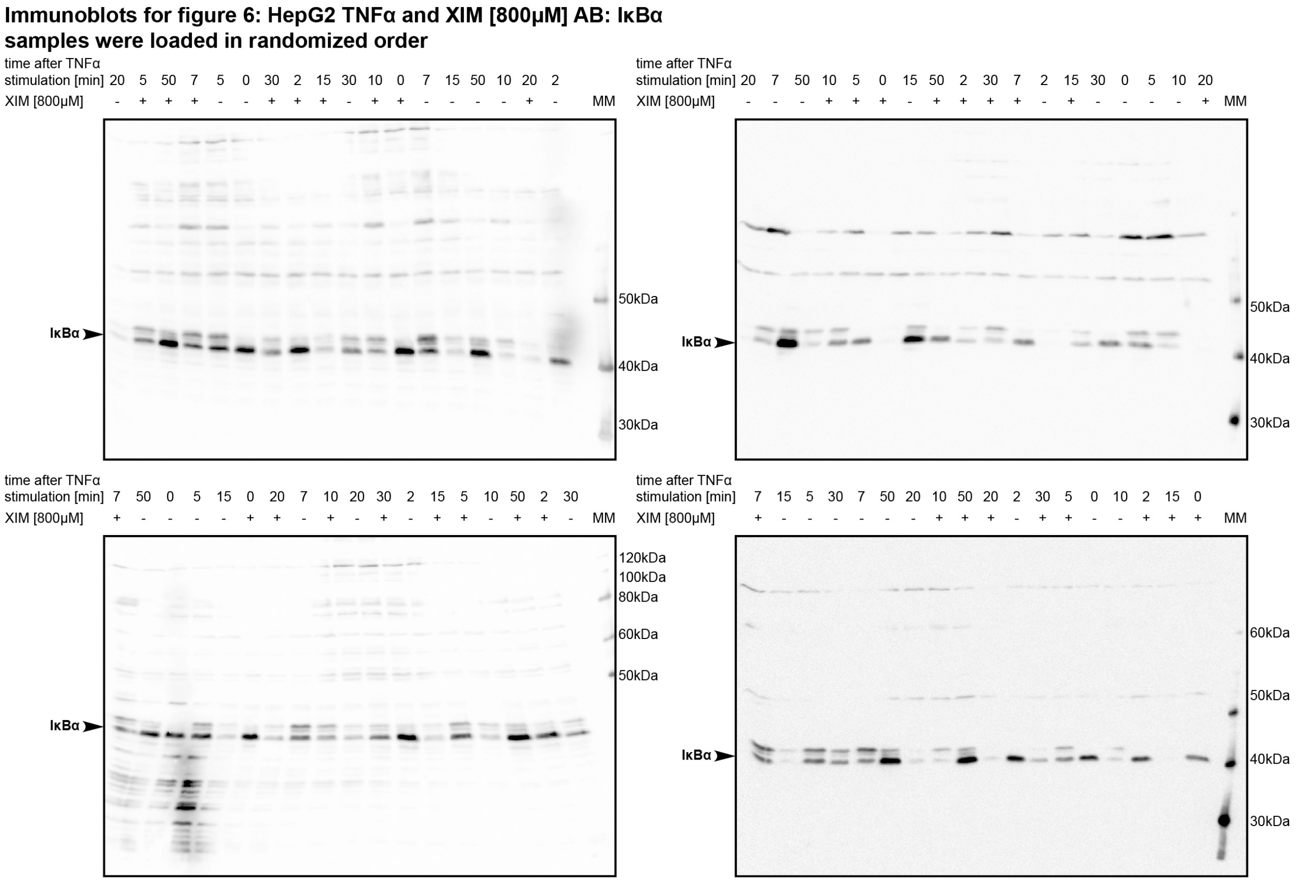

Figure S39: Ximelagatran treatment.

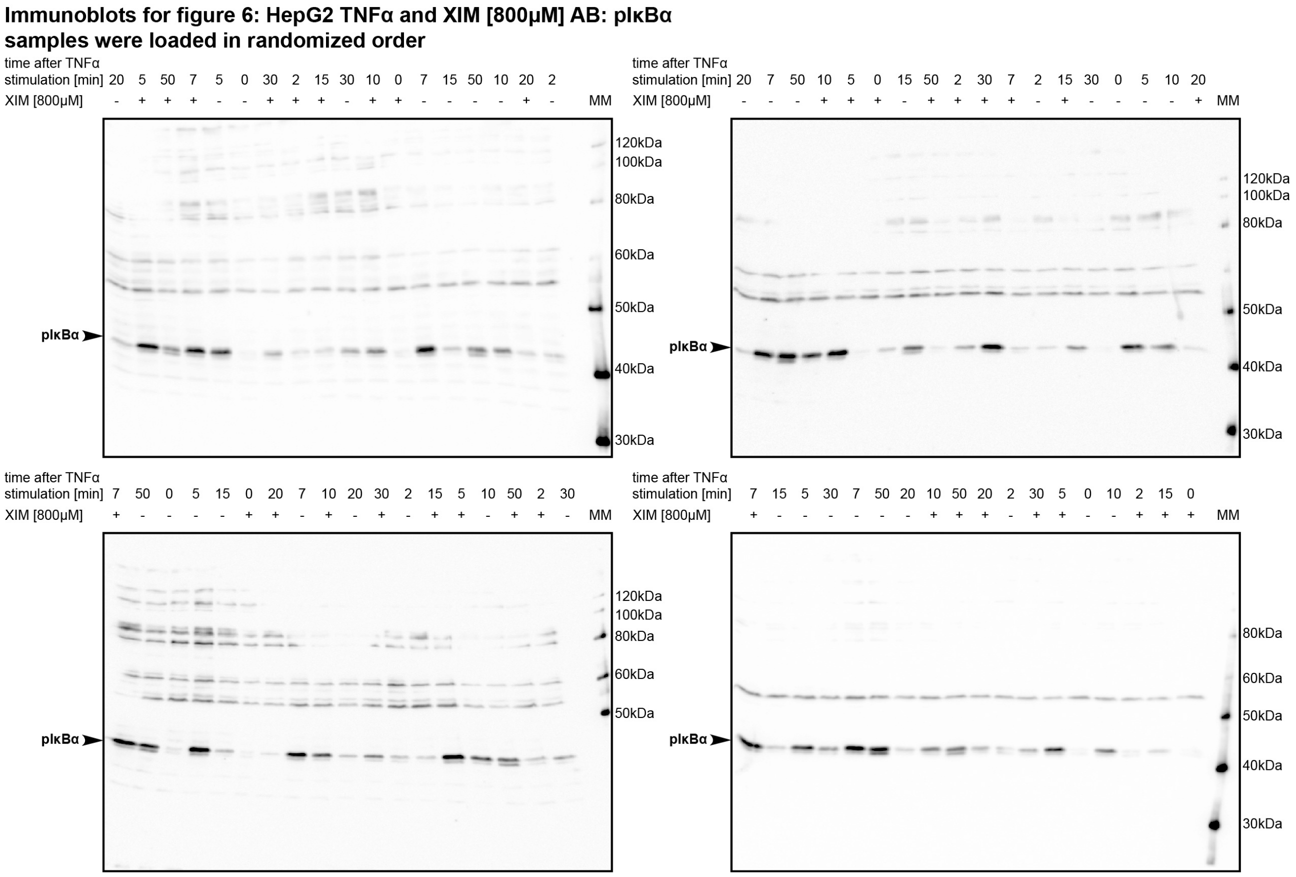

Figure S40: Ximelagatran treatment.

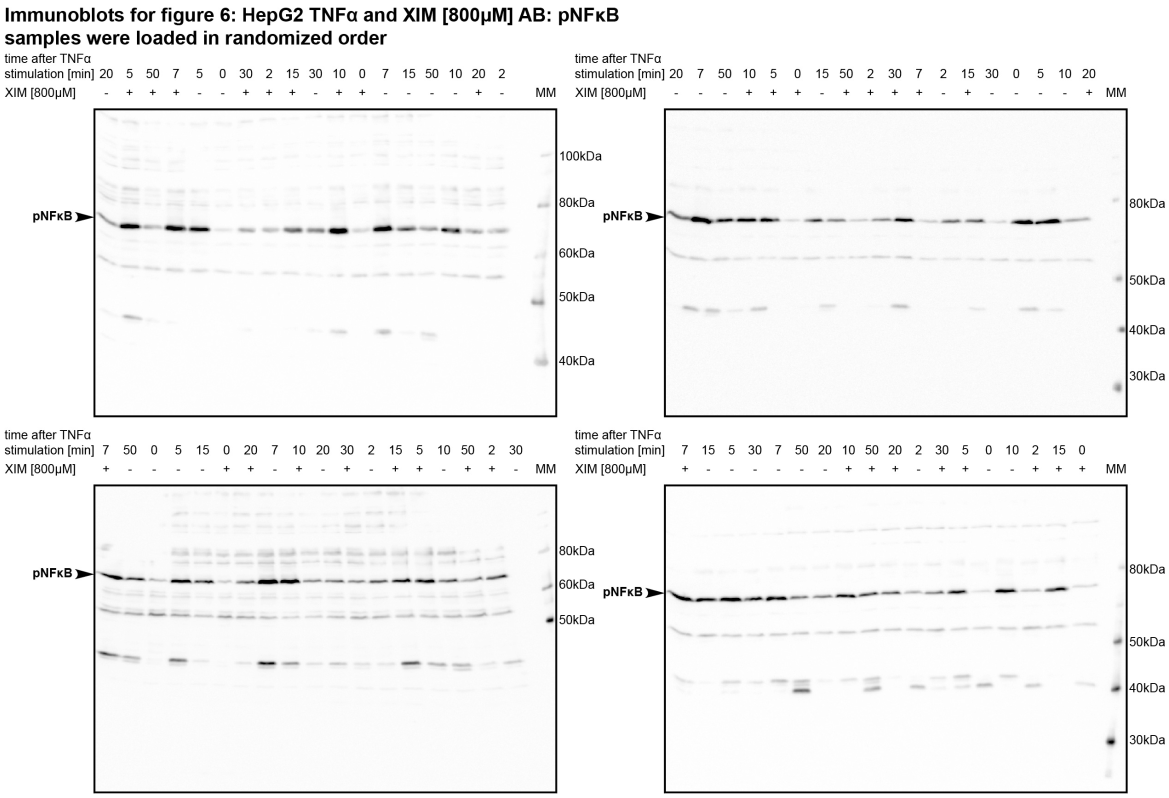

Figure S41: Ximelagatran treatment.

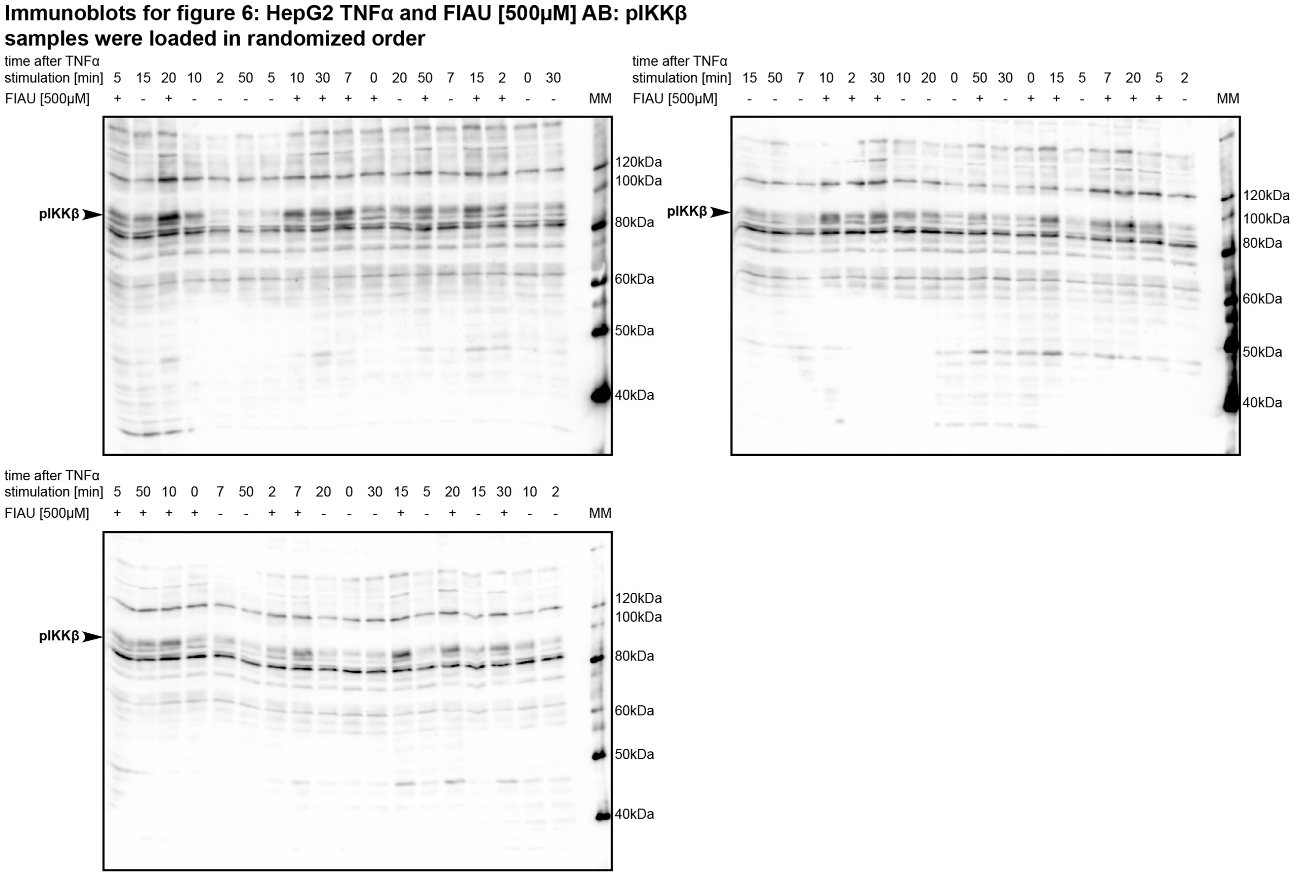

Figure S42: Fialuridine treatment.

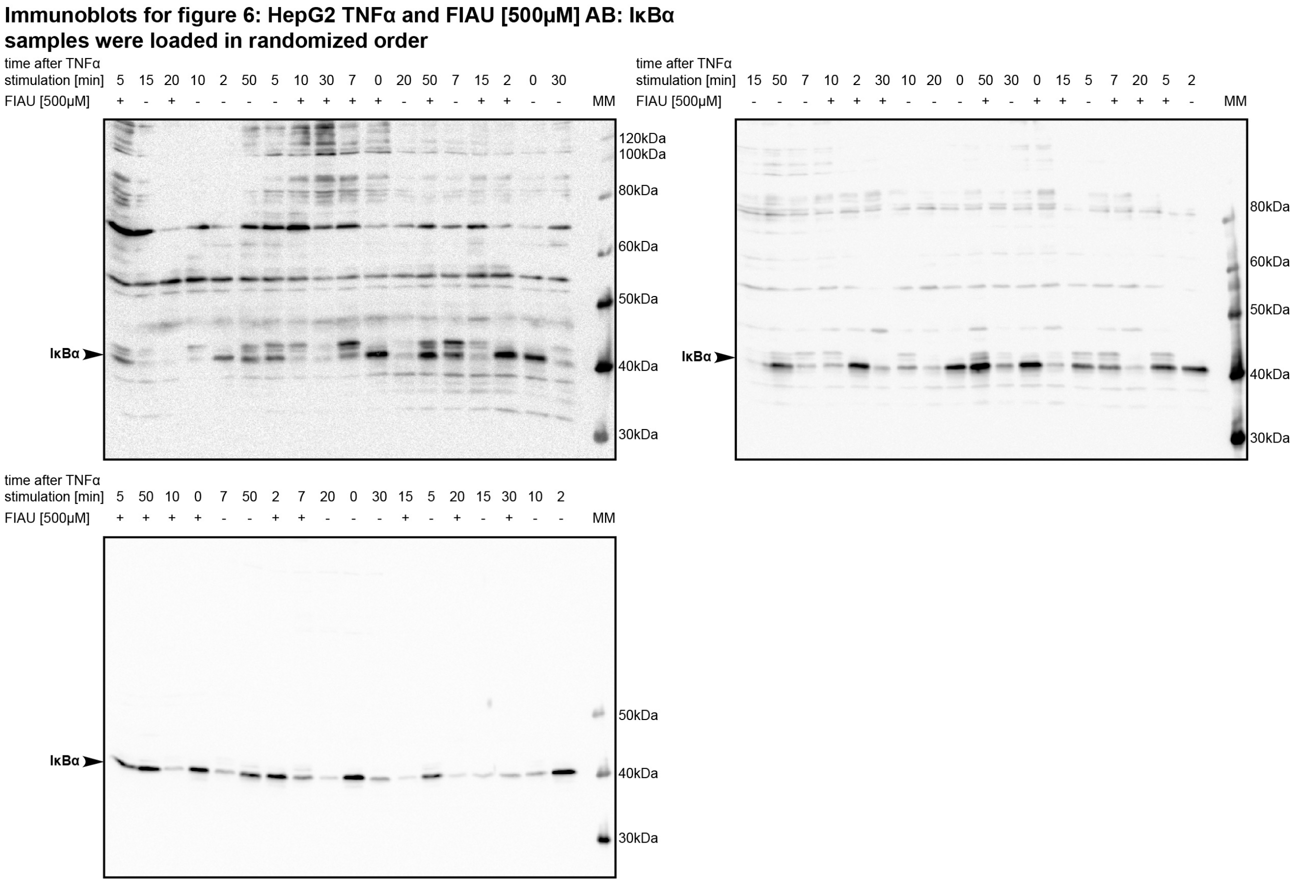

Figure S43: Fialuridine treatment.

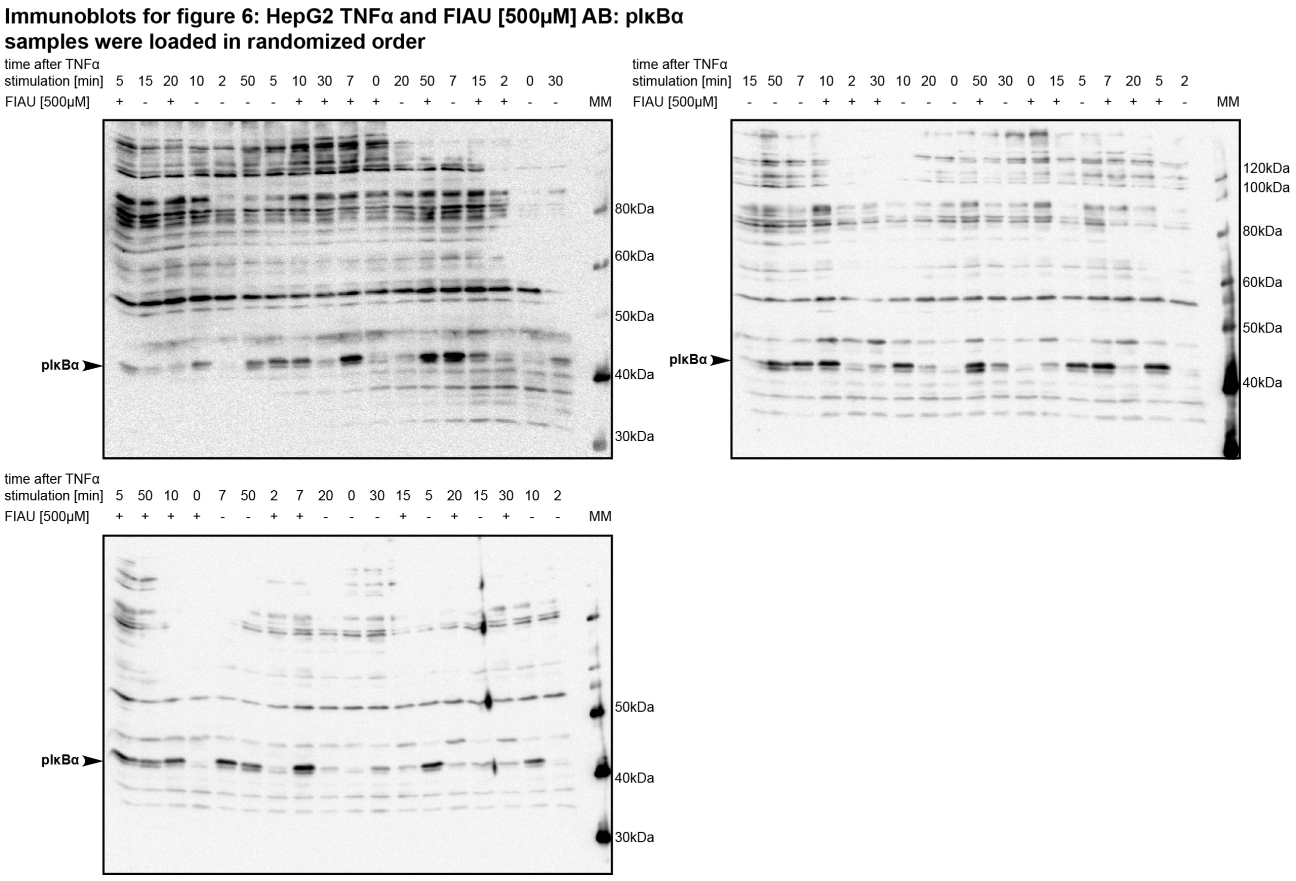

Figure S44: Fialuridine treatment.

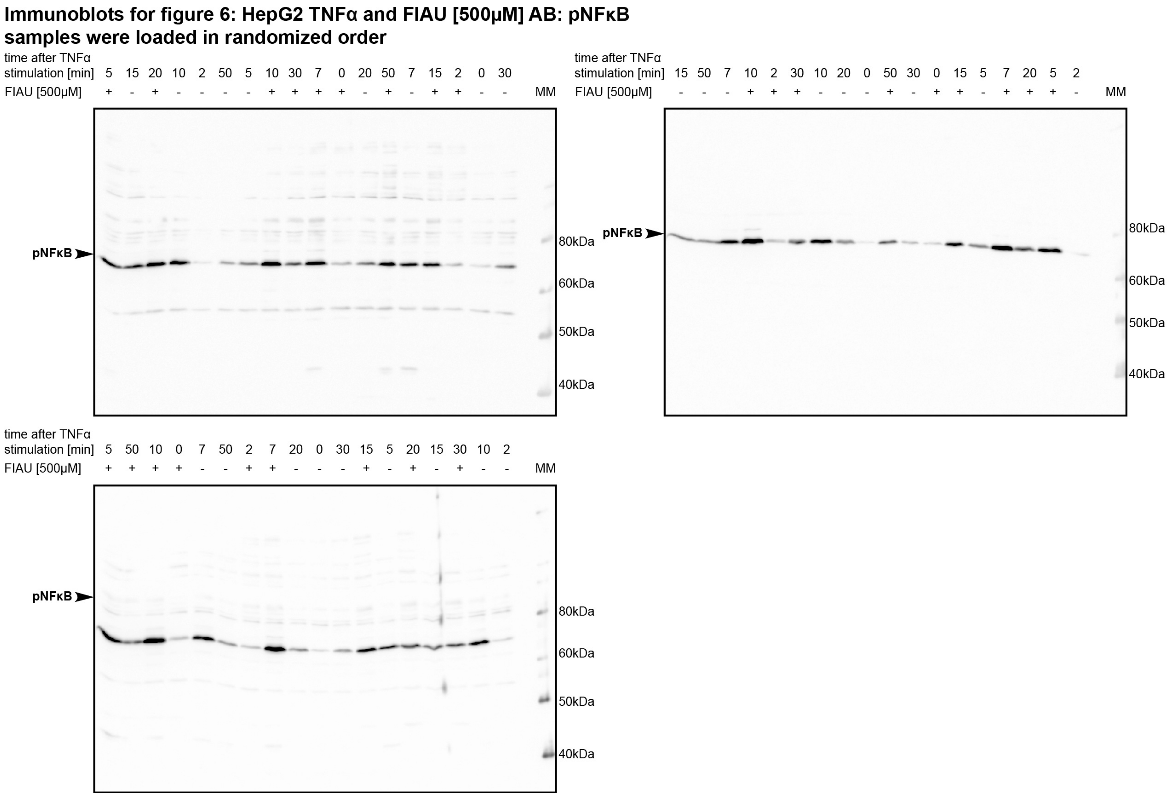

Figure S45: Fialuridine treatment.
